# Spatiotemporal Patterns of Esophageal Cancer Burden Attributable to Behavioral, Metabolic, and Dietary Risk Factors From 1990 to 2019: Longitudinal Observational Study

**DOI:** 10.2196/46051

**Published:** 2023-10-06

**Authors:** Peng Li, Jing Jing, Wenjun Liu, Jizhao Wang, Xin Qi, Guangjian Zhang

**Affiliations:** 1 Department of Thoracic Surgery The First Affiliated Hospital of Xian Jiaotong University Xi'an China; 2 Department of Epidemiology and Health Statistics School of Public Health Xi’an Jiaotong University Health Science Center Xi'an China; 3 College of Geography and Environment Baoji University of Arts and Sciences Baoji China; 4 Department of Cardiovascular Medicine The First Affiliated Hospital of Xian Jiaotong University Xi'an China

**Keywords:** esophageal cancer, age-standardized mortality rate, disability-adjusted life years, attributable risk, average annual percentage change

## Abstract

**Background:**

Esophageal cancer (EC) is the sixth leading cause of cancer-related burden with distinct regional variations globally. Although the burden of EC has decreased, the specific reasons for this decline are still unclear.

**Objective:**

This study aims to uncover the spatiotemporal patterns of EC risk–attributable burden in 204 countries and territories from 1990 to 2019 so that prevention and control strategies of EC can be prioritized worldwide.

**Methods:**

We extracted EC risk–attributable deaths, disability-adjusted life years (DALYs), age-standardized mortality rates (ASMRs), and age-standardized DALY rates (ASDRs) from the global burden of disease (GBD) study from 1990 to 2019, in terms of behavioral, metabolic, and dietary factors by age, sex, and geographical location. Average annual percentage change (AAPC) was used to assess the long-term trends in the ASMRs and ASDRs of EC due to specific risk factors.

**Results:**

Between 1990 and 2019, the greatest decrease in EC burden was attributed to low intake of fruits and vegetables. An AAPC of –2.96 (95% CI –3.28 to –2.63) and –3.12 (95% CI –3.44 to –2.79) in ASMR and ASDR was attributable to a low-fruit diet, while an AAPC of –3.60 (95% CI –3.84 to –3.36) and –3.64 (95% CI –3.92 to –3.35) in ASMR and ASDR was attributed to a low-vegetable diet. However, the trends in ASMRs and ASDRs due to high BMI showed significant increases with an AAPC of 0.52 (95% CI 0.29-0.75) in ASMR and 0.42 (95% CI 0.18-0.66) in ASDR from 1990 to 2019 compared to significant decreases in other attributable risks with AAPC<0 (*P*<.05). East Asia had the largest decrease in EC burden due to low-vegetable diets, with an AAPC of –11.00 (95% CI –11.32 to –10.67) in ASMR and –11.81 (95% CI –12.21 to –11.41) in ASDR, followed by Central Asia, whereas Western Sub-Saharan Africa had the largest increase in ASMR and ASDR due to high BMI, with an AAPC of 3.28 (95% CI 3.14-3.42) and 3.09 (95% CI 2.96-3.22), respectively. China had the highest EC burden attributed to smoking, alcohol use, high BMI, and low-fruit diets. Between 1990 and 2019, there was a significant decrease in EC burden attributable to smoking, alcohol use, chewing tobacco, low-fruit diets, and low-vegetable diets in most countries, wherein a significant increase in the EC burden was due to high BMI.

**Conclusions:**

Our study shows that smoking and alcohol consumption are still the leading risk factors of EC burden and that EC burden attributable to low intake of fruits and vegetables has shown the largest decline recently. The risks of ASMRs and ASDRs of EC showed distinct spatiotemporal patterns, and future studies should focus on the upward trend in the EC burden attributed to high BMI.

## Introduction

In 2019, esophageal cancer (EC) was the sixth leading cause of cancer deaths and disability-adjusted life years (DALYs), with over 0.54 million deaths and 1.17 million DALYs, representing 50% of all cancers worldwide [1]. Early diagnosis and treatment have been implemented to reduce EC incidence and mortality rates and to improve survival rates over years. However, patients with EC still have low 5-year survival rates (about 30.3%) due to unclear and nonspecific early clinical symptoms, and most patients are diagnosed at an advanced stage [2]. Therefore, EC is still an important public health and clinical challenge for early diagnosis and prevention, especially in low-income countries. Besides, EC burden greatly varies with geographical locations, and the “EC belt” has substantially high EC incidence and mortality rates worldwide [3]. The disparities in the burden of cancer and the poor treatment outcomes across regions worldwide are reflected in the Sustainable Development Goal target 3.4 that focuses on reducing premature mortality from noncommunicable diseases (eg, cancers) by one-third by promoting mental health and well-being by 2030 through prevention and treatment [4].

EC has 2 main histological subtypes, that is, esophageal squamous cell carcinoma (ESCC) and esophageal adenocarcinoma (EAC), which have analogous and distinct etiologies with modifiable risk factors (eg, smoking, alcohol use, obesity, diet) [5,6]. EC cases with over 85% ESCC histological subtype cluster in low-income countries and has recently shown a significantly downward trend worldwide [7,8]. Previous studies have reported the epidemiological characteristics of EC, such as incidence, mortality, and DALY rates in several countries or regions over several years [9,10]. Age-standardized incidence rates of EC in China have declined significantly from 1990 to 2019, and the decrease is more pronounced among females than among males [11]. Moreover, there are significant differences in the risk factors between sexes [11]. There was a significant male predominance in EC cases with a male-to-female incidence ratio of 3.3:1 for ESCC and 6.7:1 for EAC [8]. Further, the interaction effect of sex and smoking in ESCC has been reported in a cohort study with 13 years of median follow-up [12]. However, comprehensive understanding of EC risk–attributable burden does not exist in many countries, leading to a void in the up-to-date prevention strategies and adjustment in the EC treatment research focus. At this point, the latest changes in the specified risk-attributable burden of EC should be scheduled at global, regional, and national scales to elucidate the trend in EC cases that are attributable to primary factors (eg, smoking, alcohol use, metabolism, diet). Addressing the primary factors contributing to EC burden can lead to advances in the prognosis and treatment of EC in countries worldwide that require EC prevention and control measures.

To date, the Global Burden of Disease (GBD) study has assessed cancer burden by broadly considering the major modifiable risk factors across countries, ages, and sexes worldwide over time. To our knowledge, GBD is the only database that quantifies and provides a comprehensive picture of the cancer burden attributable to a set of risk factors through stringent quality assurance procedures to model at different scales over time. Further, GBD 2019 provides the latest information on each risk-outcome pair for EC in 204 countries and territories and its aggregation regions from 1990 to 2019, which enables the evaluation of the risk-attributable burden for low-income countries. Herein, we analyzed the spatiotemporal patterns in the burden of EC attributable to specific behavioral, metabolic, and dietary risk factors at global, regional, and national levels over 3 decades, which can help policy makers implement prevention measures and tackle the growing EC burden driven by specific risk factors.

## Methods

### Data Source

We collected the annual number of EC (International Classification of Diseases 10th revision code C15) deaths, DALYs, age-standardized mortality rates (ASMRs), and age-standardized DALY rates (ASDRs) related to risk-attributable factors by sex and age at global, regional, and national levels from GBD 2019 estimations from 1990 to 2019. The data included EC risk–attributable deaths, DALYs, ASMRs, and ASDRs in 204 countries and territories and were clustered in 21 GBD regions according to geographical proximity and economic similarities [13]. Subsequently, according to the sociodemographic index (SDI) ranging from 0 to 1, 204 countries and territories were classified under 5 SDI levels: low SDI (<0.45), low-middle SDI (≥0.45 to <0.61), middle SDI (≥0.61 to <0.69), high-middle SDI (≥0.69 to <0.80), and high SDI (≥0.80). The SDI is a composite indicator of a country’s lag-distributed income per capita, average years of schooling (aged >15 years), and fertility rate in females younger than 25 years [14].

### Exposure Selection and Definition

By reviewing previous literature and studies in terms of the leading risk factors of EC burden, we targeted the EC burden attributable to the most prominent risk factors, including behavioral risk (ie, smoking, alcohol use, and chewing tobacco), metabolic risk (ie, high BMI), and diet risk (ie, diet with less vegetables and fruits) [15,16]. We focused on these leading factors (established EC risk factors with available data in GBD) to analyze the spatiotemporal patterns of EC burden in different regions in order to implement EC prevention and intervention measures. In this study, smoking is defined as the prevalence of current use of any smoked tobacco product and prevalence of the former use of any smoked tobacco product. Alcohol use is defined as the average daily alcohol consumption of pure alcohol (measured in grams per day) by current drinkers who had consumed alcohol during the past 12 months. Chewing tobacco is defined as the current use of any chewing tobacco product. High BMI is defined as BMI above the normal range (18.5-24.9 kg/m^2^). Diet low in vegetables and fruits is defined as the average daily consumption of vegetables and fruits (measured in grams per day). These variables are well-established risk factors for EC with available data in GBD and whose detailed definitions and risk gradients for EC are elaborated elsewhere [17]. Because EC incidence and mortality have been hardly reported among individuals younger than 40 years [18], we considered 0-39 years old as one age group, individuals aged 40-94 years as smaller subgroups each with an age range of 5 years, and >95 years old as the last age group.

### Statistical Analysis

#### Estimation of the EC Burden

Data on deaths, DALYs, ASMRs, and ASDRs were reported with numbers and their 95% uncertainty intervals (UIs), generating values at the 2.5th and 97.5th percentile from 1000 draws. The methods for the estimation of explaining uncertainty in the risk-attributable burden have been described in detail elsewhere [15]. Briefly, the GBD comparative risk assessment was divided into 6 key processes for each risk-outcome estimation. First, we identified convincing or probable evidence for causal association with EC based on systematic reviews and meta-regression. Second, we estimated the relative risks for each risk-outcome pair as risk-attributable function of EC by using GBD’s meta-regression-Bayesian, regularized, and trimmed method. Third, we analyzed the model risk exposure level and distribution by age, sex, location, and year by using Bayesian meta-regression modelling (DisMod-MR 2.1). Fourth, we determined the theoretical minimum risk exposure level based on a counterfactual scenario (a given population receives the optimal level of risk exposure) and the 85th percentile of exposure in cohorts and trial studies. Fifth, we calculated the population attributable fractions for each risk-outcome pair by age, sex, location, and year. Sixth, we estimated the EC burden attributable to specified risk factors, wherein deaths and DALYs were multiplied by the corresponding risk factor population attributable fraction [19,20].

#### Analysis of the Time Trends of EC Burden

An average annual percentage change (AAPC) was used to estimate the long-term trends in ASMR and ASDR in EC cases attributable to each risk factor from 1990 to 2019. In this study, AAPC was widely used to analyze a summary measure of time trend over an entire period, which was fitted in a regression by the natural logarithm of the ASMR and ASDR from 1990 to 2019, that is, y = α+βx+ɛ, where y = ln (ASMR or ASDR) and x = calendar year. The AAPC was calculated as 100 × (exp(β)-1), and its 95% CI can also be obtained from the fitted regression model. The time trend of ASMR or ASDR was defined as a significant increase if AAPC>0 and its lower boundary of 95% CI>0 (*P*<.05). Inversely, the time trend of ASMR or ASDR was defined as a significant decrease if the AAPC<0 and its upper boundary of 95% CI<0 (*P*<.05). Otherwise, it was not a significant trend over time. In addition, we assessed the time trends of ASMR and ASDR attributable to each specific risk factor for EC by age group and sex at regional, SDI, and national levels from 1990 to 2019. These statistical analyses were conducted using Joinpoint trend analysis software (version 4.9.1.0; Statistical Methodology and Applications Branch), and corresponding geographical patterns were presented by ArcGIS 10.6 (ESRI). A *P* value less than .05 was considered statistically significant.

### Ethical Considerations

This study did not involve human participants and animals. Ethics approval was not applicable for this study, as this study used existing good quality modeled data that were aggregated at the population level.

## Results

### Global Trends in EC Risk–Attributable Burden

As Table 1 shows, although the total number of EC risk–attributable deaths and DALYs globally increased in both sexes combined between 1990 and 2019, the corresponding ASMRs and ASDRs decreased. The deaths attributable to smoking increased from 134,682 (95% UI 105,311-152,927) in 1990 to 203,328 (95% UI 170,461-236,522) in 2019, while the corresponding ASMR decreased from 3.42 (95% UI 2.69-3.86) per 100,000 people in 1990 to 2.48 (95% UI 2.08-2.89) per 100,000 people in 2019. The DALYs and ASDRs attributable to smoking followed a similar pattern during the same period. Besides, in 2019, the higher global EC burden was attributable to specifically behavioral, metabolic, and dietary factors among males (Table 1).

**Table 1:** Global burden of esophageal cancer attributable to behavioral, metabolic, and diet factors in 1990 and 2019.

Risk factor		1990				2019			
		Death (persons, 95% UI^a^)	ASMR^b^ (95% UI)	DALY^c^ (95% UI)	ASDR^d^ (95% UI)	Death (persons, 95% UI)	ASMR (95% UI)	DALY (95% UI)	ASDR (95% UI)
**Smoking**									
	B^e^	134,682 (105,311-152,927)	3.42 (2.69-3.86)	3,475,304 (2,674,422-3,967,053)	84.38 (65.34-96.30)	203,328 (170,462-236,512)	2.48 (2.08-2.89)	4,746,524 (3,983,523-5,544,229)	56.71 (47.57-66.14)
	M^f^	120,286 (92,296-137,575)	6.70 (5.22-7.62)	3,160,591 (2,404,539-3,635,360)	162.14 (123.89-186.04)	187,234 (156,028-219,347)	4.95 (4.12-5.78)	4,425,432 (3,688,962-5,188,276)	111.18 (92.75-130.29)
	F^g^	14,396 (11,141-17,351)	0.69 (0.53-0.83)	314,713 (242,655-382,305)	14.73 (11.41-17.87)	16,094 (12,861-19,289)	0.37 (0.29-0.44)	321,092 (260,159-383,914)	7.33 (5.94-8.76)
**Alcohol use**									
	B	65,669 (47,318-84,125)	1.64 (1.19-2.11)	1,792,113 (1,277,343-2,281,636)	42.97 (30.73-54.66)	113,600 (84,063-144,686)	1.38 (1.02-1.76)	2,818,188 (2,109,628-3,573,631)	33.55 (25.09-42.54)
	M	58,665 (41,912-74,762)	3.17 (2.30-4.04)	1,628,732 (1,160,091-2,068,043)	81.57 (58.12-103.62)	103,883 (77,092-132,860)	2.71 (2.01-3.46)	2,609,021 (1,943,902-3,312,769)	64.84 (48.32-82.39)
	F	7004 (4724-9732)	0.34 (0.23-0.47)	163,381 (110,272-227,901)	7.62 (5.15-10.61)	9718 (6701-13,143)	0.22 (0.15-0.30)	209,167 (145,394-281,635)	4.80 (3.33-6.46)
**Chewing tobacco**									
	B	9398 (6604-12,589)	0.24 (0.17-0.31)	261,073 (182,523-351,920)	6.25 (4.38-8.40)	18,277 (12,696-24,652)	0.22 (0.15-0.30)	475,862 (327,634-643,826)	5.66 (3.91-7.66)
	M	6463 (4059-9238)	0.35 (0.22-0.50)	184,101 (115,166-263,107)	9.16 (5.75-13.08)	12,277 (7441-17,718)	0.32 (0.19-0.46)	330,479 (200,616-476,315)	8.14 (4.94-11.74)
	F	2935 (1797-4434)	0.14 (0.08-0.21)	76,972 (46,998-116,401)	3.57 (2.18-5.38)	6000 (3649-8965)	0.14 (0.08-0.21)	145,383 (88,251-216,523)	3.34 (2.03-4.99)
**High BMI**									
	B	35,283 (10,182-75,927)	0.90 (0.26-1.97)	917,929 (256,226-1,976,144)	22.26 (6.29-47.89)	89,904 (27,879-171,255)	1.09 (0.34-2.10)	2,202,314 (681,901-4,173,080)	26.27 (8.12-49.89)
	M	23,273 (3778-54,281)	1.28 (0.21-2.99)	634,186 (103,387-1,476,891)	32.05 (5.21-74.68)	65,511 (12,587-135,689)	1.71 (0.33-3.54)	1,653,597 (320,884-3,422,784)	41.06 (7.95-85.11)
	F	12,010 (640-30,139)	0.58 (0.03-1.44)	283,743 (15,239-714,960)	13.20 (0.71-33.16)	24,393 (1296-53,852)	0.56 (0.03-1.23)	548,718 (29,096-1,198,556)	12.59 (0.67-27.51)
**Diet low in fruits**									
	B	51,867 (17,816-92,689)	1.32 (0.45-2.37)	1,358,518 (473,229-2,387,195)	32.83 (11.40-57.97)	51,210 (15,227-108,734)	0.63 (0.19-1.33)	1,249,775 (384,470-2,595,057)	14.96 (4.60-31.05)
	M	33,861 (11,023-61,135)	1.86 (0.60-3.39)	924,139 (307,556-1,665,445)	46.68 (15.36-83.83)	35,701 (10,018-78,761)	0.94 (0.26-2.09)	892,474 (262,854-1,927,948)	22.31 (6.49-48.36)
	F	18,006 (6627-32,143)	0.86 (0.31-1.53)	434,380 (160,567-761,632)	20.12 (7.43-35.37)	15,509 (5185-30,419)	0.35 (0.12-0.70)	357,301 (128,558-668,342)	8.23 (2.98-15.36)
**Diet low in vegetables**									
	B	21,591 (2291-44,769)	0.55 (0.06-1.15)	550,996 (57,581-1,145,982)	13.41 (1.41-27.88)	17,176 (2549-33,958)	0.21 (0.03-0.42)	420,309 (64,154-827,694)	5.03 (0.77-9.91)
	M	14,339 (1421-29,787)	0.80 (0.08-1.67)	379,595 (37,236-789,676)	19.45 (1.92-40.34)	11,573 (1709-23,211)	0.31 (0.04-0.62)	288,897 (43,912-575,697)	7.23 (1.09-14.42)
	F	7252 (797-14,894)	0.35 (0.04-0.71)	171,401 (19,849-350,757)	7.96 (0.92-16.31)	5603 (868-10,917)	0.13 (0.02-0.25)	131,412 (20,890-258,144)	3.03 (0.48-5.95)

^a^UI: uncertainty interval.

^b^ASMR: age-standardized mortality rate per 100,000 people.

^c^DALY: disability-adjusted life year of persons.

^d^ASDR: age-standardized disability-adjusted life year rate per 100,000 people.

^e^B: both male and female.

^f^M: male.

^g^F: female.

**Figure 1:**
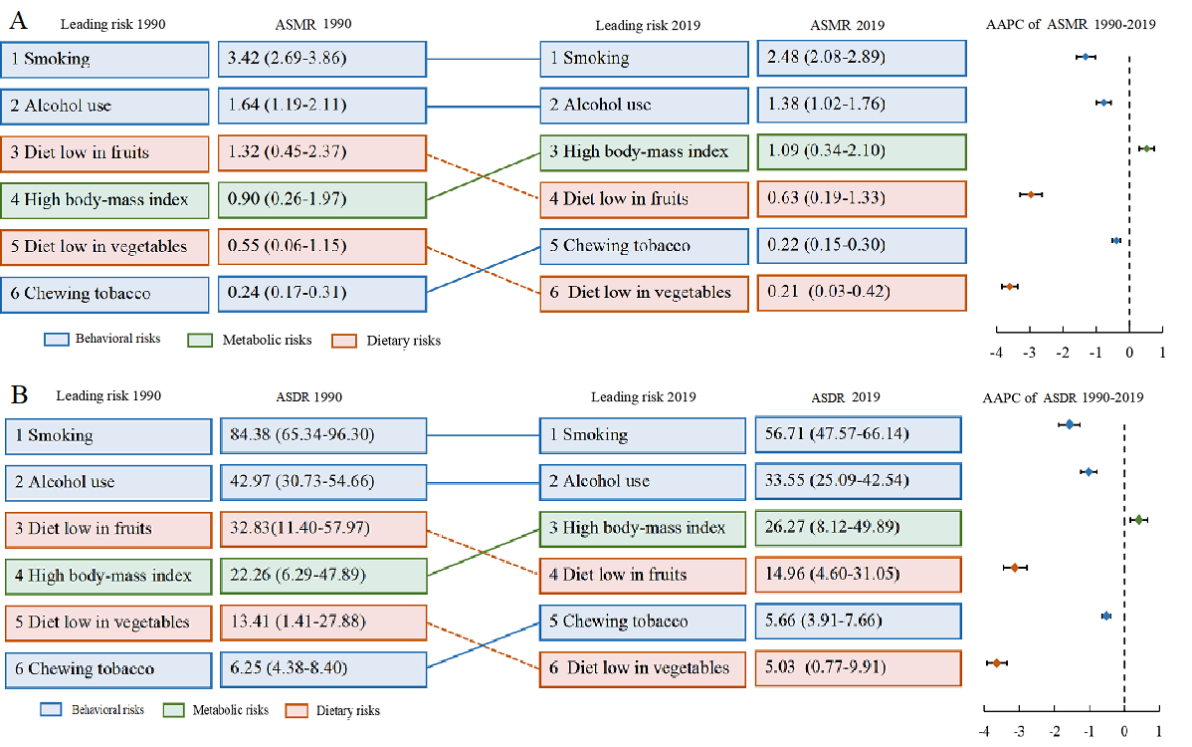
The changes in esophageal cancer (A) age-standardized mortality rate and (B) age-standardized disability-adjusted life year rate attributable to specific risk factors for both sexes combined in 1990-2019 globally. Dashed lines indicate decrease in rank. Solid lines indicate increase or no change in rank. Data in parentheses are 95% CIs. AAPC: average annual percentage change; ASDR: age-standardized disability-adjusted life year rate; ASMR: age-standardized mortality rate.

Behavioral risks include smoking, alcohol use, and chewing tobacco; metabolic risks include high BMI; and diet risks include diets low in fruits and vegetables. Figure 1 shows the changes in EC ASMR and ASDR by specific risk factors for both sexes combined from 1990 to 2019. Globally, smoking and alcohol use were the leading risk factors for EC burden, which declined significantly in the past 3 decades. The ASMRs attributable to diets low in fruits and vegetables had the most significant decrease, falling from the third and fifth positions in 1990 to the fourth and sixth positions in 2019, respectively. However, EC burden related to high BMI ranked third during this period. Notably, although the ranks of ASMR and ASDR attributable to chewing tobacco increased, the burden of EC attributed to chewing tobacco declined significantly (*P*<.05).

### Global Trends in EC Risk–Attributable Burden by Age Groups

**Figure 2:**
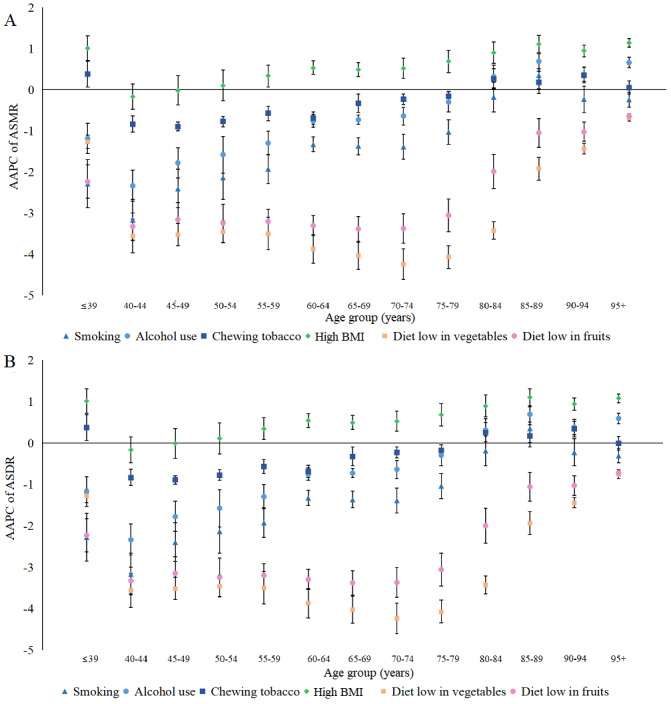
The average annual percentage change of (A) age-standardized mortality rate and (B) age-standardized disability-adjusted life year rate for both sexes combined by age groups in 1990-2019 globally. AAPC: average annual percentage change; ASDR: age-standardized disability-adjusted life year rate; ASMR: age-standardized mortality rate.

Figure 2 shows that smoking-related ASMRs and ASDRs declined in each age group except for the 85-89 years age group, with the highest decrease noted in the 40-44 years age group. Similarly, alcohol-related ASMR and ASDR declined in the 40-44 years age group, while it increased in the >80 years age group and peaked in the 85-89 years age group. The ASMRs and ASDRs attributable to diets low in fruits and vegetables declined in all age groups, but that attributable to high BMI increased in most age groups outside the subgroups within the age range of 40-54 years significantly (*P*<.05). The AAPCs of ASMR and ASDR for males and females are presented in Figures S1-S2 of Multimedia Appendix 1.

### Regional and SDI Trends in EC Risk–Attributable Burden

**Figure 3:**
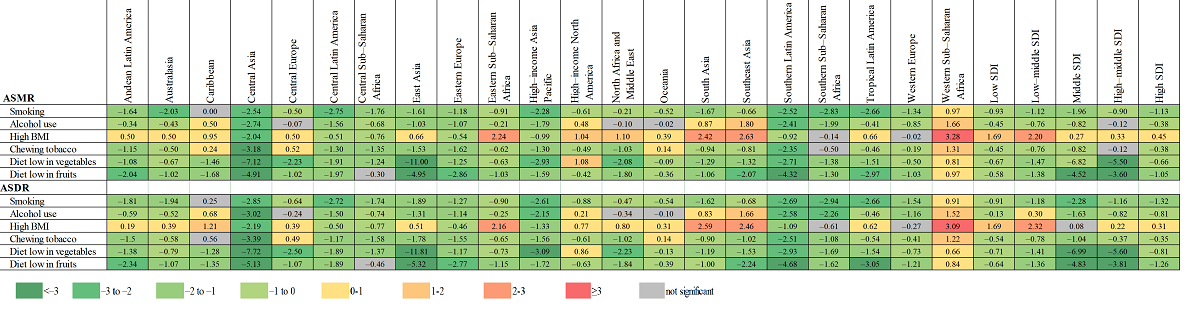
The average annual percentage change for esophageal cancer risk–attributable age-standardized mortality rate and age-standardized disability-adjusted life year rate in 21 global burden of disease regions classified by 5 sociodemographic index levels for both sexes in 1990-2019. Dark green indicates the highest decline in the average annual percentage change, dark red indicates the highest increase in the average annual percentage change, and grey indicates no significant average annual percentage change. AAPC: average annual percentage change; ASDR: age-standardized disability-adjusted life year rate; ASMR: age-standardized mortality rate; SDI: sociodemographic index.

As Figure 3 shows, there were significant downward trends in risk-attributable ASMRs and ASDRs in most regions with AAPC<0 compared to significant increases in high BMI–related ASMRs and ASDRs (*P*<.05). The EC burden attributable to each risk factor increased in Western Sub-Saharan Africa, while it declined significantly in Central Asia, Central Latin America, Eastern Europe, high-income Asia–Pacific, and Southern Latin America (*P*<.05). Among the risk factors, diets low in vegetables caused the most decline in the EC burden in East Asia and Central Asia, while high BMI caused the most increase in the ASMRs and ASDRs in Western Sub-Saharan Africa. Southern Sub-Saharan Africa and Central Asia showed the highest decline in smoking-related and alcohol use–related ASMRs and ASDRs, respectively. Figure 3 shows that the most dramatic decrease in EC burden was ascribed to low-vegetable diets in countries with middle SDI, followed by countries with high-middle SDI. In contrast, countries with low-middle SDI showed a sharp increase in EC burden, which is attributable to high BMI. In addition, Figures S1-S2 of Multimedia Appendix 1 show the changes in ASMRs and ASDRs attributable to risk factors among males and females in 21 GBD regions and 5 SDI levels. Table S1 of Multimedia Appendix 1 shows that the preponderance of EC burden for both sexes combined, in terms of the number of deaths and DALYs, were attributable to smoking, alcohol use, high BMI, and low-fruit diets in East Asia, while the highest number of deaths and DALYs attributable to chewing tobacco and low-vegetable diets occurred in South Asia in 2019 (Multimedia Appendix 1). Table S2 in Multimedia Appendix 1 shows distinct differences and risk gradients of ASMRs and ASDRs in 21 GBD regions and 5 SDI levels.

### National Trends in EC Risk–Attributable Burden

**Figure 4:**
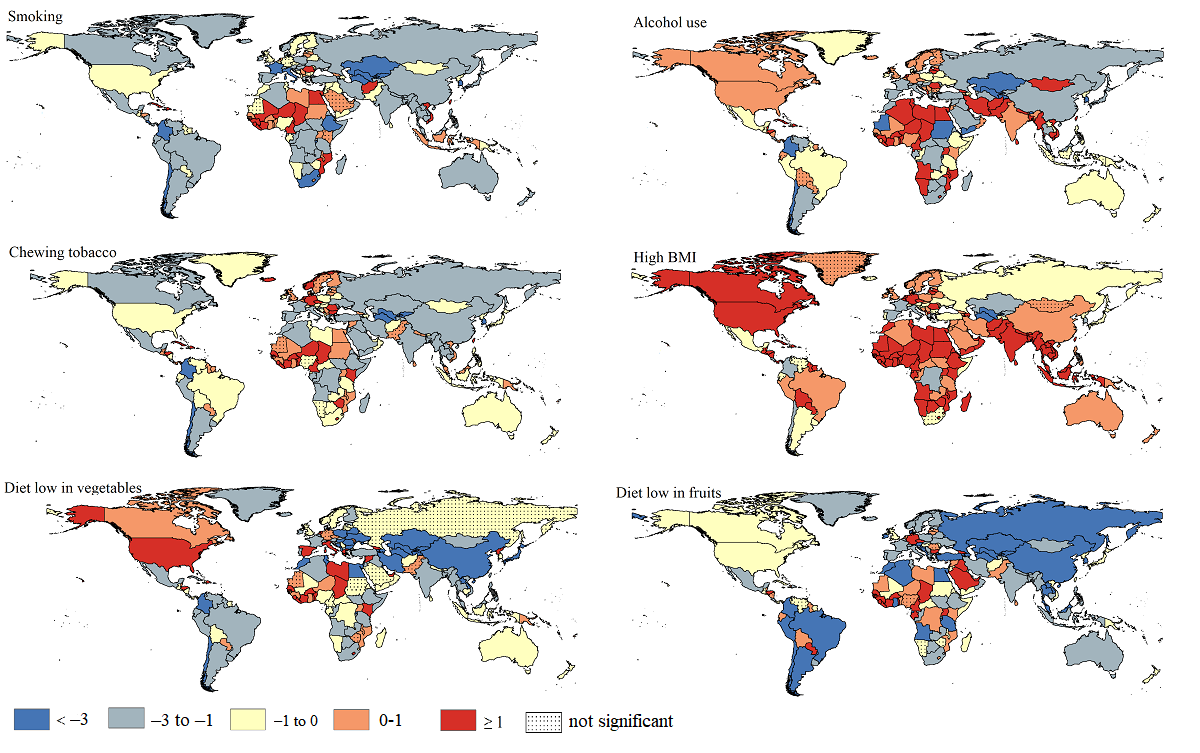
The average annual percentage change of esophageal cancer age-standardized mortality rate attributable to specific risk factors for both sexes combined in 204 countries and territories from 1990 to 2019.

Figure 4 shows the trends in risk-attributable ASMR in both sexes combined in 204 countries and territories from 1990 to 2019. There was a significant downward trend in ASMRs attributable to smoking, alcohol use, chewing tobacco, low-vegetable diets, and low-fruit diets in 136 (66.7%), 93 (45.6%), 118 (57.8%), 141 (69.1%), and 137 (67.2%) countries of all the 204 countries and territories, respectively. In contrast, a significantly upward trend in ASMR attributable to high BMI was observed in 139 (68.1%) countries and territories. Turkmenistan showed the highest decline in ASMRs attributable to smoking (AAPC=–5.21, 95% CI –5.99 to –4.42), high BMI (AAPC=–3.21, 95% CI –4.03 to –2.39), chewing tobacco (AAPC=–4.77, 95% CI –5.51 to –4.03), and low-vegetable diets (AAPC=–13.67, 95% CI –14.69 to –12.64), while the highest decline in ASMR attributable to alcohol use (AAPC=–8.02, 95% CI –10.94 to –5.00) and low-fruit diets (AAPC=–7.99, 95% CI –8.63 to –7.34) occurred in Sudan and Albania. São Tomé, Príncipe, and Northern Mariana had the highest increase in ASMR attributable to smoking (AAPC=3.40, 95% CI 3.17-3.62) and chewing tobacco (AAPC=3.58, 95% CI 3.08-4.08). Besides, Vietnam had the highest increase in ASMR attributable to alcohol use (AAPC=8.61, 95% CI 7.77-9.45) and high BMI (AAPC=5.94, 95% CI 5.55-6.34), but United Arab Emirates had the highest increase in ASMR attributable to low-vegetable diets (AAPC=11.01, 95% CI 9.47-12.56) and low-fruit diets (AAPC=5.10, 95% CI 4.50-5.72).

**Figure 5:**
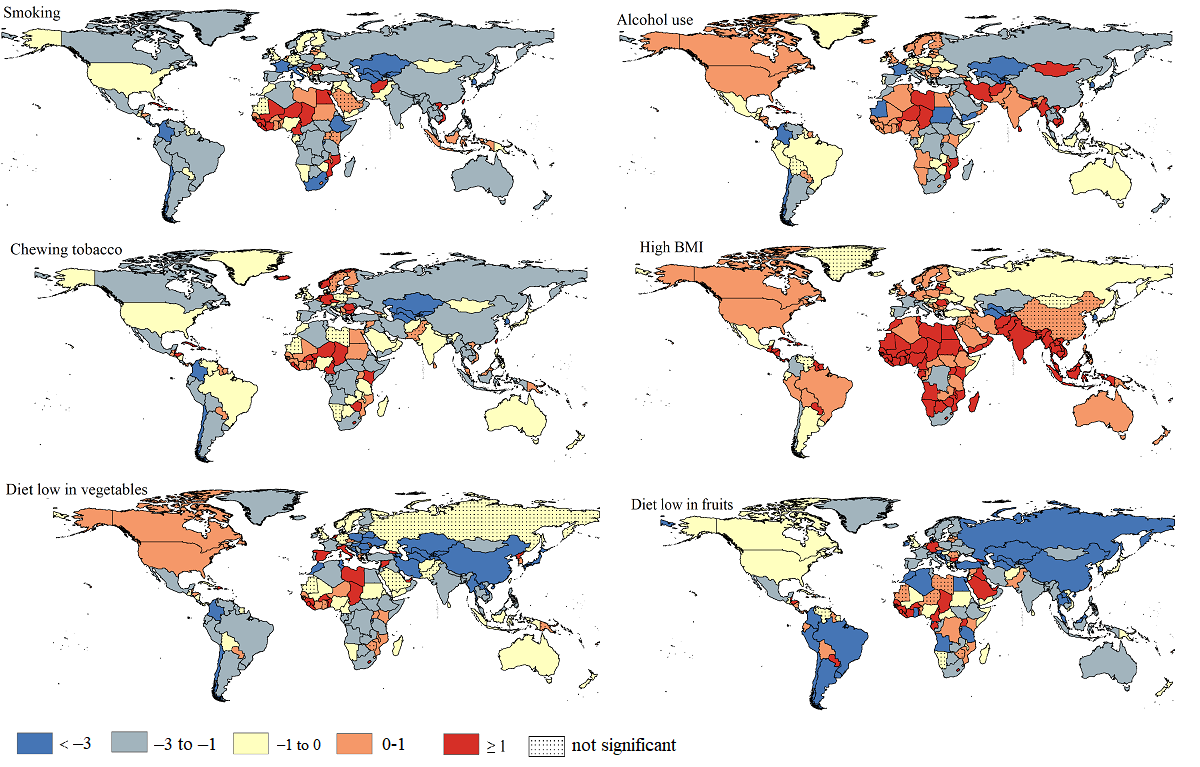
The average annual percentage change of esophageal cancer age-standardized disability-adjusted life year rate attributable to specific risk factors for both sexes combined in 204 countries and territories from 1990 to 2019.

As Figure 5 shows, from 1990 to 2019, significant decreases in ASDR attributable to smoking, alcohol use, chewing tobacco, low-vegetable diets, and low-fruit diets were found in 143 (70.1%), 96 (47.1%), 116 (56.9%), 145 (71.1%), and 143 (70.1%) countries of the 204 countries and territories, while 124 (60.7%) countries and territories experienced significant increases in ASDR attributable to high BMI. During this period, Turkmenistan had the highest decrease in ASDR attributable to smoking (AAPC=–5.31, 95% CI –6.09 to –4.53) and low-vegetable diets (AAPC=–14.49, 95% CI –15.66 to –13.31), while Uzbekistan had the highest decrease in ASDR attributable to chewing tobacco (AAPC=–4.97, 95% CI –5.48 to –4.26) and high BMI (AAPC=–3.37, 95% CI –3.89 to –2.84). The changes in the risk-attributable burden in other countries and territories are presented in Figure 5.

As Table S3 of Multimedia Appendix 1 shows, globally in 2019, China was the worst affected country with the highest number of EC deaths and DALYs attributable to smoking, alcohol use, high BMI, and low-fruit diets due to its large population and aging, accounting for about 50% of the total number of EC deaths and DALYs. China was followed by India, which had the highest number of EC-related deaths and DALYs attributable to chewing tobacco and low-vegetable diets.

## Discussion

### Principal Findings

This study uses the latest GBD 2019 data to analyze the spatiotemporal patterns in the burden of EC attributable to specific risk factors from 1990 to 2019, and our results show that the burden of EC varied with different risk factors at regional and national levels, among which EC disparities had different gradients of risk factors during this period. EC risk–attributable burden declined in most regions, countries, and territories, except for the increasing burden of EC attributed to high BMI. Our findings may be useful for the prognosis and treatment of EC by identifying the burden of modifiable risk factors worldwide for making clinical decisions about the risk attribution, stratification, and prevention.

Although the past 3 decades have witnessed a significant decrease in smoking-related EC burden, smoking still dominates among the risk-attributable factors. Moreover, the risk rank of EC burden attributable to chewing tobacco increased by 1 gradient from 1990 to 2019. The DALY of EC is estimated to be driven by tobacco smoking and chewing tobacco worldwide [10]. Compared with those in 1990, EC deaths and DALYs reduced by 55.9% and 42.1% in 2019, respectively, along with the corresponding ASMRs and ASDRs that annually declined by –1.18% and –1.41%, respectively, of which smoking resulted in 40.6% deaths [21]. Between 1990 and 2019, the trends in EC burden attributable to smoking largely varied across geographical locations, and there was a significant decrease mostly in regions outside the Caribbean and Western Sub-Saharan Africa. In 2003, the World Health Organization issued the “Framework Convention on Tobacco Control,” in which 168 countries joined this action and further worked out their own tobacco control strategies. EC deaths attributable to smoking continue to decrease with the reduction of smoking prevalence in high-income countries, paralleling with a decrease of 32.2% for males and 28.8% for females in age-standardized prevalence of smoking since 1990 [22,23]. Therefore, reducing smoking prevalence is an effective way to mitigate the EC burden.

Alcohol consumption still remains the second leading risk factor for EC burden from 1990 to 2019. The clear differences in EC burden over time attributable to alcohol use in GBD regions, as shown in our study, provides the most up-to-date information on the trends in EC risk–attributable burden. Approximately 22.6% of the deaths and 33.8% DALYs in EC are attributed to alcohol use worldwide [10,22]. Although alcohol-related EC deaths and DALYs increased by 13.1% and 8.3%, the change in the percentage of alcohol-related EC deaths and DALYs reduced by 14.4% and 17.1% from 1990 to 2017 [10,24]. The EC burden attributable to alcohol use declined in most regions (eg, Eastern Europe) concomitantly with the decrease in alcohol per capita consumption (from 11.2 L per capita to 9.8 L per capita) [25,26]. In contrast, a significant increase in EC burden related to alcohol use was observed in Southeast Asia, South Asia, Western Sub-Saharan Africa, and North America, largely because alcohol per capita consumption was high from 1990 to 2017 and is expected to remain high till 2030 [27-29]. For example, alcohol per capita consumption increased by 34% in Southeast Asia, ranging from 3.5 L per capita in 2010 to 4.7 L per capita in 2017 [27]. Thus, alcohol-related EC burden largely varies across geographical locations, and this variation should be carefully analyzed in the future.

The EC burden attributable to high BMI, in comparison with other risk factors, increased in most regions. This is consistent with the rising incidence of high BMI reported across the world, which has increased from 1975 to 2016 by nearly 12% for males, 24% for females, and 40% for both sexes combined [30]. Geographical differences in EC burden related to high BMI followed a geographical pattern of increasing obesity prevalence, with the largest absolute increase in high-income western countries, Central Asia, Middle East, and North Africa, and with the most relative increase in East and South-Eastern Asia [31]. However, previous studies have indicated that high BMI (or obesity) was a driver for EAC and not for ESCC [23]. This study also found that the geographical pattern of EC burden related to high BMI with AAPC>3 was generally consistent with that of EAC [32,33]. The slightly increased burden of EC attributable to high BMI, especially in typical ESCC high-risk regions such as China, suggest that EAC may show an upward trend due to increased prevalence of high BMI.

Consistent with that reported in previous studies [21,34], the burden of EC attributable to low-fruit and low-vegetable diets declined significantly, even if it remained stable or increased slightly in some regions. This aligns with the globally low consumption of fruits by 16.58% and vegetables by 25.60% from 1990 to 2017 [34,35]. The high dietary fibers of vegetables and fruits show a protective effect by generating inositol hexaphosphate to curb the growth rate of EC cells by reducing cellular proliferation and stimulating apoptosis [36]. Another plausible explanation is that high dietary fibers in vegetables and fruits decrease the levels of systemic inflammation factors such as tumor necrosis factor-α receptor-2 and interleukin-6, which may cause carcinogenesis [37]. The largest decrease in EC burden attributable to low intake of fruits and vegetables occurred in East Asia because of higher incomes and better health care. However, an upward trend in EC burden associated with low intake of vegetables occurred in North America and that associated with low intake of fruits occurred in Western Sub-Saharan Africa. In light of these variations, policy makers in each country should tailor related measures to promote well-balanced diets according to the local risk factors.

### Risk Exposure of EC burden Among Sexes and Age Groups

In terms of the spatiotemporal pattern in the risk exposure of EC burden, important differences in the EC burden between sexes and age groups in different locations should be mentioned. The time trend in EC burden attributed to smoking and chewing tobacco among sexes and age groups followed a similar pattern with a significant decrease, but the gradient showed a distinct difference among locations. This is possibly caused by the reduction of smoking prevalence in different regions, wherein males in 135 countries and females in 68 countries showed a prominent decrease in smoking prevalence [22]. Similarly, the greater decreases in EC burden due to alcohol use among females and in the 40-44 years age group indicate that interventions should target males and >45-year-old populations. Besides, the time trends of the EC burden attributable to high BMI for males and females did not follow the same pattern—with an increasing AAPC for males and a decreasing AAPC for females within the 40-64 years old subgroups. A previous study documented that high BMI resulted in ASDR increase of 12.7% for males and 26.8% for females, accounting for nearly 20% of the EC burden [38]. In the past decades, the prevalence of obesity increased by quadruple in men and more than double in women, and the time trend of ASMR attributable to high BMI showed a significant upward trend in 170 countries and territories, a downward trend only in 8 countries and territories, and a stable trend in 26 countries and territories [31,39]. The reason for this phenomenon is not completely understood, but the global prevalence of high BMI and obesity as well as deaths and DALY-related overweight are observed to be parallel with our findings across age groups, implying that sex differences in the prevalence of high BMI and obesity in the age groups are a potential risk factor [38,40]. Accordingly, the sex differences in EC burden related to high BMI should be addressed by ad hoc strategies, which are imperative for implementing regulations and policies targeting specific disorders.

### Public Health Implications

Our findings provide important evidence for tailored, country-specific policy development and inventions to address the geographical disparities in the EC burden, which can be used by governments at the national level to meet the challenges of the substantial burden of EC and develop strategies based on the priority of specific risk-attributable EC burden. Strategies such as behavioral and metabolic modifications (including smoking control, alcohol control policies and regulations, weight management tips) and access to abundant fruits and vegetables are imperative to face the challenges of EC. In light of the increase in EC burden related to high BMI in most countries, we recommend that public health policy makers and decision makers should prioritize their agenda by designing and implementing ad hoc effective policies to restrain the upward momentum of EAC. Moreover, encouraging balanced diets and reducing alcohol consumption and tobacco should constitute the essential component of EC prevention strategies. Educating the general public regarding risk factor modification is urgently needed in countries with heavy EC burden. Although it is not clear how diet influences EC development and progression, our study shows that EC burden attributable to low intake of fruits and vegetables has decreased significantly.

### Strengths and Limitations

This study provides a comprehensive spatiotemporal analysis of the EC burden attributable to specific risk factors, thereby becoming a potential reference for developing interventions that address similar health problems by identifying the most prominent risk factor and monitoring the effectiveness of the change of risk-attributable burden of EC over time. Further studies are required to conduct early interventions targeting behavioral and metabolic risk factors of individuals based on our findings. Moreover, the accelerating growth of EC burden related to high BMI alerts us to prevent and curb this issue as early as possible. We hope these limitations can be solved in the future. The 2 distinct histological subtypes of EC, that is, ESCC and EAC, have completely different risk factors, trends, and geographical patterns, but data on ESCC and EAC are unavailable in GBD. Thus, we did not analyze the changes of EC burden attributable to risk factors by subtype. Second, some countries had no EC data because of the lack of registries, or even if they had data in several registries that did not cover the whole country and territory, the estimates were obtained by modelling. The EC data in countries without cancer registries were modeled, but the model did not solve the geographical approximates of EC, because the distribution of EC showed clear clusters. Third, we did not analyze the spatiotemporal changes of EC burden attributable to other risk factors such as consumption of hot meals, eating red or processed meat, physical activity, and infectious agents, because GBD has no data on these variables. This may overestimate the burden of EC attributable to the included risk factors in this study. Fourth, this study did not capture the potential synergy effects between risk factors in which some combinations might be multiplicative. For some risk factors such as behavior and diet, the joint effect of smoking and alcohol use on the burden of EC is very important for framing public policy. Further, more detailed work is needed to strengthen the evidence base for understanding the mediation effects of sex, age, and geographical locations on the risk-attributable burden of EC.

### Conclusion

This study analyzes the spatiotemporal patterns in the risk-attributable burden of EC over the past 3 decades, suggesting that smoking and alcohol use remained the dominant risk factors and drive the burden of EC compared with other risk-outcome pairs. The EC burden caused by smoking, alcohol use, chewing tobacco, low-fruit diets, and low-vegetable diets has decreased over the last 3 decades, but the EC burden attributable to high BMI has increased significantly. In addition, changes in the burden of EC due to modifiable risk factors varied widely across regions, with the largest decrease in EC burden in East Asia, especially in China. Although the exact reasons for the marked geographical differences are still unclear, a large number of epidemiological studies are ongoing to yield valuable findings. Our findings targeted specific risk factors of EC burden at different geographical scales to elucidate the key modifiable risk factors for the prevention and control of EC in line with achieving the target 3.4 of the Sustainable Development Goals. Further, understanding the epidemiological trend and risk factor stratification of EC are the key pathways for public health and clinical decisions regarding risk stratification, screening, and prevention. These results might be conducive for identifying the leading modifiable risk factors for countries that might not have previous local research on EC burden and risk factor exposure.

## Appendix

Multimedia Appendix 1 Supplementary data.

## Abbreviations

AAPC: average annual percentage change
ASDR: age-standardized disability-adjusted life year rate
ASMR: age-standardized mortality rate
DALY: disability-adjusted life year
EAC: esophageal adenocarcinoma
EC: esophageal cancer
ESCC: esophageal squamous cell carcinoma
GBD: global burden of disease
SDI: sociodemographic index
UI: uncertainty interval

## Data Availability

Publicly available data sets were analyzed in this study. The processed data sets and statistical methods in the analysis are available from the corresponding author upon reasonable request.
