# Supplementary material for: Spatiotemporal Patterns of Esophageal Cancer Burden Attributable to Behavioral, Metabolic, and Dietary Risk Factors From 1990 to 2019: Longitudinal Observational Study
Source: JMIR Public Health Surveill. 2023 Oct 6;9:e46051. doi: 10.2196/46051 (PMC10589835; doi:10.2196/46051)
Supplement: Multimedia Appendix 1 [file publichealth_v9i1e46051_app1.pdf]

Multimedia Appendix1

The spatiotemporal patterns of esophageal cancer burden attributable to behavioral, metabolic, and dietary risk factors from 1990 to 2019: a population-based study

Table of content

Figure S1 The AAPCs of ASMR (a) and ASDR (b) for males at age groups in 1990-2019, globally.

Figure S2 The AAPCs of ASMR (a) and ASDR (b) for females at age groups in 1990-2019, globally.

Figure S3. The AAPC for EC risk attributable ASMR and ASDR for males (a) and females (b) in 21 GBD regions and 5 SDI levels, 1990-2019.

Figure S4. The AAPC of EC ASMR attributable to specific risk factors for males at 204 countries and territories from 1990 to 2019.

Figure S5. The AAPC of EC ASMR attributable to specific risk factors for females at 204 countries and territories from 1990 to 2019.

Figure S6. The AAPC of EC ASDR attributable to specific risk factors for males at 204 countries and territories from 1990 to 2019.

Figure S7. The AAPC of EC ASDR attributable to specific risk factors for females at 204 countries and territories from 1990 to 2019.

Table S1. The burden of EC attributable to behavioral, metabolic and diet factors in 21 GBD regions in 1990 and 2019.

Table S2. The burden of EC attributable to behavioral, metabolic and diet factors in 5 SDI levels in 1990 and 2019

Table S3. The burden of EC attributable to bahavioral, metobolic, and diet risk factors for both sexes combined in 204 countries and territories in 2019

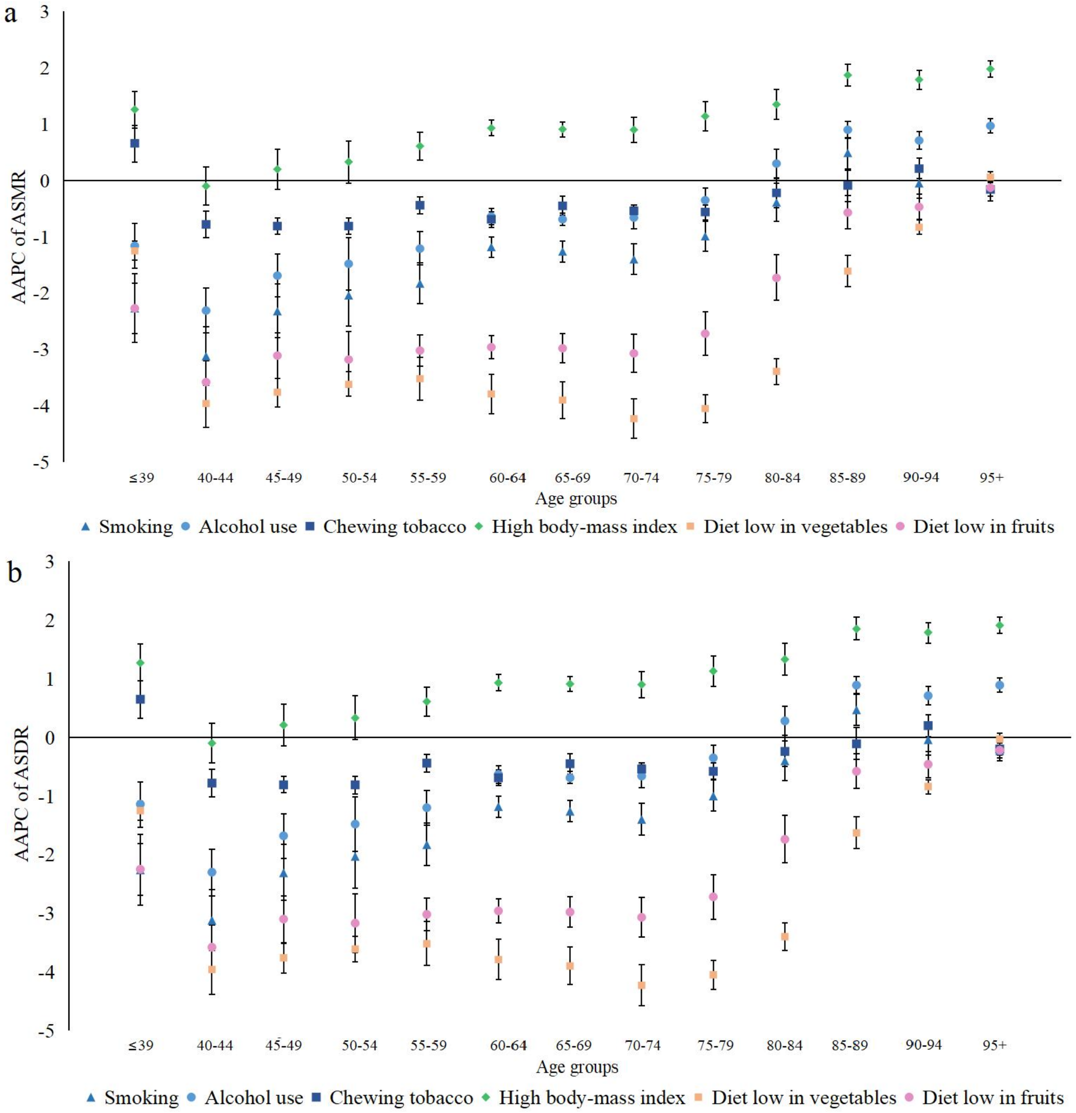

Figure S1. The AAPCs of ASMR (a) and ASDR (b) for males in age groups in 1990-2019, globally. (AAPC: average annual percentage change; ASMR: age-standardized mortality rate; ASDR:age-standardized DALY rate.)

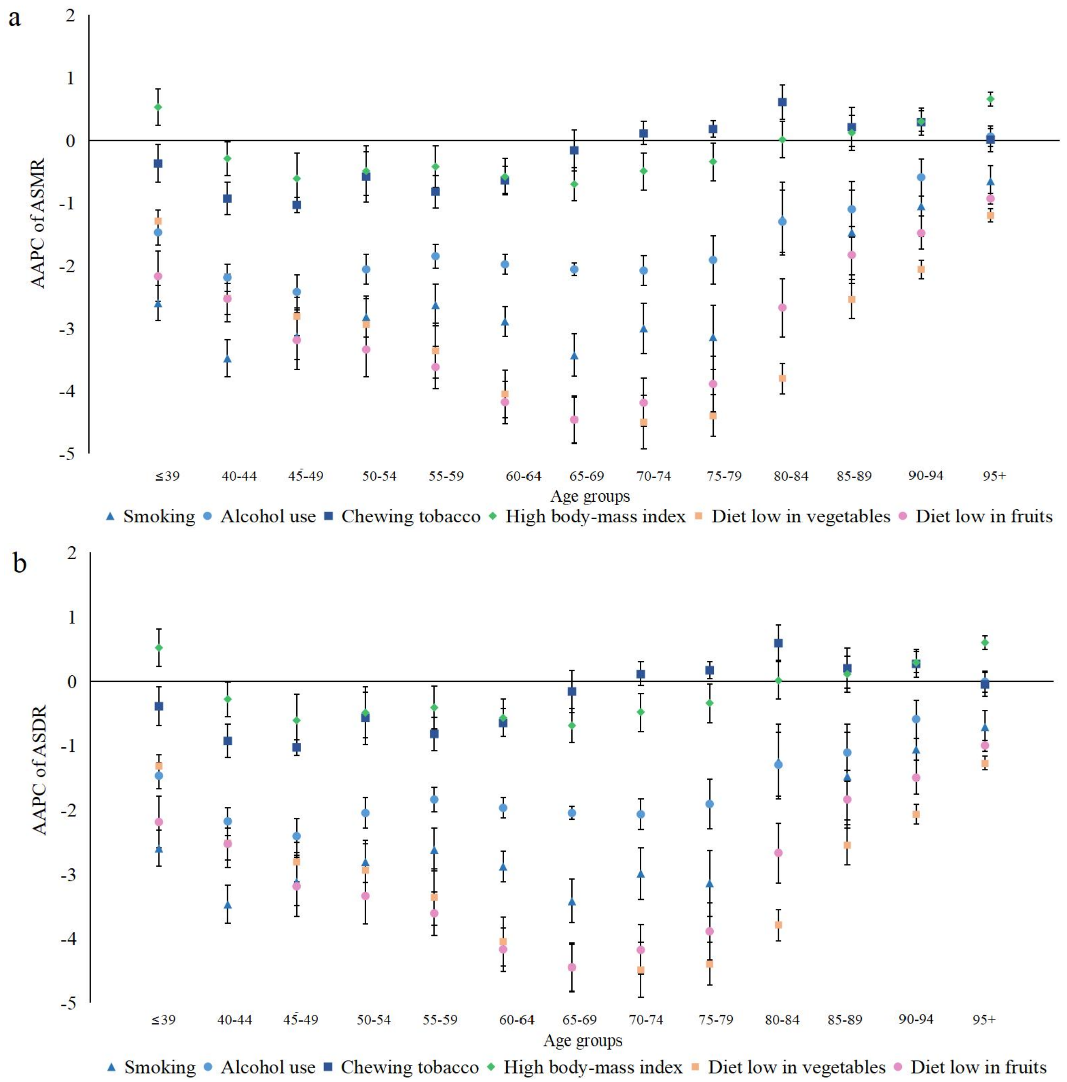

Figure S2. The AAPCs of ASMR (a) and ASDR (b) for females in age groups in 1990-2019, globally. (AAPC: average annual percentage change; ASMR: age-standardized mortality rate; ASDR:age-standardized DALY rate.)

a

ASMR	Andean Latin America	Australasia	Caribbean	Central Asia	Central Europe	Central Latin America	Central Sub-Saharan Africa	East Asia	Eastern Europe	Eastern Sub-Saharan Africa	High-income Asia Pacific	High-income North America	North Africa and Middle East	Oceania	South Asia	Southeast Asia	Southern Latin America	Southern Sub-Saharan Africa	Tropical Latin America	Western Europe	Western Sub-Saharan Africa	Low SDI	Low-middle SDI	Middle SDI	High-middle SDI	High SDI
Smoking	-1.55	-2.01	0.26	-2.71	-0.59	-2.36	-1.60	-1.36	-1.45	-0.84	-2.46	-0.61	-0.13	-0.45	-1.42	-0.40	-2.67	-2.66	-2.44	-1.63	1.31	-0.80	-0.88	-1.71	-0.89	-1.28
Alcohol use	-0.27	-0.35	0.67	-2.65	-0.11	-1.29	-0.60	-0.82	-1.18	-0.05	-1.91	0.44	0.12	-0.04	1.09	1.94	-2.41	-1.83	-0.32	-1.09	2.04	0.05	0.55	-1.10	-0.58	-0.69
High BMI	0.74	0.71	1.47	-1.69	0.67	0.06	-0.79	1.56	-0.26	2.50	-0.98	1.22	1.81	0.68	2.93	3.05	-0.93	0.08	0.96	-0.16	4.06	1.93	2.69	1.07	0.72	0.42
Chewing tobacco	-0.96	-0.67	0.68	-3.32	0.64	-0.92	-1.20	-1.12	-1.51	-0.68	-1.53	-0.74	-0.81	0.22	-0.73	-0.31	-2.50	-1.71	-0.19	-0.34	1.84	-0.32	-0.60	-0.80	-0.17	-0.69
Diet low in vegetables	-1.80	-0.80	-1.51	-4.90	-0.91	-1.46	-0.45	-4.14	-2.74	-1.01	-1.67	-0.35	-1.61	-0.25	-0.78	-1.72	-4.45	-1.48	-2.77	-1.13	1.47	-0.53	-1.34	-6.45	-5.45	-0.71
Diet low ine fruits	-0.85	-0.51	-1.17	-6.93	-2.14	-1.46	-1.32	-10.13	-1.21	-0.59	-3.01	1.15	-1.83	0.06	-1.01	-1.01	-2.75	-1.55	-1.29	-0.62	1.25	-0.38	-1.21	-3.89	-3.29	-1.09
**ASDR**																										
Smoking	-1.73	-1.84	0.53	-2.95	-0.74	-2.34	-1.70	-1.67	-1.46	-0.80	-2.80	-0.86	-0.36	-0.45	-1.38	-0.46	-2.86	-2.72	-2.49	-1.80	1.35	-0.76	-0.95	-2.04	-1.14	-1.45
Alcohol use	-0.54	-0.38	0.86	-2.94	-0.28	-1.25	-0.75	-1.12	-1.22	-0.06	-2.31	0.19	-0.07	-0.12	1.02	1.76	-2.63	-2.08	-0.39	-1.37	2.02	0.06	0.46	-1.43	-0.80	-0.94
High BMI	0.42	0.65	1.71	-1.94	0.46	0.05	-0.83	1.37	-0.26	2.46	-1.40	0.93	1.57	0.56	3.11	2.92	-1.16	-0.38	0.89	-0.44	3.99	1.95	2.78	0.85	0.56	0.31
Chewing tobacco	-1.36	-0.64	0.95	-3.48	0.55	-0.81	-1.29	-1.42	-1.50	-0.62	-1.81	-0.78	-0.82	0.23	-0.72	-0.42	-2.71	-2.03	-0.30	-0.55	1.89	-0.41	-0.62	-1.02	-0.38	-0.57
Diet low in vegetables	-2.17	-0.83	-1.20	-5.17	-1.04	-1.41	-0.60	-4.56	-2.71	-1.01	-1.88	-0.58	-1.63	-0.28	-0.74	-1.82	-4.88	-1.71	-2.87	-1.36	1.49	-0.49	-1.30	-6.70	-5.58	-0.91
Diet low in fruits	-1.20	-0.59	-1.00	-7.54	-2.47	-1.46	-1.46	-11.07	-1.19	-0.59	-3.27	0.92	-2.00	0.01	-0.95	-1.18	-3.03	-1.77	-1.35	-0.89	1.25	-0.38	-1.21	-4.72	-3.51	-1.34

<-3 -3 to -2 -2 to -1 -1 to 0 0 to 1 1 to 2 2 to 3 ≥ 3 No significance

b

ASMR	Andean Latin America	Australasia	Caribbean	Central Asia	Central Europe	Central Latin America	Central Sub-Saharan Africa	East Asia	Eastern Europe	Eastern Sub-Saharan Africa	High-income Asia Pacific	High-income North America	North Africa and Middle East	Oceania	South Asia	Southeast Asia	Southern Latin America	Southern Sub-Saharan Africa	Tropical Latin America	Western Europe	Western Sub-Saharan Africa	Low SDI	Low-middle SDI	Middle SDI	High-middle SDI	High SDI
Smoking	-2.05	-2.48	-1.45	-2.77	-0.43	-4.14	-1.65	-4.00	-0.20	-0.83	-2.67	-1.38	-1.17	-1.10	-1.86	-2.36	-1.95	-3.10	-3.28	-0.98	-0.53	-1.04	-1.77	-4.18	-2.34	-1.48
Alcohol use	-0.53	-1.32	-0.88	-3.54	-0.64	-2.66	-0.51	-2.90	-1.52	-0.43	-2.20	-0.13	-1.33	-0.28	0.97	0.73	-2.34	-1.97	-0.44	-0.51	0.69	-0.60	0.18	-2.93	-2.18	-0.69
High BMI	0.08	-0.21	-0.44	-2.40	-0.33	-1.47	-0.55	-1.25	-1.85	1.98	-1.68	0.01	0.21	-0.25	1.86	1.97	-0.85	-0.28	-0.02	0.02	2.16	1.43	1.60	-1.09	-0.92	-0.17
Chewing tobacco	-1.45	-1.10	-1.12	-2.77	-0.55	-2.39	-1.55	-3.54	-2.52	-0.50	-1.12	-1.12	-1.90	-0.39	-1.15	-1.25	-1.99	-0.36	-0.99	-0.43	0.51	-0.57	-0.90	-0.74	-0.22	-0.91
Diet low in vegetables	-2.41	-1.72	-2.03	-4.98	-1.62	-2.80	-0.01	-6.59	-3.85	-1.06	-2.11	-1.12	-2.04	-0.66	-1.37	-2.64	-3.94	-0.80	-3.31	-1.12	0.15	-0.82	-1.54	-7.36	-5.87	-1.02
Diet low ine fruits	-1.50	-1.36	-2.17	-7.40	-2.66	-2.69	-1.01	-12.26	-2.05	-0.67	-3.20	0.21	-2.43	-0.48	-1.58	-1.82	-2.53	-0.87	-1.96	-0.61	0.08	-0.79	-1.52	-5.56	-4.64	-1.45
**ASDR**																										
Smoking	-2.22	-2.52	-1.48	-2.91	-0.44	-4.16	-1.75	-4.52	0.28	-0.97	-2.27	-1.64	-1.44	-1.19	-2.03	-2.73	-2.02	-3.50	-3.16	-0.88	-0.76	-1.17	-1.90	-4.53	-2.65	-1.46
Alcohol use	-0.68	-1.45	-0.81	-3.58	-0.58	-2.60	-0.62	-3.38	-1.21	-0.65	-1.96	-0.33	-1.91	-0.32	1.00	0.50	-2.32	-2.50	-0.44	-0.50	0.29	-0.77	0.22	-3.28	-2.38	-0.73
High BMI	-0.24	-0.44	-0.30	-2.45	-0.27	-1.54	-0.64	-1.71	-1.56	1.84	-1.53	-0.21	-0.19	-0.34	2.00	1.68	-0.88	-0.80	-0.16	0.08	1.71	1.38	1.71	-1.37	-1.09	-0.25
Chewing tobacco	-1.75	-1.15	-0.91	-2.75	-0.47	-2.42	-1.78	-4.15	-2.11	-0.68	-0.59	-1.11	-1.97	-0.38	-1.07	-1.68	-1.99	-0.86	-1.12	-0.35	0.09	-0.70	-0.95	-0.91	-0.53	-0.77
Diet low in vegetables	-2.62	-1.85	-1.69	-5.12	-1.46	-2.74	-0.24	-7.31	-3.46	-1.32	-1.61	-1.25	-2.08	-0.75	-1.28	-3.03	-4.14	-1.25	-3.45	-1.01	-0.26	-0.98	-1.50	-7.48	-5.92	-0.97
Diet low in fruits	-1.70	-1.58	-2.04	-8.02	-2.81	-2.72	-1.22	-13.45	-1.47	-0.90	-2.97	0.09	-2.55	-0.56	-1.44	-2.21	-2.59	-1.31	-2.01	-0.52	-0.35	-0.95	-1.50	-6.01	-4.98	-1.41

<-3 -3 to -2 -2 to -1 -1 to 0 0 to 1 1 to 2 2 to 3 ≥ 3 No significance

Figure S3. The AAPC for EC risk attributable ASMR and ASDR for males (a) and females (b) in 21 GBD regions and 5 SDI levels, 1990-2019.

AAPC: average annual percentage change; ASMR: age-standardized mortality rate; ASDR:age-standardized DALY rate. AAPC from the highest decline (dark green) to the highest rise (dark red), where no significant AAPC was marked as grey).

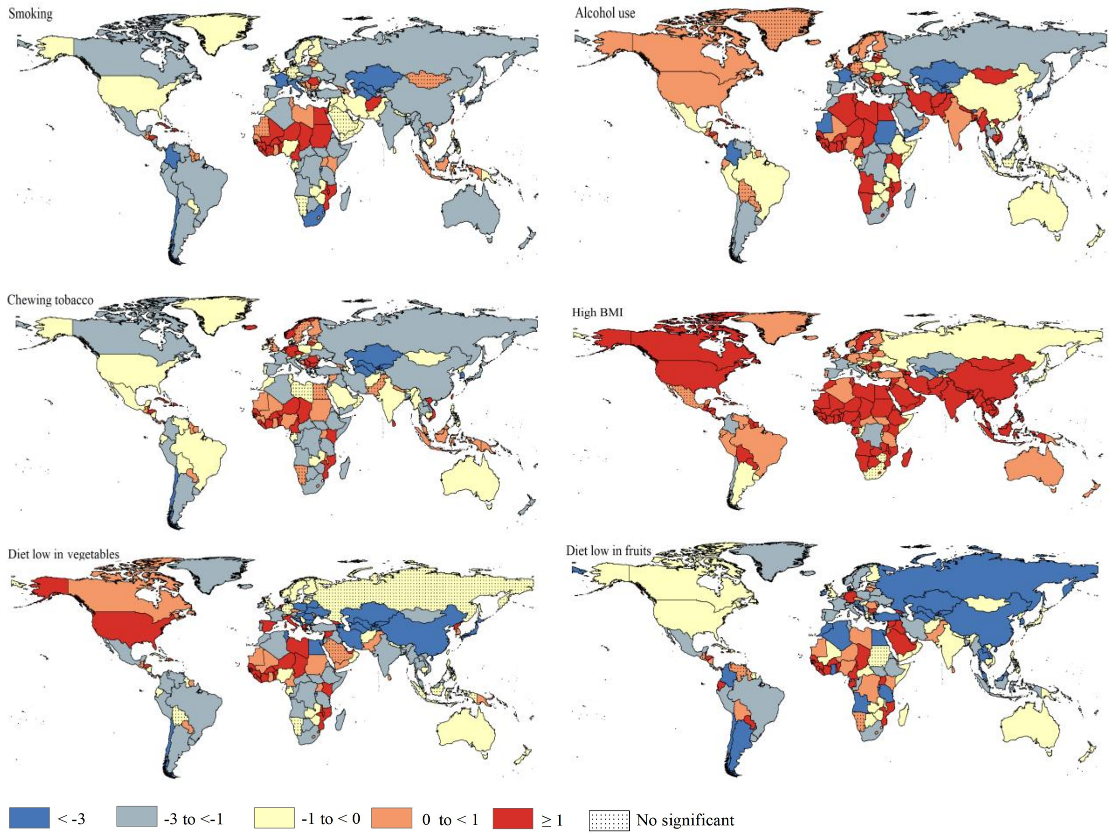

Figure S4. The AAPC of EC ASMR attributable to specific risk factors for males at 204 countries and territories from 1990 to 2019.

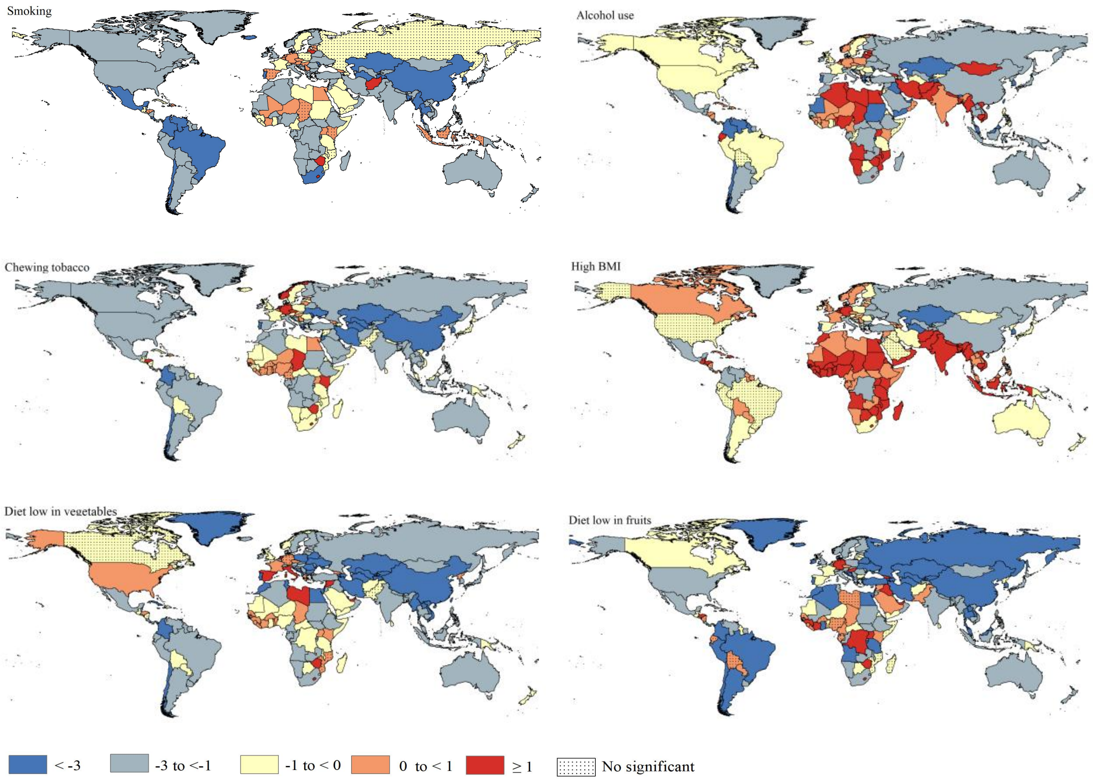

Figure S5. The AAPC of EC ASMR attributable to specific risk factors for females at 204 countries and territories from 1990 to 2019.

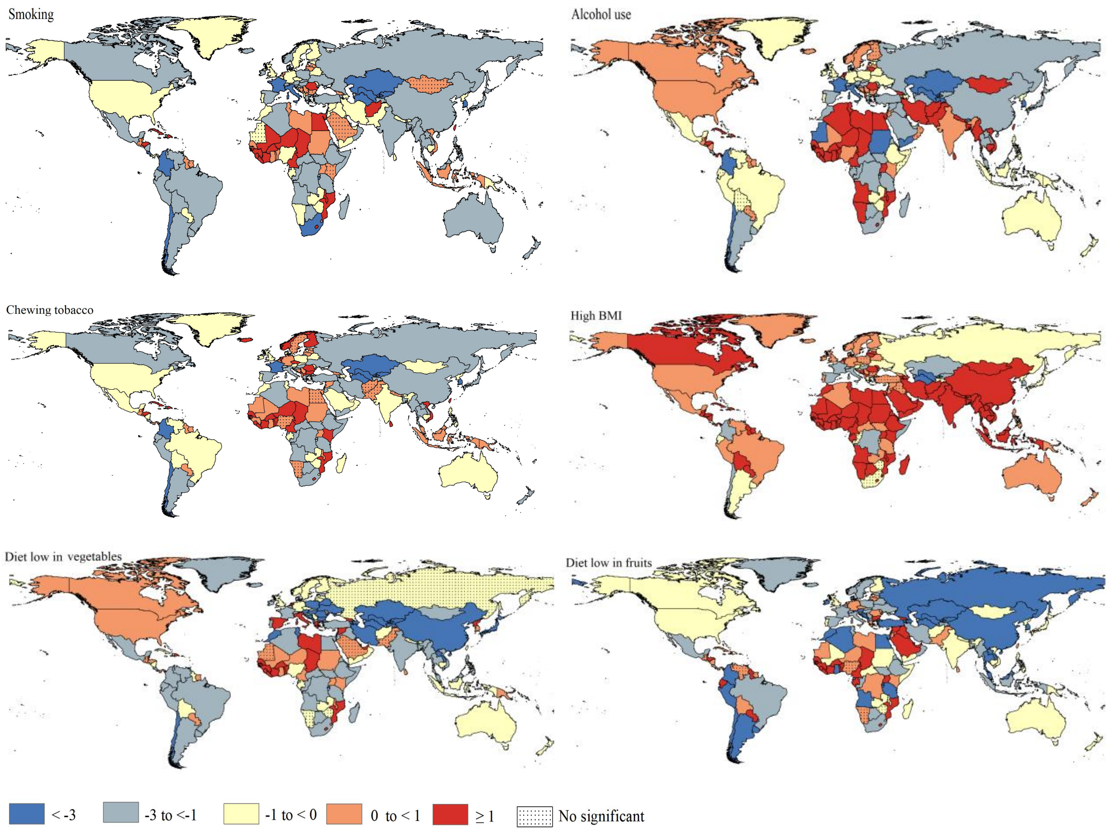

Figure S6. The AAPC of EC ASDR attributable to specific risk factors for males at 204 countries and territories from 1990 to 2019.

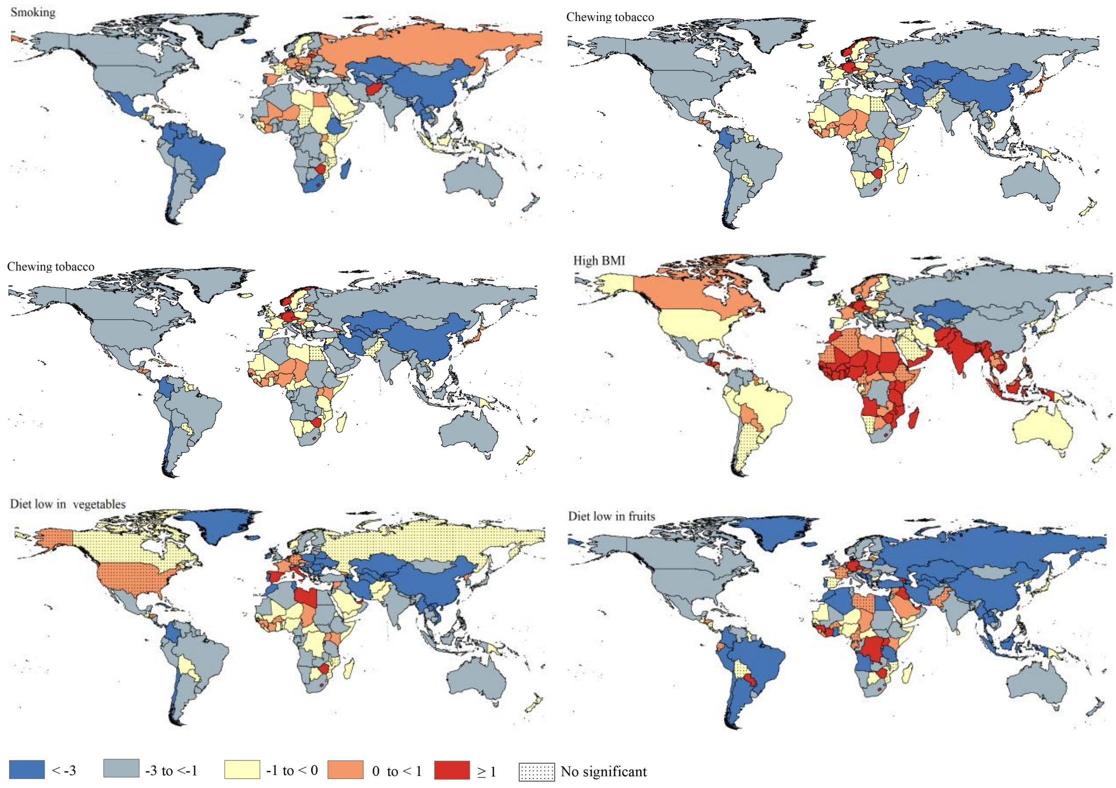

Figure S7. The AAPC of EC ASDR attributable to specific risk factors for females at 204 countries and territories from 1990 to 2019

Table S1. The burden of EC attributable to behavioral, metabolic and diet factors in 21 GBD regions and 5 SDI levels in 1990 and 2019

Region	1990				2019				AAPC(1990-2019)	
	Deaths (95%UI)	ASMR (95%UI)	DALYs (95%UI)	ASDR (95%UI)	Deaths (95%UI)	ASMR (95%UI)	DALYs (95%UI)	ASDR (95%UI)	ASMR (95%CI)	ASDR (95%CI)
**Smoking**										
Andean Latin America	87 (67,106)	0.45 (0.34, 0.54)	2084 (1585,2559)	10.08 (7.7, 12.37)	147 (106, 200)	0.27 (0.19,0.37)	3242 (2301, 4514)	5.78 (4.12, 8.01)	-1.64 (-1.75, -1.52)	-1.81 (-1.95, -1.67)
Australasia	425 (372,475)	1.79 (1.57, 1.99)	9699 (8584,10783)	41.57 (36.91, 46.18)	498 (412, 595)	1.02 (0.85, 1.22)	10935 (9174, 12848)	24.1 (20.2, 28.17)	-2.03 (-2.09, -1.96)	-1.94 (-1.99, -1.89)
Caribbean	362 (316,407)	1.42 (1.23, 1.59)	8665 (7542,9830)	33.1 (28.85, 37.61)	678 (545, 816)	1.3 (1.05, 1.57)	16920 (13576, 20620)	32.29 (25.91, 39.24)	0.00 (-0.15, 0.15)	0.25 (0.1, 0.4)
Central Asia	2056 (1856,2259)	4.37 (3.93, 4.81)	55713 (50036,61273)	112.79 (101.31, 124.14)	1554 (1316, 1949)	2.16 (1.83, 2.68)	41375 (34680, 52740)	51.73 (43.78, 65.53)	-2.54 (-2.8, -2.27)	-2.85 (-3.12, -2.59)
Central Europe	2162 (1964,2341)	1.45 (1.32, 1.57)	59817 (54502,64690)	40.2 (36.67, 43.48)	2667 (2222, 3149)	1.31 (1.08, 1.54)	68154 (56259, 81204)	35.35 (29.05, 42.1)	-0.5 (-0.6, -0.4)	-0.64 (-0.77, -0.52)
Central Latin America	663 (569,749)	0.84 (0.72, 0.95)	16185 (13768,18444)	18.99 (16.25, 21.48)	924 (714, 1162)	0.40 (0.31, 0.5)	21465 (16335, 27465)	8.97 (6.86, 11.45)	-2.75 (-2.86, -2.64)	-2.72 (-2.83, -2.6)
Central Sub-Saharan Africa	515 (232,717)	2.22 (1.01, 3.14)	14667 (6475,20514)	57.27 (25.65, 79.97)	795 (424, 1158)	1.47 (0.79, 2.14)	23069 (12079, 33795)	38.16 (20.37, 55.8)	-1.76 (-1.99, -1.53)	-1.74 (-1.96, -1.52)
East Asia	76974 (49276,92685)	9.02 (5.88, 10.79)	1987661 (1251627,2422130)	213.99 (136.15, 259.34)	125696 (95840, 155773)	6.06 (4.63, 7.49)	2875590 (2203977, 3577906)	132.28 (101.86, 164)	-1.61 (-2.03, -1.19)	-1.89 (-2.34, -1.45)

Region	1990				2019				AAPC(1990-2019)	
	Deaths (95%UI)	ASMR (95%UI)	DALYs (95%UI)	ASDR (95%UI)	Deaths (95%UI)	ASMR (95%UI)	DALYs (95%UI)	ASDR (95%UI)	ASMR (95%CI)	ASDR (95%CI)
Eastern Europe	5350	1.85	152114	52.80	5062	1.47	137934	41.37	-1.18	-1.27
	(4774,5920)	(1.65, 2.05)	(135537,169351)	(47.07, 58.67)	(4226, 5970)	(1.23, 1.74)	(115225, 163254)	(34.59, 48.99)	(-1.4, -0.96)	(-1.52, -1.03)
Eastern Sub-Saharan	1901	2.62	51930	64.66	3296	2.08	90952	51.75	-0.91	-0.9
Africa	(1258,2554)	(1.75, 3.5)	(33607,70309)	(42.34, 87.2)	(2239, 4734)	(1.44, 2.95)	(60797, 132636)	(34.93, 74.65)	(-1.06, -0.77)	(-1.06, -0.74)
High-income Asia	5664	2.8	138158	66.28	6948	1.54	135322	33.76	-2.28	-2.61
Pacific	(5188,6103)	(2.56, 3.01)	(125921,149111)	(60.52, 71.46)	(6000, 7931)	(1.34, 1.75)	(119012, 154437)	(29.63, 38.49)	(-2.45, -2.10)	(-2.82, -2.4)
High-income North	6842	1.98	161003	48.82	10815	1.71	235489	39.01	-0.61	-0.88
America	(6038,7600)	(1.76, 2.19)	(145113,175907)	(44.13, 53.11)	(9303, 12369)	(1.48, 1.94)	(206908, 263962)	(34.49, 43.52)	(-0.74, -0.47)	(-1.01, -0.76)
North Africa and	1355	0.81	36925	19.93	3132	0.76	81057	17.51	-0.21	-0.47
Middle East	(982,1635)	(0.6, 0.97)	(25601,45034)	(14.23, 24.2)	(2393, 3696)	(0.59, 0.89)	(59499, 96665)	(13.17, 20.67)	(-0.26, -0.16)	(-0.53, -0.41)
Oceania	24	0.80	692	20.6	48	0.69	1409	17.56	-0.52	-0.54
	(17,35)	(0.56, 1.17)	(474,993)	(14.18, 29.65)	(33, 68)	(0.48, 0.97)	(950, 1998)	(12.15, 24.83)	(-0.62, -0.42)	(-0.64, -0.43)
South Asia	8102	1.50	224647	36.46	13271	0.96	347985	23.53	-1.67	-1.62
	(6627,10110)	(1.25, 1.86)	(182213,284520)	(29.76, 45.49)	(10243, 18923)	(0.75, 1.38)	(267726, 492693)	(18.19, 33.26)	(-1.77, -1.56)	(-1.71, -1.52)
Southeast Asia	2816	1.13	76706	27.92	5811	0.96	154630	23.4	-0.66	-0.68
	(2308,3327)	(0.94, 1.34)	(61266,90931)	(22.6, 33.17)	(4689, 7132)	(0.78, 1.17)	(122821, 188896)	(18.66, 28.61)	(-0.72, -0.59)	(-0.73, -0.62)
Southern Latin	1391	2.99	34580	73.56	1307	1.56	30065	36.79	-2.52	-2.69
America	(1222,1557)	(2.63, 3.36)	(30686,38397)	(65.33, 81.72)	(1110, 1526)	(1.33, 1.83)	(25668, 34482)	(31.39, 42.24)	(-2.68, -2.36)	(-2.85, -2.53)
Southern	1530	5.59	43469	146.76	1780	3.15	49030	80.71	-2.83	-2.94
Sub-Saharan Africa	(1117,1974)	(4.06, 7.21)	(31566,56220)	(106.51,	(1450, 2215)	(2.58, 3.86)	(39400, 62244)	(65.22,	(-3.46, -2.2)	(-3.56, -2.31)

Region	1990				2019				AAPC(1990-2019)	
	Deaths (95%UI)	ASMR (95%UI)	DALYs (95%UI)	ASDR (95%UI)	Deaths (95%UI)	ASMR (95%UI)	DALYs (95%UI)	ASDR (95%UI)	ASMR (95%CI)	ASDR (95%CI)
				189.45)				101.44)		
Tropical Latin America	3164 (2835,3477)	3.52 (3.16, 3.89)	85408 (76718,93832)	87.52 (78.6, 96.23)	3862 (3294, 4461)	1.58 (1.35, 1.82)	98300 (83475, 113734)	39.20 (33.35, 45.38)	-2.66 (-2.84, -2.48)	-2.66 (-2.87, -2.45)
Western Europe	12978 (11827,14192)	2.29 (2.1, 2.5)	305909 (279090,331955)	57.03 (51.93, 61.74)	14181 (12484, 16165)	1.61 (1.42, 1.81)	299291 (262808, 336263)	37.78 (33.12, 42.22)	-1.34 (-1.42, -1.26)	-1.54 (-1.63, -1.46)
Western Sub-Saharan Africa	332 (251,425)	0.37 (0.28, 0.47)	9285 (6904,12032)	9.74 (7.31, 12.57)	855 (559, 1123)	0.45 (0.3, 0.58)	24312 (15877, 32419)	11.62 (7.63, 15.28)	0.97 (0.79, 1.14)	0.91 (0.73, 1.09)
**Alcohol use**										
Andean Latin America	64 (42, 86)	0.31 (0.2,0.42)	1829 (1242, 2432)	8.15 (5.45,10.9)	143 (93, 211)	0.26 (0.17,0.38)	3698 (2441, 5360)	6.41 (4.22,9.29)	-0.34 (-0.56, -0.12)	-0.59 (-0.8, -0.37)
Australasia	372 (291, 445)	1.58 (1.23,1.9)	8529 (6740, 10200)	36.78 (29.07,43.86)	709 (543, 881)	1.43 (1.1,1.77)	14780 (11503, 18231)	32.06 (25.1,39.3)	-0.43 (-0.5, -0.35)	-0.52 (-0.59, -0.45)
Caribbean	195 (146, 249)	0.75 (0.56,0.97)	5266 (3932, 6704)	19.8 (14.77,25.17)	416 (299, 546)	0.8 (0.57,1.05)	11490 (8272, 15134)	21.96 (15.8,28.95)	0.5 (0.24, 0.76)	0.68 (0.44, 0.93)
Central Asia	1088 (774, 1414)	2.25 (1.58,2.94)	31760 (23034, 40830)	62.77 (45.26,80.85)	875 (630, 1166)	1.15 (0.82,1.54)	25203 (18070, 33478)	30.01 (21.64,39.91)	-2.74 (-3.03, -2.45)	-3.02 (-3.32, -2.73)
Central Europe	1497 (1182, 1790)	1.01 (0.8,1.21)	42574 (33767, 50662)	28.75 (22.75,34.16)	2076 (1580, 2578)	1.03 (0.79,1.28)	54237 (41428, 67410)	28.61 (21.83,35.73)	-0.07 (-0.18, 0.03)	-0.24 (-0.37, -0.1)
Central Latin America	364 (269, 457)	0.44 (0.32,0.55)	10127 (7608, 12619)	11.11 (8.3,13.89)	713 (502, 949)	0.3 (0.21,0.4)	18851 (13450, 25043)	7.71 (5.5,10.25)	-1.56 (-1.75, -1.36)	-1.5 (-1.69, -1.31)

Region	1990				2019				AAPC(1990-2019)	
	Deaths (95%UI)	ASMR (95%UI)	DALYs (95%UI)	ASDR (95%UI)	Deaths (95%UI)	ASMR (95%UI)	DALYs (95%UI)	ASDR (95%UI)	ASMR (95%CI)	ASDR (95%CI)
Central Sub-Saharan Africa	385 (165, 584)	1.67 (0.72,2.53)	11314 (4803, 17347)	43.43 (18.65,66.12)	738 (373, 1142)	1.38 (0.7,2.16)	21814 (11000, 33587)	35.42 (17.93,54.45)	-0.68 (-1.12, -0.23)	-0.74 (-1.15, -0.32)
East Asia	34441 (21297, 47245)	3.87 (2.39,5.28)	951890(584990, 1297661)	99.46 (60.98,135.73)	63286 (44055, 85935)	3 (2.09,4.05)	1554078 (1084262,2106781)	70.86 (49.51,95.9)	-1.03 (-1.36, -0.69)	-1.31 (-1.68, -0.95)
Eastern Europe	3636 (2816, 4407)	1.26 (0.98,1.53)	105752 (82283, 127855)	36.82 (28.65,44.5)	3615 (2711, 4552)	1.06 (0.8,1.34)	100673 (75714, 126841)	30.58 (23.01,38.58)	-1.07 (-1.29, -0.84)	-1.14 (-1.39, -0.9)
Eastern Sub-Saharan Africa	1454 (960, 1947)	1.9 (1.26,2.54)	42767 (28116, 57570)	50.56 (33.29,68.02)	3026 (2019, 4329)	1.81 (1.21,2.58)	89792 (59802, 129809)	47.98 (32.1,68.75)	-0.21 (-0.24, -0.18)	-0.25 (-0.3, -0.21)
High-income Asia Pacific	3107 (2359, 3826)	1.52 (1.16,1.88)	80034 (60943, 97796)	38.24 (29.06,46.75)	4460 (3223, 5686)	1.02 (0.74,1.28)	90423 (66730, 113998)	23.39 (17.43,29.49)	-1.79 (-1.93, -1.65)	-2.15 (-2.32, -1.99)
High-income North America	3278 (2478, 4071)	0.97 (0.74,1.2)	82790 (63137, 102306)	25.64 (19.68,31.61)	6844 (5116, 8447)	1.11 (0.83,1.36)	158522 (119277, 195673)	27.13 (20.47,33.38)	0.48 (0.42, 0.54)	0.21 (0.16, 0.26)
North Africa and Middle East	122 (72, 173)	0.06 (0.04,0.09)	3939 (2240, 5635)	1.93 (1.12,2.77)	296 (182, 421)	0.06 (0.04,0.09)	9267 (5601, 13282)	1.8 (1.1,2.58)	-0.1 (-0.27, 0.06)	-0.34 (-0.47, -0.2)
Oceania	6 (4, 10)	0.2 (0.11,0.32)	202 (116, 320)	5.59 (3.18,8.88)	14 (8, 23)	0.19 (0.1,0.31)	456 (246, 729)	5.29 (2.79,8.45)	-0.02 (-0.26, 0.22)	-0.1 (-0.33, 0.13)
South Asia	1648 (1064, 2369)	0.27 (0.17,0.39)	51393 (33595, 73750)	7.63 (4.93,10.97)	5069 (3322, 7623)	0.35 (0.23,0.52)	149610 (97824, 222038)	9.7 (6.34,14.46)	0.87 (0.74, 1.01)	0.83 (0.7, 0.96)
Southeast Asia	682 (428, 940)	0.26 (0.16,0.35)	20675 (13056, 28407)	7.06 (4.45,9.74)	2677 (1851, 3530)	0.42 (0.3,0.56)	77022 (52620, 101730)	11.28 (7.73,14.85)	1.80 (1.67, 1.92)	1.66 (1.53, 1.78)

Region	1990				2019				AAPC(1990-2019)	
	Deaths (95%UI)	ASMR (95%UI)	DALYs (95%UI)	ASDR (95%UI)	Deaths (95%UI)	ASMR (95%UI)	DALYs (95%UI)	ASDR (95%UI)	ASMR (95%CI)	ASDR (95%CI)
Southern Latin America	1259 (984, 1507)	2.77 (2.16,3.32)	30057 (23675, 36011)	64.38 (50.75,77.07)	1286 (967, 1595)	1.53 (1.15,1.89)	27982 (21121, 34430)	34.17 (25.75,42.03)	-2.41 (-2.58, -2.25)	-2.58 (-2.74, -2.41)
Southern Sub-Saharan Africa	935 (627, 1264)	3.30 (2.2,4.48)	28432 (19167, 38053)	92.85 (62.16,125.08)	1355 (985, 1780)	2.38 (1.73,3.1)	38573 (28197, 51399)	62.40 (45.29,82.57)	-1.99 (-2.63, -1.35)	-2.26 (-2.91, -1.61)
Tropical Latin America	1232 (909, 1573)	1.28 (0.95,1.65)	37471 (27990, 47359)	36.49 (27.2,46.44)	2887 (2132, 3657)	1.16 (0.85,1.47)	82656 (62252, 103972)	32.55 (24.5,40.95)	-0.41 (-0.6, -0.22)	-0.46 (-0.66, -0.27)
Western Europe	9613 (7650, 11522)	1.73 (1.38,2.07)	237043 (190761, 282761)	45.01 (36.27,53.56)	12178 (9516, 14679)	1.40 (1.09,1.67)	262498 (206270, 314886)	33.66 (26.52,40.28)	-0.85 (-0.95, -0.75)	-1.16 (-1.26, -1.05)
Western Sub-Saharan Africa	292 (200, 392)	0.33 (0.22,0.44)	8269 (5690, 11102)	8.57 (5.91,11.5)	933 (632, 1270)	0.50 (0.33,0.68)	26565 (17916, 36407)	12.58 (8.5,17.14)	1.66 (1.52, 1.81)	1.52 (1.38, 1.66)
**Chewing tobacco**										
Andean Latin America	1 (1, 2)	0.01 (0, 0.01)	38 (24, 56)	0.17 (0.11, 0.25)	3 (2,4)	0.01 (0,0.01)	62 (37,94)	0.11 (0.07,0.16)	-1.15 (-1.24, -1.06)	-1.5 (-1.6, -1.4)
Australasia	8 (5, 12)	0.03 (0.02, 0.05)	177 (102, 270)	0.76 (0.44, 1.16)	16 (9,25)	0.03 (0.02,0.05)	307 (175,476)	0.65 (0.37,1.02)	-0.5 (-0.67, -0.33)	-0.58 (-0.76, -0.4)
Caribbean	4 (3, 6)	0.02 (0.01, 0.02)	116 (71, 169)	0.44 (0.27, 0.64)	9 (5,13)	0.02 (0.01,0.03)	247 (147,368)	0.47 (0.28,0.71)	0.24 (-0.01, 0.49)	0.56 (0.31, 0.82)
Central Asia	118 (69, 174)	0.24 (0.14, 0.36)	3479 (2026, 5162)	6.85 (4.02, 10.04)	93 (53,143)	0.12 (0.07,0.18)	2759 (1582,4276)	3.21 (1.86,4.95)	-3.18 (-3.51, -2.85)	-3.39 (-3.74, -3.04)
Central Europe	17	0.01	478	0.33	28	0.01	721	0.39	0.52	0.49

Region	1990				2019				AAPC(1990-2019)	
	Deaths (95%UI)	ASMR (95%UI)	DALYs (95%UI)	ASDR (95%UI)	Deaths (95%UI)	ASMR (95%UI)	DALYs (95%UI)	ASDR (95%UI)	ASMR (95%CI)	ASDR (95%CI)
	(10, 25)	(0.01, 0.02)	(280, 700)	(0.19, 0.48)	(17,	(0.01,0.02)	(425,1085)	(0.23,0.58)	(0.42, 0.62)	(0.4, 0.58)
Central Latin	15	0.02	380	0.44	32	0.01	779	0.32	-1.3	-1.17
America	(9, 22)	(0.01, 0.03)	(233, 551)	(0.27, 0.63)	(19,49)	(0.01,0.02)	(450,1159)	(0.19,0.48)	(-1.42, -1.18)	(-1.3, -1.05)
Central Sub-Saharan	51	0.24	1453	5.80	83	0.17	2283	3.97	-1.35	-1.58
Africa	(22, 84)	(0.1, 0.39)	(592, 2390)	(2.44, 9.4)	(40,145)	(0.08,0.3)	(1088,3942)	(1.93,6.86)	(-1.53, -1.16)	(-1.77, -1.39)
East Asia	1115	0.12	32631	3.36	1787	0.08	47099	2.15	-1.53	-1.78
	(592, 1775)	(0.07, 0.19)	(17089, 52160)	(1.78, 5.4)	(925,2889)	(0.04,0.13)	(24120,77769)	(1.11,3.51)	(-1.95, -1.11)	(-2.23, -1.33)
Eastern Europe	38	0.01	1010	0.36	33	0.01	867	0.26	-1.62	-1.55
	(23, 55)	(0.01, 0.02)	(601, 1492)	(0.21, 0.51)	(19,49)	(0.01,0.01)	(491,1326)	(0.15,0.4)	(-1.87, -1.36)	(-1.82, -1.27)
Eastern Sub-Saharan	290	0.39	8509	10.17	529	0.32	15510	8.4	-0.62	-0.65
Africa	(178, 414)	(0.24, 0.54)	(5165, 12188)	(6.21, 14.5)	(334,770)	(0.21,0.47)	(9729,22849)	(5.26,12.29)	(-0.72, -0.51)	(-0.78, -0.53)
High-income Asia	100	0.05	2405	1.16	160	0.04	3073	0.77	-1.30	-1.56
Pacific	(55, 157)	(0.03, 0.08)	(1338, 3733)	(0.65, 1.81)	(87,253)	(0.02,0.06)	(1715,4773)	(0.43,1.19)	(-1.43, -1.17)	(-1.67, -1.46)
High-income North	589	0.17	13644	4.16	975	0.16	21599	3.67	-0.49	-0.61
America	(335, 884)	(0.1, 0.25)	(7762, 20467)	(2.34, 6.23)	(534,1495)	(0.09,0.24)	(11975,32941)	(2.05,5.62)	(-0.7, -0.27)	(-0.77, -0.44)
North Africa and	119	0.07	3326	1.78	244	0.06	6947	1.41	-1.03	-1.02
Middle East	(62, 184)	(0.04, 0.11)	(1645, 5258)	(0.9, 2.78)	(123,374)	(0.03,0.09)	(3279,10865)	(0.69,2.2)	(-1.19, -0.87)	(-1.15, -0.89)
Oceania	2	0.04	52	1.34	4	0.05	131	1.39	0.14	0.14
	(1, 2)	(0.02, 0.07)	(29, 83)	(0.74, 2.12)	(2,6)	(0.03,0.07)	(73,217)	(0.79,2.25)	(0.11, 0.16)	(0.11, 0.18)
South Asia	6194	1.16	175247	28.15	12847	0.94	340799	22.94	-0.94	-0.9

Region	1990				2019				AAPC(1990-2019)	
	Deaths (95%UI)	ASMR (95%UI)	DALYs (95%UI)	ASDR (95%UI)	Deaths (95%UI)	ASMR (95%UI)	DALYs (95%UI)	ASDR (95%UI)	ASMR (95%CI)	ASDR (95%CI)
	(4418, 8301)	(0.84, 1.56)	(124367, 236586)	(20.11, 37.83)	(9096,18061)	(0.66,1.32)	(239988,475407)	(16.16,32.04)	(-1.09, -0.8)	(-1.02, -0.78)
Southeast Asia	486<br>(330, 662)	0.21<br>(0.14, 0.29)	12098<br>(8068, 16743)	4.64<br>(3.12, 6.38)	925<br>(622,1255)	0.17<br>(0.11,0.23)	21236<br>(14145,28973)	3.45<br>(2.3,4.68)	-0.81<br>(-0.94, -0.68)	-1.02<br>(-1.16, -0.89)
Southern Latin America	9<br>(6, 14)	0.02<br>(0.01, 0.03)	209<br>(131, 306)	0.45<br>(0.28, 0.66)	10<br>(6,15)	0.01<br>(0.01,0.02)	202<br>(125,299)	0.24<br>(0.15,0.36)	-2.35<br>(-2.52, -2.17)	-2.51<br>(-2.68, -2.34)
Southern Sub-Saharan Africa	63<br>(37, 98)	0.25<br>(0.14, 0.4)	1567<br>(943, 2346)	5.47<br>(3.28, 8.25)	115<br>(67,181)	0.24<br>(0.13,0.39)	2477<br>(1523,3748)	4.51<br>(2.73,6.94)	-0.50<br>(-1, 0)	-1.08<br>(-1.59, -0.56)
Tropical Latin America	55<br>(34, 82)	0.07<br>(0.04, 0.1)	1235<br>(742, 1855)	1.37<br>(0.84, 2.06)	135<br>(79,207)	0.06<br>(0.03,0.09)	2774<br>(1640,4214)	1.14<br>(0.68,1.73)	-0.46<br>(-0.53, -0.39)	-0.54<br>(-0.6, -0.48)
Western Europe	73<br>(45, 107)	0.01<br>(0.01, 0.02)	1696<br>(1006, 2534)	0.32<br>(0.19, 0.47)	112<br>(68,166)	0.01<br>(0.01,0.02)	2305<br>(1367,3387)	0.29<br>(0.17,0.42)	-0.19<br>(-0.23, -0.14)	-0.41<br>(-0.46, -0.36)
Western Sub-Saharan Africa	50<br>(31, 71)	0.06<br>(0.04, 0.08)	1324<br>(822, 1900)	1.43<br>(0.89, 2.04)	138<br>(82,206)	0.08<br>(0.05,0.12)	3683<br>(2154,5493)	1.84<br>(1.09,2.74)	1.31<br>(1.17, 1.45)	1.22<br>(1.09, 1.35)
**High BMI**										
Andean Latin America	78<br>(25, 153)	0.39<br>(0.12, 0.78)	2029<br>(653, 3879)	9.44<br>(3, 18.07)	245<br>(82, 449)	0.45<br>(0.15, 0.82)	5600<br>(1897, 10225)	9.87<br>(3.32, 17.96)	0.5<br>(0.41, 0.59)	0.19<br>(0.09, 0.28)
Australasia	249<br>(83, 449)	1.06<br>(0.35, 1.92)	5690<br>(1850, 10182)	24.63<br>(8, 43.92)	631<br>(224, 1090)	1.27<br>(0.45, 2.17)	13021<br>(4529, 22378)	28.16<br>(9.73, 48)	0.5<br>(0.38, 0.62)	0.39<br>(0.28, 0.49)
Caribbean	196<br>(66, 370)	0.77<br>(0.25, 1.45)	4853<br>(1611, 9069)	18.42<br>(6.12, 34.44)	484<br>(150, 877)	0.93<br>(0.29, 1.69)	12409<br>(3818, 22637)	23.73<br>(7.3, 43.34)	0.95<br>(0.69, 1.21)	1.21<br>(0.96, 1.46)

Region	1990				2019				AAPC(1990-2019)	
	Deaths (95%UI)	ASMR (95%UI)	DALYs (95%UI)	ASDR (95%UI)	Deaths (95%UI)	ASMR (95%UI)	DALYs (95%UI)	ASDR (95%UI)	ASMR (95%CI)	ASDR (95%CI)
Central Asia	1504 (488, 2709)	3.26 (1.03, 5.87)	39632 (13103, 70992)	81.14 (26.5, 145.63)	1424 (489, 2480)	1.99 (0.68, 3.45)	38393 (12709, 67193)	47.83 (16.43, 83.25)	-2.04 (-2.38, -1.71)	-2.19 (-2.52, -1.85)
Central Europe	1213 (407, 2126)	0.82 (0.28, 1.45)	33036 (10597, 57504)	22.23 (7.13, 38.72)	2022 (690, 3456)	0.99 (0.34, 1.69)	50676 (16622, 87404)	26.35 (8.34, 45.56)	0.5 (0.39, 0.62)	0.39 (0.24, 0.53)
Central Latin America	398 (130, 752)	0.5 (0.16, 0.95)	10108 (3361, 19012)	11.54 (3.78, 21.82)	1069 (364, 1949)	0.46 (0.16, 0.84)	25414 (8466, 46217)	10.55 (3.53, 19.18)	-0.51 (-0.6, -0.42)	-0.5 (-0.59, -0.41)
Central Sub-Saharan Africa	286 (71, 654)	1.25 (0.3, 2.88)	8290 (2050, 19019)	32.17 (7.91, 73.85)	639 (171, 1349)	1.20 (0.32, 2.61)	18649 (4948, 38470)	30.62 (8.21, 64.41)	-0.76 (-1.17, -0.34)	-0.77 (-1.18, -0.36)
East Asia	11987 (1984, 32689)	1.41 (0.23, 3.88)	312386 (50580, 839095)	33.51 (5.5, 91.09)	36940 (9621, 81495)	1.77 (0.46, 3.9)	881413 (224528, 1929647)	40.4 (10.38, 88.17)	0.66 (0.17, 1.17)	0.51 (-0.03, 1.04)
Eastern Europe	2955 (978, 5409)	1.04 (0.36, 1.89)	78159 (25458, 143685)	27.21 (8.88, 50.12)	3381 (1141, 5942)	0.98 (0.33, 1.73)	88533 (28263, 159064)	26.52 (8.41, 47.25)	-0.54 (-0.81, -0.28)	-0.46 (-0.74, -0.17)
Eastern Sub-Saharan Africa	691 (149, 1668)	0.91 (0.19, 2.23)	20354 (4561, 48846)	24.06 (5.27, 57.93)	2673 (803, 5484)	1.63 (0.47, 3.34)	77843 (23372, 157367)	42.18 (12.69, 85.8)	2.24 (2.14, 2.35)	2.16 (2.06, 2.25)
High-income Asia Pacific	1026 (211, 2455)	0.51 (0.11, 1.21)	25779 (4992, 61948)	12.35 (2.49, 29.76)	1874 (377, 4222)	0.42 (0.08, 0.95)	37045 (7142, 82943)	9.47 (1.89, 21.13)	-0.99 (-1.15, -0.83)	-1.33 (-1.51, -1.14)
High-income North America	3222 (1050, 5808)	0.95 (0.31, 1.71)	79194 (26082, 142707)	24.42 (8.02, 43.78)	8199 (2775, 13507)	1.31 (0.45, 2.16)	186230 (62500, 304731)	31.54 (10.62, 51.67)	1.04 (0.83, 1.25)	0.77 (0.58, 0.97)
North Africa and Middle East	880 (260, 1644)	0.51 (0.15, 0.96)	25027 (7516, 47286)	13.16 (3.91, 24.7)	2962 (1030, 5041)	0.71 (0.25, 1.21)	79894 (27248, 136935)	16.82 (5.78, 28.62)	1.1 (1.03, 1.17)	0.8 (0.72, 0.88)

Region	1990				2019				AAPC(1990-2019)	
	Deaths (95%UI)	ASMR (95%UI)	DALYs (95%UI)	ASDR (95%UI)	Deaths (95%UI)	ASMR (95%UI)	DALYs (95%UI)	ASDR (95%UI)	ASMR (95%CI)	ASDR (95%CI)
Oceania	10 (3, 21)	0.34 (0.1, 0.73)	310 (93, 653)	8.90 (2.6, 18.62)	28 (8, 56)	0.4 (0.12, 0.83)	848 (253, 1684)	10.2 (3.01, 20.44)	0.39 (0.24, 0.54)	0.31 (0.13, 0.49)
South Asia	1539 (361, 3790)	0.28 (0.06, 0.68)	45229 (10677, 109505)	7.05 (1.66, 17.26)	8116 (2529, 16015)	0.57 (0.18, 1.13)	230148 (73617, 449398)	15.13 (4.81, 29.65)	2.42 (2.19, 2.66)	2.59 (2.4, 2.79)
Southeast Asia	457 (104, 1117)	0.17 (0.04, 0.43)	13642 (3190, 32852)	4.7 (1.09, 11.42)	2309 (728, 4564)	0.37 (0.12, 0.75)	64696 (20148, 126435)	9.61 (2.98, 18.79)	2.63 (2.56, 2.7)	2.46 (2.38, 2.54)
Southern Latin America	662 (206, 1286)	1.46 (0.45, 2.85)	15420 (4576, 30335)	33.07 (9.72, 65.02)	1071 (366, 1906)	1.27 (0.44, 2.25)	22526 (7763, 40271)	27.42 (9.41, 49.06)	-0.92 (-1.18, -0.65)	-1.09 (-1.34, -0.84)
Southern Sub-Saharan Africa	833 (267, 1565)	3.01 (0.94, 5.65)	24375 (7785, 45483)	80.79 (26.09, 152.28)	1776 (633, 3023)	3.27 (1.15, 5.51)	46780 (17149, 79226)	78.16 (28.57, 132.81)	-0.14 (-0.72, 0.44)	-0.61 (-1.2, -0.01)
Tropical Latin America	1203 (357, 2268)	1.31 (0.41, 2.49)	33877 (10047, 63913)	34.1 (10.13, 64.18)	3785 (1277, 6516)	1.55 (0.52, 2.66)	100018 (33039, 172467)	39.75 (13.2, 68.52)	0.66 (0.52, 0.81)	0.62 (0.45, 0.78)
Western Europe	5690 (1788, 10435)	1.01 (0.31, 1.86)	134831 (41681, 250504)	25.28 (7.77, 47.21)	9279 (3001, 16333)	1.05 (0.34, 1.84)	194261 (62758, 342685)	24.6 (7.81, 43.61)	-0.02 (-0.14, 0.11)	-0.27 (-0.4, -0.13)
Western Sub-Saharan Africa	202 (56, 443)	0.23 (0.06, 0.5)	5710 (1613, 12222)	5.95 (1.67, 12.86)	997 (316, 1894)	0.54 (0.17, 1.02)	27917 (8772, 53122)	13.37 (4.24, 25.51)	3.28 (3.14, 3.42)	3.09 (2.96, 3.22)
**Diet low in fruits**										
Andean Latin America	34 (7, 82)	0.18 (0.04,0.42)	837 (171, 1966)	3.9 (0.79,9.23)	54 (7, 160)	0.10 (0.01,0.29)	1144 (133, 3444)	2.02 (0.23,6.07)	-2.04 (-2.14, -1.94)	-2.34 (-2.45, -2.23)

Region	1990				2019				AAPC(1990-2019)	
	Deaths (95%UI)	ASMR (95%UI)	DALYs (95%UI)	ASDR (95%UI)	Deaths (95%UI)	ASMR (95%UI)	DALYs (95%UI)	ASDR (95%UI)	ASMR (95%CI)	ASDR (95%CI)
Australasia	108	0.46	2363	10.17	182	0.36	3557	7.68	-1.02	-1.07
	(21, 259)	(0.09,1.1)	(473, 5661)	(2.04,24.35)	(26, 487)	(0.05,0.96)	(527, 9454)	(1.17,20.36)	(-1.08, -0.95)	(-1.12, -1.02)
Caribbean	74	0.29	1765	6.68	98	0.19	2461	4.73	-1.68	-1.35
	(10, 210)	(0.04,0.83)	(247, 5006)	(0.93,18.94)	(13, 289)	(0.03,0.56)	(354, 7269)	(0.69,13.94)	(-2.02, -1.35)	(-1.67, -1.02)
Central Asia	1150	2.54	29548	61.16	514	0.76	13435	17.25	-4.91	-5.13
	(405, 1982)	(0.9,4.35)	(10395, 50505)	(21.62,105.01)	(137, 1180)	(0.2,1.72)	(3503, 30916)	(4.6,39.51)	(-5.18, -4.64)	(-5.41, -4.86)
Central Europe	487	0.34	13019	8.90	554	0.27	13675	7.21	-1.02	-1.07
	(117, 1109)	(0.08,0.76)	(3080, 29644)	(2.13,20.12)	(106, 1352)	(0.05,0.66)	(2610, 33575)	(1.41,17.57)	(-1.24, -0.8)	(-1.33, -0.82)
Central Latin	136	0.17	3364	3.81	240	0.1	5551	2.31	-1.97	-1.89
America	(18, 399)	(0.02,0.51)	(461, 9663)	(0.51,11.14)	(34, 706)	(0.01,0.31)	(814, 16298)	(0.34,6.8)	(-2.11, -1.84)	(-2.04, -1.74)
Central Sub-Saharan	296	1.35	8738	33.95	695	1.37	19937	33.20	-0.30	-0.46
Africa	(63, 708)	(0.29,3.21)	(1925, 20446)	(7.4,80.41)	(196, 1397)	(0.39,2.75)	(5767, 39663)	(9.38,66.73)	(-0.71, 0.1)	(-0.86, -0.06)
East Asia	31106	3.74	800308	86.67	19948	1.0	447377	21.04	-4.95	-5.32
	(9699, 56128)	(1.17,6.74)	(251143, 1433077)	(27.29,155.25)	(3345, 52997)	(0.17,2.63)	(72880, 1182104)	(3.55,55.34)	(-5.54, -4.35)	(-5.94, -4.7)
Eastern Europe	2012	0.71	53488	18.79	1316	0.39	34712	10.6	-2.86	-2.77
	(645, 3627)	(0.23,1.28)	(17128, 96194)	(6.06,33.71)	(328, 2855)	(0.1,0.84)	(8590, 74607)	(2.68,22.53)	(-3.14, -2.58)	(-3.06, -2.47)
Eastern Sub-Saharan	1342	1.82	38841	46.46	2266	1.42	64945	35.31	-1.03	-1.15
Africa	(525, 2302)	(0.71,3.14)	(15387, 66162)	(18.13,79.67)	(802, 4129)	(0.5,2.62)	(23263, 118506)	(12.53,64.53)	(-1.11, -0.95)	(-1.24, -1.06)
High-income Asia	1319	0.66	31822	15.36	1794	0.40	35021	9.11	-1.59	-1.72
Pacific	(356, 2770)	(0.18,1.38)	(8712, 66560)	(4.19,32.11)	(437, 4019)	(0.1,0.88)	(9216, 77203)	(2.54, 19.36)	(-1.81, -1.37)	(-1.96, -1.47)

Region	1990				2019				AAPC(1990-2019)	
	Deaths (95%UI)	ASMR (95%UI)	DALYs (95%UI)	ASDR (95%UI)	Deaths (95%UI)	ASMR (95%UI)	DALYs (95%UI)	ASDR (95%UI)	ASMR (95%CI)	ASDR (95%CI)
High-income North America	1144 (189, 2962)	0.33 (0.06,0.86)	27132 (4605, 70020)	8.29 (1.43,21.37)	1823 (278, 4987)	0.29 (0.04,0.8)	40163 (6054, 110370)	6.89 (1.08,18.68)	-0.42 (-0.54, -0.29)	-0.63 (-0.76, -0.51)
North Africa and Middle East	310 (67, 761)	0.19 (0.04,0.45)	8751 (1863, 21568)	4.62 (1,11.36)	527 (110, 1351)	0.12 (0.03,0.32)	14959 (3203, 37083)	3.04 (0.63,7.66)	-1.8 (-1.96, -1.64)	-1.84 (-2.01, -1.67)
Oceania	10 (3, 20)	0.38 (0.12,0.72)	303 (99, 568)	9.05 (2.95,17.1)	22 (6, 44)	0.33 (0.09,0.68)	632 (177, 1263)	7.89 (2.24,15.8)	-0.36 (-0.41, -0.31)	-0.39 (-0.45, -0.33)
South Asia	6910 (3371, 10207)	1.27 (0.61,1.87)	199638 (97823, 294362)	31.52 (15.37,46.41)	13797 (6217, 21505)	1.00 (0.45,1.56)	376083 (171024, 579225)	25.04 (11.35,38.65)	-1.06 (-1.21, -0.92)	-1 (-1.12, -0.88)
Southeast Asia	1261 (479, 2162)	0.50 (0.19,0.86)	35663 (13484, 60686)	12.64 (4.77,21.64)	1664 (425, 3565)	0.28 (0.07,0.6)	44196 (11571, 94532)	6.74 (1.76,14.38)	-2.07 (-2.11, -2.03)	-2.24 (-2.28, -2.2)
Southern Latin America	290 (40, 802)	0.65 (0.09,1.78)	6549 (873, 18266)	14.15 (1.89,39.38)	185 (21, 594)	0.22 (0.02,0.7)	3562 (395, 11776)	4.33 (0.48,14.31)	-4.32 (-4.58, -4.05)	-4.68 (-4.97, -4.38)
Southern Sub-Saharan Africa	844 (358, 1354)	3.1 (1.32,4.96)	24086 (10191, 38618)	80.11 (33.9,129.06)	1349 (567, 2103)	2.49 (1.03,3.88)	35712 (15241, 55837)	59.41 (25.25,92.54)	-1.3 (-1.88, -0.72)	-1.62 (-2.21, -1.03)
Tropical Latin America	425 (63, 1201)	0.47 (0.07,1.32)	12013 (1811, 33339)	12 (1.78,33.75)	518 (61, 1676)	0.21 (0.03,0.7)	13461 (1552, 43696)	5.37 (0.62,17.36)	-2.97 (-3.14, -2.81)	-3.05 (-3.21, -2.9)
Western Europe	2306 (527, 5516)	0.41 (0.09,0.98)	52133 (11482, 127153)	9.74 (2.13,23.77)	2893 (592, 7133)	0.32 (0.06,0.79)	58338 (11811, 146226)	7.37 (1.55,18.5)	-1.03 (-1.12, -0.94)	-1.21 (-1.32, -1.1)
Western Sub-Saharan Africa	304 (121, 539)	0.36 (0.14,0.63)	8158 (3256, 14512)	8.68 (3.46,15.44)	773 (294, 1391)	0.43 (0.16,0.78)	20853 (7780, 37679)	10.2 (3.86,18.36)	0.97 (0.84, 1.1)	0.84 (0.73, 0.96)

Region	1990				2019				AAPC(1990-2019)	
	Deaths (95%UI)	ASMR (95%UI)	DALYs (95%UI)	ASDR (95%UI)	Deaths (95%UI)	ASMR (95%UI)	DALYs (95%UI)	ASDR (95%UI)	ASMR (95%CI)	ASDR (95%CI)
**Diet low in vegetables**										
Andean Latin	41	0.21	958	4.56	83	0.15	1716	3.06	-1.08	-1.38
America	(6,76)	(0.03,0.4)	(154,1810)	(0.73,8.59)	(13,165)	(0.02,0.3)	(254,3471)	(0.46,6.19)	(-1.16, -1)	(-1.47, -1.28)
Australasia	69	0.29	1487	6.36	126	0.25	2381	5.04	-0.67	-0.79
	(3,145)	(0.01,0.62)	(65,3118)	(0.28,13.34)	(5,269)	(0.01,0.53)	(98,5128)	(0.21,10.84)	(-0.74, -0.6)	(-0.85, -0.73)
Caribbean	95	0.37	2240	8.53	133	0.26	3189	6.13	-1.46	-1.28
	(15,181)	(0.06,0.71)	(362,4295)	(1.38,16.33)	(18,270)	(0.04,0.52)	(435,6553)	(0.84,12.61)	(-1.7, -1.23)	(-1.52, -1.04)
Central Asia	257	0.58	6243	13.19	70	0.11	1628	2.26	-7.12	-7.72
	(24,577)	(0.05,1.29)	(578,14256)	(1.23,29.89)	(15,145)	(0.02,0.24)	(357,3393)	(0.48,4.68)	(-7.78, -6.46)	(-8.44, -6.99)
Central Europe	189	0.13	4917	3.34	158	0.08	3608	1.83	-2.23	-2.5
	(10,418)	(0.01,0.29)	(256,10933)	(0.17,7.43)	(13,359)	(0.01,0.17)	(327,8238)	(0.17,4.21)	(-2.39, -2.07)	(-2.69, -2.31)
Central Latin	178	0.23	4227	4.94	330	0.14	7313	3.07	-1.91	-1.89
America	(22,349)	(0.03,0.46)	(497,8311)	(0.59,9.7)	(33,664)	(0.01,0.29)	(725,14709)	(0.3,6.17)	(-2.06, -1.77)	(-2.04, -1.73)
Central Sub-Saharan	285	1.31	8149	32.23	491	0.98	13819	23.43	-1.24	-1.37
Africa	(49,554)	(0.23,2.59)	(1360,15812)	(5.5,62.58)	(82,998)	(0.16,2.01)	(2329,28063)	(3.93,47.52)	(-1.34, -1.14)	(-1.49, -1.26)
East Asia	11769	1.42	294635	32.28	1293	0.07	26415	1.28	-11	-11.81
	(596,25367)	(0.07,3.09)	(14415,639948)	(1.61,69.66)	(508,2856)	(0.03,0.16)	(11280,56355)	(0.53,2.82)	(-11.32, -10.67)	(-12.21, -11.41)
Eastern Europe	536	0.19	13819	4.85	478	0.14	12054	3.60	-1.25	-1.17
	(28,1185)	(0.01,0.42)	(715,30655)	(0.25,10.78)	(26,1074)	(0.01,0.31)	(685,27396)	(0.2,8.15)	(-1.72, -0.76)	(-1.67, -0.67)

Region	1990				2019				AAPC(1990-2019)	
	Deaths (95%UI)	ASMR (95%UI)	DALYs (95%UI)	ASDR (95%UI)	Deaths (95%UI)	ASMR (95%UI)	DALYs (95%UI)	ASDR (95%UI)	ASMR (95%CI)	ASDR (95%CI)
Eastern Sub-Saharan Africa	926 (170,1710)	1.28 (0.23,2.39)	26218 (4832,47909)	31.83 (5.86,58.56)	1716 (328,3380)	1.1 (0.21,2.17)	48057 (9095,94123)	26.67 (5.1,52.23)	-0.63 (-0.71, -0.54)	-0.73 (-0.83, -0.64)
High-income Asia Pacific	320 (22,730)	0.16 (0.01,0.37)	7234 (522,16545)	3.51 (0.25,8.01)	298 (34,764)	0.06 (0.01,0.16)	5129 (637,13386)	1.26 (0.16, 3.26)	-2.93 (-3.32, -2.54)	-3.09 (-3.47, -2.71)
High-income North America	605 (31,1322)	0.17 (0.01,0.38)	13704 (680,30233)	4.13 (0.21,9.13)	1359 (103,2791)	0.21 (0.02,0.44)	28527 (2101,58987)	4.77 (0.35,9.87)	1.08 (0.88, 1.28)	0.86 (0.67, 1.05)
North Africa and Middle East	232 (29,486)	0.14 (0.02,0.3)	6159 (729,12884)	3.36 (0.41,7.05)	352 (51,751)	0.09 (0.01,0.19)	9267 (1339,20001)	1.96 (0.28,4.23)	-2.08 (-2.28, -1.88)	-2.23 (-2.46, -2.01)
Oceania	6 (1,13)	0.23 (0.04,0.46)	176 (30,359)	5.36 (0.92,11)	14 (2,29)	0.22 (0.04,0.46)	400 (66,824)	5.11 (0.84,10.6)	-0.09 (-0.12, -0.06)	-0.13 (-0.17, -0.08)
South Asia	2470 (402,4786)	0.46 (0.08,0.89)	70444 (11322,136830)	11.25 (1.83,21.82)	4619 (573,9116)	0.34 (0.04,0.66)	124896 (15207,247032)	8.34 (1.02,16.45)	-1.29 (-1.42, -1.16)	-1.19 (-1.29, -1.08)
Southeast Asia	721 (115,1370)	0.29 (0.05,0.55)	20007 (3155,38074)	7.18 (1.14,13.63)	1222 (128,2450)	0.21 (0.02,0.42)	31557 (3190,63451)	4.87 (0.5,9.75)	-1.32 (-1.4, -1.24)	-1.53 (-1.61, -1.44)
Southern Latin America	281 (24,567)	0.63 (0.05,1.26)	6303 (495,12688)	13.59 (1.08,27.36)	263 (14,556)	0.31 (0.02,0.66)	5247 (262,11117)	6.36 (0.32,13.45)	-2.71 (-2.85, -2.56)	-2.93 (-3.07, -2.78)
Southern Sub-Saharan Africa	363 (63,716)	1.34 (0.23,2.66)	10209 (1727,19984)	34.2 (5.83,67.11)	578 (99,1119)	1.07 (0.18,2.08)	15147 (2572,29490)	25.32 (4.3,49.15)	-1.38 (-1.95, -0.81)	-1.69 (-2.27, -1.11)
Tropical Latin America	595 (94,1151)	0.67 (0.11,1.29)	16219 (2454,31369)	16.53 (2.54,31.96)	1065 (97,2140)	0.44 (0.04,0.88)	27144 (2430,54321)	10.84 (0.97,21.7)	-1.51 (-1.58, -1.45)	-1.54 (-1.59, -1.48)

Region	1990				2019				AAPC(1990-2019)	
	Deaths (95%UI)	ASMR (95%UI)	DALYs (95%UI)	ASDR (95%UI)	Deaths (95%UI)	ASMR (95%UI)	DALYs (95%UI)	ASDR (95%UI)	ASMR (95%CI)	ASDR (95%CI)
Western Europe	1468 (88,3065)	0.26 (0.02,0.54)	32716 (1854,68705)	6.07 (0.34,12.76)	2072 (115,4384)	0.23 (0.01,0.48)	40691 (2106,86235)	5.02 (0.25,10.73)	-0.5 (-0.59, -0.41)	-0.73 (-0.84, -0.63)
Western Sub-Saharan Africa	185 (29,354)	0.22 (0.03,0.42)	4931 (770,9476)	5.27 (0.82,10.09)	455 (75,904)	0.26 (0.04,0.51)	12126 (1976,24302)	6.00 (0.99,11.88)	0.81 (0.71, 0.92)	0.66 (0.57, 0.75)

ASMR: age-standardized mortality rate; DALYs: disability-adjusted life years; ASDR:age-standardized DALY rate; age-standardized mortality rate and age-standardized DALY rate are showed per 100,000 persons. BMI: body-mass index; Behavioral risk includes smoking, alcohol use and chewing tobacco; metabolic risk include high body-mass index; diet risk include diet low in fruits and vegetables, respectively.

Table S2. The burden of EC attributable to behavioral, metabolic and diet factors in 5 SDI levels in 1990 and 2019

Region	1990				2019				AAPC (1990-2019)	
	Deaths (95%UI)	ASMR (95%UI)	DALYs (95%UI)	ASDR (95%UI)	Deaths (95%UI)	ASMR (95%UI)	DALYs (95%UI)	ASDR (95%UI)	ASMR (95%CI)	ASDR (95%CI)
Smoking										
Low SDI	3469 (2638,4317)	1.51 (1.16,1.87)	95609 (71717,119738)	37.35 (28.24,46.55)	5954 (4450,7793)	1.18 (0.89,1.53)	163428 (118926,216677)	29.33 (21.73,38.52)	-0.93 (-1.01,-0.84)	-0.91 (-1,-0.81)
Low-middle SDI	10953 (9205,14893)	1.88 (1.6,2.57)	298977 (248538,400474)	46.39 (38.81,62.74)	18766 (15342,29170)	1.4 (1.15,2.20)	481956 (391375,739373)	33.72 (27.46,51.71)	-1.12 (-1.22,-1.02)	-1.18 (-1.28,-1.09)
Middle SDI	56189 (33025,67497)	5.61 (3.36,6.72)	1018858 (838185,1167593)	135.13 (79.41,162.86)	82580 (64267,100310)	3.41 (2.64,4.14)	1924974 (1514168,2332737)	74.66 (58.57,90.4)	-1.96 (-2.38,-1.54)	-2.28 (-2.71,-1.85)
High-middle SDI	39344 (32457,44941)	3.66 (3.02,4.18)	1473309 (858889,1795336)	92.06 (75.94,105.47)	62576 (47383,75745)	3.03 (2.3,3.67)	717168 (639679,797819)	70.49 (54.07,85.52)	-0.90 (-1.17,-0.63)	-1.16 (-1.45,-0.86)
High SDI	24696 (22586,26691)	2.38 (2.18,2.57)	587800 (541269,632069)	58.43 (53.84,62.79)	33401 (29392,37683)	1.78 (1.58,2.00)	1457734 (1118093,1772470)	41.47 (37.1,46.1)	-1.13 (-1.25,-1.01)	-1.32 (-1.43,-1.2)
Alcohol use										
Low SDI	1827 (1184,2488)	0.75 (0.48,1.02)	53981 (35125,72986)	20.1 (13.04,27.29)	3858 (2591,5453)	0.72 (0.49,1.02)	114199 (76590,162080)	19.45 (13.01,27.59)	-0.17 (-0.27,-0.06)	-0.18 (-0.29,-0.08)
Low-middle SDI	3337 (2358,4653)	0.53 (0.37,0.75)	99906 (71002,138506)	14.66 (10.39,20.36)	8311 (5861,12693)	0.59 (0.42,0.91)	237411 (168099,358550)	16.03 (11.35,24.29)	0.36 (0.32,0.39)	0.3 (0.27,0.34)
Middle SDI	24247 (13499,33461)	2.32 (1.31,3.2)	574609 (427165,731505)	60.26 (33.4,82.57)	41630 (29448,55416)	1.68 (1.18,2.24)	1042444 (746685,1383493)	39.62 (28.31,52.38)	-1.31 (-1.64,-0.99)	-1.63 (-1.98,-1.29)
High-middle SDI	20985	1.94	680421	51.57	35373	1.72	541122	42.85	-0.60	-0.82

Region	1990				2019				AAPC (1990-2019)	
	Deaths (95%UI)	ASMR (95%UI)	DALYs (95%UI)	ASDR (95%UI)	Deaths (95%UI)	ASMR (95%UI)	DALYs (95%UI)	ASDR (95%UI)	ASMR (95%CI)	ASDR (95%CI)
	(15390,26709)	(1.42,2.46)	(378522,930099)	(38.27,65.63)	(25281,46457)	(1.22,2.25)	(417158,657022)	(30.9,55.95)	(-0.82,-0.38)	(-1.07,-0.58)
High SDI	15255	1.49	382748	38.57	24397	1.32	882186	31.88	-0.55	-0.81
	(11954,18407)	(1.17,1.80)	(301103,460559)	(30.37,46.41)	(18670,29696)	(1.01,1.61)	(636353,1151807)	(24.59,38.67)	(-0.64,-0.46)	(-0.89,-0.72)
Chewing tobacco										
Low SDI	1235	0.54	35433	13.54	2434	0.49	67253	11.94	-0.45	-0.54
	(855,1746)	(0.37,0.76)	(24465,49432)	(9.35,19.21)	(1689,3582)	(0.34,0.73)	(46207,96334)	(8.21,17.43)	(-0.54,-0.36)	(-0.64,-0.44)
Low-middle SDI	4367	0.75	123349	18.8	8435	0.63	225314	15.55	-0.76	-0.78
	(3110,5945)	(0.54,1.01)	(87297,168272)	(13.41,25.63)	(5995,12370)	(0.45,0.92)	(159600,325388)	(11.03,22.6)	(-0.88,-0.65)	(-0.89,-0.68)
Middle SDI	2065	0.21	26629	5.16	4230	0.17	107572	4.1	-0.82	-1.04
	(1400,2851)	(0.14,0.28)	(16603,37676)	(3.46,7.18)	(2847,5693)	(0.12,0.23)	(71597,147707)	(2.73,5.58)	(-1.01,-0.63)	(-1.25,-0.84)
High-middle SDI	964	0.09	57701	2.41	1888	0.09	28266	2.32	-0.12	-0.37
	(622,1350)	(0.06,0.13)	(38377,81195)	(1.51,3.41)	(1175,2669)	(0.06,0.13)	(15720,42674)	(1.43,3.33)	(-0.34,0.10)	(-0.61,-0.12)
High SDI	767	0.07	17928	1.78	1287	0.07	47393	1.67	-0.38	-0.35
	(439,1137)	(0.04,0.11)	(10130,26755)	(1.01,2.67)	(725,1946)	(0.04,0.10)	(29123,68001)	(0.93,2.53)	(-0.51,-0.24)	(-0.45,-0.25)
High BMI										
Low SDI	1179	0.5	34313	12.93	4112	0.79	119207	20.56	1.69	1.69
	(285,2706)	(0.12,1.16)	(8341,78611)	(3.13,29.6)	(1201,8250)	(0.23,1.58)	(35643,239878)	(6.06,41.46)	(1.59,1.78)	(1.6,1.79)
Low-middle SDI	2087	0.36	59062	8.98	9074	0.66	251342	17.14	2.20	2.32
	(508,5110)	(0.08,0.87)	(14438,144513)	(2.19,21.99)	(2988,17833)	(0.22,1.31)	(82449,491135)	(5.6,33.54)	(2.10,2.30)	(2.23,2.41)
Middle SDI	11082	1.11	295329	26.94	31024	1.27	765974	29.29	0.27	0.08

Region	1990				2019				AAPC (1990-2019)	
	Deaths (95%UI)	ASMR (95%UI)	DALYs (95%UI)	ASDR (95%UI)	Deaths (95%UI)	ASMR (95%UI)	DALYs (95%UI)	ASDR (95%UI)	ASMR (95%CI)	ASDR (95%CI)
	(2424,27789)	(0.24,2.81)	(82725,622129)	(6.04,66.87)	(9180,62632)	(0.38,2.57)	(224730,1518192)	(8.53,58.12)	(-0.14,0.68)	(-0.34,0.51)
High-middle SDI	11342<br>(3447,23743)	1.07<br>(0.33,2.23)	296557<br>(66679,728520)	26.68<br>(7.58,55.93)	25376<br>(7580,49886)	1.23<br>(0.37,2.42)	448011<br>(145695,777638)	29.97<br>(8.85,58.63)	0.33<br>(0.15,0.51)	0.22<br>(0.01,0.43)
High SDI	9580<br>(2916,17918)	0.93<br>(0.28,1.74)	232324<br>(70647,432300)	23.26<br>(7.05,43.3)	20284<br>(6693,35573)	1.10<br>(0.36,1.92)	616917<br>(182197,1207438)	26.4<br>(8.44,45.83)	0.45<br>(0.31,0.59)	0.31<br>(0.18,0.45)
Diet low in vegetables										
Low SDI	1559<br>(271,2884)	0.68<br>(0.12,1.27)	44185<br>(7631,81517)	16.98<br>(2.95,31.4)	2918<br>(542,5635)	0.58<br>(0.11,1.11)	81561<br>(15172,157287)	14.34<br>(2.67,27.67)	-0.67<br>(-0.74,-0.6)	-0.71<br>(-0.79,-0.63)
Low-middle SDI	2733<br>(438,5408)	0.48<br>(0.08,0.94)	75767<br>(12266,151010)	11.67<br>(1.88,23.17)	4385<br>(612,8543)	0.33<br>(0.05,0.64)	117224<br>(16243,228658)	8.08<br>(1.12,15.7)	-1.47<br>(-1.55,-1.38)	-1.41<br>(-1.50,-1.33)
Middle SDI	9725<br>(731,20600)	1.00<br>(0.08,2.13)	123555<br>(7951,266011)	23.13<br>(1.74,48.91)	3853<br>(639,7531)	0.16<br>(0.03,0.32)	93317<br>(15090,183132)	3.62<br>(0.59,7.10)	-6.82<br>(-7.11,-6.52)	-6.99<br>(-7.36,-6.62)
High-middle SDI	4972<br>(318,10650)	0.47<br>(0.03,1.01)	248321<br>(18676,527086)	11.27<br>(0.73,24.28)	2166<br>(368,4396)	0.11<br>(0.02,0.22)	77836<br>(5104,166338)	2.45<br>(0.41,4.93)	-5.50<br>(-5.97,-5.04)	-5.60<br>(-6.13,-5.07)
High SDI	2596<br>(148,5500)	0.25<br>(0.01,0.53)	59006<br>(3233,125758)	5.85<br>(0.32,12.47)	3844<br>(258,8108)	0.20<br>(0.01,0.43)	50118<br>(8331,100745)	4.47<br>(0.29,9.56)	-0.65<br>(-0.70,-0.60)	-0.81<br>(-0.87,-0.76)
Diet low in fruits										
Low SDI	2404<br>(938,4126)	1.04<br>(0.40,1.78)	69634<br>(27137,118521)	26.39<br>(10.28,45.16)	4621<br>(1747,7820)	0.91<br>(0.34,1.55)	130947<br>(49730,221231)	22.76<br>(8.59,38.53)	-0.58<br>(-0.69,-0.46)	-0.64<br>(-0.76,-0.53)
Low-middle SDI	6888	1.18	195273	29.64	11400	0.85	309350	21.21	-1.38	-1.36

Region	1990				2019				AAPC (1990-2019)	
	Deaths (95%UI)	ASMR (95%UI)	DALYs (95%UI)	ASDR (95%UI)	Deaths (95%UI)	ASMR (95%UI)	DALYs (95%UI)	ASDR (95%UI)	ASMR (95%CI)	ASDR (95%CI)
	(3103,10731)	(0.53,1.85)	(89117,301896)	(13.4,46.02)	(4969,19129)	(0.37,1.42)	(137100,513684)	(9.35,35.36)	(-1.51,-1.25)	(-1.48,-1.25)
Middle SDI	24518 (8095,43210)	2.50 (0.83,4.40)	334042 (103539,621615)	58.85 (19.62,103.77)	17982 (4599,41528)	0.76 (0.20,1.76)	424189 (111095,966353)	16.53 (4.31,37.74)	-4.52 (-5.04,-4.01)	-4.83 (-5.34,-4.31)
High-middle SDI	12968 (3881,24354)	1.22 (0.37,2.30)	640223 (215623,1127963)	30.30 (9.41,56.49)	10205 (2269,25842)	0.50 (0.11,1.26)	145475 (29959,359444)	11.73 (2.69,28.76)	-3.60 (-4.00,-3.21)	-3.81 (-4.23,-3.39)
High SDI	5081 (1156,11701)	0.49 (0.11,1.13)	119145 (26529,275861)	11.89 (2.65,27.50)	6990 (1411,17264)	0.37 (0.08,0.91)	239523 (54619,587643)	8.48 (1.78,20.95)	-1.05 (-1.12,-0.98)	-1.26 (-1.34,-1.18)

(ASMR: age-standardized mortality rate; DALYs: disability-adjusted life years; ASDR:age-standardized DALY rate; age-standardized mortality rate and age-standardized DALY rate are showed per 100 000 persons. BMI: body-mass index; Behavioral risk includes smoking, alcohol use and chewing tobacco; metabolic risk include high body-mass index; diet risk include diet low in fruits and vegetables, respectively

Table S3. The burden of EC attributable to bahavioral, metobolic, and diet risk facors for both sexes in 204 countries and territories in 2019

Countries and territories	Smoking			
	Deaths (95%UI)	ASMR (95%UI)	DALYs (95%UI)	ASDR (95%UI)
Afghanistan	132 (44, 196)	1.09 (0.38, 1.59)	3919 (1322, 6054)	26.52 (8.91, 39.41)
Albania	33 (23, 47)	0.76 (0.54, 1.08)	715 (502, 1101)	17.06 (12.01, 26)
Algeria	141 (98, 186)	0.47 (0.33, 0.62)	3235 (2198, 4320)	9.56 (6.58, 12.68)
American Samoa	0 (0, 0)	0.52 (0.39, 0.64)	7 (5, 8)	12.87 (9.35, 15.96)
Andorra	2 (2, 3)	1.57 (1.14, 2.1)	54 (39, 74)	38.56 (27.57, 52.2)
Angola	212 (116, 300)	1.88 (1.03, 2.67)	6161 (3397, 8826)	47.89 (26.33,
Antigua and Barbuda	1 (1, 1)	0.68 (0.51, 0.87)	17 (12, 21)	15.59 (11.74, 19.95)
Argentina	982 (831, 1148)	1.82 (1.54, 2.12)	22779 (19291,	43.4 (36.76, 50.12)
Armenia	29 (23, 36)	0.7 (0.56, 0.85)	701 (551, 871)	16.5 (13.01, 20.5)
Australia	408 (334, 494)	1 (0.83, 1.2)	9080 (7528, 10745)	23.94 (19.97,
Austria	186 (159, 214)	1.11 (0.96, 1.28)	4333 (3716, 4981)	28.03 (23.93,
Azerbaijan	328 (234, 548)	3.73 (2.65, 6.31)	8631 (6106, 14231)	84.73 (61, 139.82)
Bahamas	5 (3, 6)	1.19 (0.88, 1.56)	129 (94, 173)	30.11 (22.13,
Bahrain	6 (4, 8)	0.83 (0.58, 1.1)	153 (104, 213)	15.48 (10.7, 20.8)
Bangladesh	1677 (1117, 2572)	1.31 (0.88, 1.99)	42304 (27755,	31.33 (20.66,
Barbados	5 (4, 7)	1.05 (0.8, 1.34)	113 (85, 147)	23.67 (17.8, 30.71)
Belarus	250 (187, 336)	1.57 (1.18, 2.1)	6865 (5112, 9252)	44.15 (32.78,
Belgium	428 (363, 491)	1.96 (1.67, 2.23)	9329 (8028, 10680)	46.83 (40.29,
Belize	2 (2, 2)	0.72 (0.57, 0.89)	55 (43, 69)	18.38 (14.29,
Benin	40 (22, 61)	0.85 (0.45, 1.26)	1082 (581, 1664)	21.02 (11.3, 32.06)
Bermuda	2 (1, 2)	1.19 (0.88, 1.56)	35 (26, 45)	28.69 (21.34,
Bhutan	5 (3, 8)	0.98 (0.62, 1.42)	122 (75, 180)	21.45 (13.31,
Bolivia (Plurinational State of)	43 (29, 60)	0.5 (0.35, 0.69)	1005 (673, 1423)	11.14 (7.51, 15.75)
Bosnia and Herzegovina	66 (49, 85)	1.09 (0.82, 1.4)	1614 (1201, 2105)	27.4 (20.64, 35.81)
Botswana	57 (35, 78)	4.14 (2.54, 5.6)	1621 (979, 2319)	105.13 (64.6,
Brazil	3772 (3204, 4355)	1.58 (1.34, 1.83)	96233 (81483, 111694)	39.27 (33.34, 45.58)
Brunei	3 (2, 4)	1.09 (0.86, 1.41)	71 (56, 92)	22.88 (17.95,
Bulgaria	127 (95, 165)	0.97 (0.72, 1.27)	3391 (2499, 4430)	27.93 (20.54,
Burkina Faso	59 (30, 85)	0.62 (0.32, 0.89)	1736 (892, 2547)	16.77 (8.64, 24.41)
Burundi	99 (59, 159)	2.1 (1.28, 3.3)	2885 (1677, 4720)	55.27 (32.78,
Cabo Verde	11 (8, 15)	2.67 (1.98, 3.47)	316 (227, 423)	70.87 (50.92,
Cambodia	133 (98, 189)	1.17 (0.87, 1.68)	3370 (2425, 4880)	27.01 (19.67,
Cameroon	131 (66, 203)	1.06 (0.53, 1.63)	3857 (1915, 6033)	28.55 (14.31,
Canada	988 (819, 1173)	1.42 (1.18, 1.67)	20701 (17483,	31.53 (26.85,

Central African Republic	39 (20, 59)	1.65 (0.89, 2.44)	1202 (611, 1862)	45.75 (23.85, 69.16)
Chad	48 (24, 71)	0.87 (0.45, 1.29)	1303 (656, 1922)	21.7 (11, 31.9)
Chile	211 (167, 261)	0.87 (0.69, 1.08)	4661 (3788, 5623)	19.2 (15.58, 23.16)
China	123070 (93222, 153229)	6.16 (4.67, 7.63)	2801241 (2133864, 3501723)	133.46 (102.15, 166.24)
Colombia	191 (132, 264)	0.36 (0.25, 0.5)	4405 (3014, 6106)	8.33 (5.69, 11.54)
Comoros	11 (7, 16)	2.28 (1.45, 3.38)	280 (171, 436)	55.25 (33.84,
Congo	51 (29, 72)	1.98 (1.11, 2.86)	1438 (823, 2095)	48.8 (28.02, 70.08)
Cook Islands	0 (0, 0)	0.91 (0.7, 1.15)	6 (4, 7)	22.21 (16.81,
Costa Rica	23 (17, 31)	0.46 (0.32, 0.61)	497 (349, 670)	9.6 (6.73, 12.9)
Croatia	110 (82, 143)	1.32 (0.98, 1.73)	2612 (1924, 3447)	33.7 (24.69, 44.53)
Cuba	430 (331, 544)	2.27 (1.75, 2.87)	10909 (8384,	58.47 (45.02,
Cyprus	11 (8, 14)	0.58 (0.42, 0.72)	248 (184, 306)	12.84 (9.67, 15.9)
Czechia	279 (216, 354)	1.39 (1.07, 1.78)	6769 (5236, 8647)	36.25 (27.8, 46.25)
C么te d'Ivoire	124 (63, 182)	1.11 (0.57, 1.62)	3743 (1896, 5534)	29.79 (15.13,
Democratic People's Republic of Korea	1197 (896, 1604)	3.65 (2.75, 4.87)	32536 (23889, 44747)	96.65 (71.79, 131.57)
Democratic Republic of the Congo	464 (234, 735)	1.27 (0.65, 2.01)	13428 (6765, 21029)	33.3 (16.86, 52.66)
Denmark	237 (202, 279)	2.04 (1.74, 2.4)	4764 (4049, 5632)	44.26 (37.76,
Djibouti	22 (13, 38)	3.75 (2.22, 6.16)	645 (370, 1135)	93.37 (54.56,
Dominica	1 (1, 1)	1.07 (0.78, 1.42)	24 (17, 32)	26.88 (19.2, 35.85)
Dominican	82 (55, 115)	0.91 (0.62, 1.28)	1900 (1228, 2778)	20.18 (13.19, 29.2)
Ecuador	55 (39, 80)	0.38 (0.27, 0.55)	1180 (821, 1758)	7.78 (5.43, 11.54)
Egypt	392 (260, 548)	0.63 (0.43, 0.89)	10909 (7087,	15.42 (10.25,
El Salvador	16 (11, 22)	0.27 (0.19, 0.38)	380 (254, 526)	6.59 (4.4, 9.13)
Equatorial Guinea	8 (4, 15)	1.66 (0.86, 3.26)	212 (106, 417)	40.87 (20.45,
Eritrea	38 (21, 61)	1.22 (0.71, 1.91)	1244 (697, 2020)	35.92 (20.4, 57.55)
Estonia	31 (23, 42)	1.33 (0.97, 1.78)	791 (573, 1052)	36.05 (26.01, 47.8)
Eswatini	16 (9, 23)	2.72 (1.59, 4.05)	433 (242, 669)	69.19 (39.74,
Ethiopia	84 (50, 136)	0.21 (0.13, 0.34)	2200 (1314, 3636)	5.1 (3.05, 8.31)
Fiji	6 (3, 9)	0.84 (0.43, 1.15)	172 (86, 242)	20.78 (10.39,
Finland	95 (79, 115)	0.81 (0.67, 0.97)	2050 (1675, 2453)	19.34 (15.84,
France	1991 (1662, 2315)	1.58 (1.33, 1.83)	44561 (37740,	39.43 (33.31,
Gabon	22 (12, 32)	2.06 (1.11, 2.94)	629 (327, 919)	53.75 (28.39,
Gambia	4 (3, 6)	0.43 (0.29, 0.58)	117 (77, 164)	11.44 (7.65, 15.82)
Georgia	48 (38, 59)	0.82 (0.65, 1.01)	1204 (935, 1496)	21.71 (16.95,
Germany	2862 (2439, 3313)	1.61 (1.39, 1.85)	64362 (55385,	40.09 (34.3, 46.04)
Ghana	71 (45, 100)	0.45 (0.29, 0.63)	1916 (1204, 2774)	11.13 (7.07, 15.86)
Greece	174 (150, 199)	0.74 (0.66, 0.84)	3427 (3016, 3869)	17.28 (15.25,
Greenland	4 (3, 6)	6.3 (4.83, 7.88)	117 (87, 147)	152.01 (114.72, 190.69)
Grenada	2 (1, 2)	1.66 (1.27, 2.05)	53 (41, 66)	43.42 (33.2, 53.54)

Guam	2 (1, 2)	0.84 (0.62, 1.09)	44 (32, 57)	22.46 (16.01,
Guatemala	42 (30, 57)	0.4 (0.28, 0.54)	970 (671, 1361)	8.69 (6.05, 12.22)
Guinea	25 (17, 35)	0.45 (0.32, 0.63)	706 (483, 999)	12 (8.23, 16.82)
Guinea-Bissau	6 (3, 8)	0.74 (0.36, 1.08)	171 (84, 256)	20.61 (9.99, 30.43)
Guyana	3 (2, 5)	0.49 (0.34, 0.67)	98 (67, 135)	13.62 (9.38, 18.89)
Haiti	36 (21, 56)	0.5 (0.3, 0.78)	1033 (604, 1653)	13.32 (7.89, 21.11)
Honduras	31 (20, 42)	0.53 (0.35, 0.71)	726 (465, 1003)	11.82 (7.62, 16.29)
Hungary	304 (237, 386)	1.72 (1.33, 2.19)	8010 (6183, 10250)	48.02 (36.92,
Iceland	9 (7, 11)	1.63 (1.32, 1.97)	194 (159, 233)	37.46 (30.86,
India	9030 (6700, 14168)	0.81 (0.61, 1.27)	235598 (171767, 366951)	19.68 (14.46, 30.58)
Indonesia	1922 (1387, 2912)	0.92 (0.67, 1.41)	51065 (36409,	21.65 (15.6, 32.62)
Iran (Islamic Republic of)	846 (639, 991)	1.23 (0.92, 1.44)	20086 (15600, 23598)	26.67 (20.45, 31.2)
Iraq	110 (81, 138)	0.52 (0.39, 0.65)	2838 (2056, 3664)	11.73 (8.57, 15)
Ireland	189 (154, 226)	2.49 (2.04, 2.97)	3760 (3090, 4486)	51.34 (42.39,
Israel	63 (52, 75)	0.54 (0.45, 0.63)	1253 (1064, 1467)	11.32 (9.58, 13.25)
Italy	992 (850, 1150)	0.72 (0.62, 0.82)	20186 (17535,	16.59 (14.48,
Jamaica	22 (16, 29)	0.75 (0.55, 1)	510 (369, 685)	17.27 (12.45,
Japan	5791 (4962, 6646)	1.68 (1.46, 1.9)	110472 (96611, 125481)	37.55 (33.12, 42.55)
Jordan	33 (25, 43)	0.55 (0.43, 0.71)	864 (633, 1122)	12.21 (9.09, 15.82)
Kazakhstan	413 (326, 502)	2.37 (1.89, 2.87)	10763 (8435,	57.97 (45.75,
Kenya	460 (306, 687)	2.14 (1.44, 3.16)	12734 (8323,	52.79 (35.13,
Kiribati	3 (1, 4)	3.99 (1.7, 5.29)	79 (32, 109)	100.99 (41.12, 136.21)
Kuwait	13 (9, 17)	0.6 (0.45, 0.8)	288 (216, 374)	10.88 (8.14, 14.37)
Kyrgyzstan	75 (62, 89)	1.75 (1.45, 2.08)	1899 (1549, 2292)	40.23 (33.09,
Lao People's Democratic Republic	40 (28, 63)	0.97 (0.68, 1.5)	1074 (719, 1714)	22.97 (15.69, 35.91)
Latvia	56 (42, 75)	1.59 (1.17, 2.13)	1465 (1079, 1978)	44.53 (32.81,
Lebanon	34 (26, 45)	0.66 (0.49, 0.87)	759 (556, 1001)	14.64 (10.75,
Lesotho	65 (41, 86)	5.11 (3.25, 6.72)	1865 (1175, 2525)	134.56 (84.93, 180.12)
Liberia	18 (9, 27)	0.88 (0.44, 1.32)	521 (256, 788)	22.98 (11.44,
Libya	31 (21, 40)	0.62 (0.43, 0.81)	815 (538, 1089)	14.77 (9.91, 19.54)
Lithuania	82 (63, 105)	1.6 (1.21, 2.08)	2166 (1628, 2834)	45.08 (33.75,
Luxembourg	13 (10, 17)	1.34 (1.05, 1.68)	300 (236, 376)	31.74 (24.92,
Madagascar	150 (86, 242)	1.34 (0.79, 2.11)	4471 (2567, 7334)	34.62 (19.97, 56.2)
Malawi	424 (283, 615)	5.89 (3.99, 8.46)	11739 (7568, 17383)	151.05 (99.27, 221.13)
Malaysia	213 (157, 276)	0.84 (0.63, 1.09)	5147 (3717, 6753)	18.61 (13.65,
Maldives	2 (1, 2)	0.65 (0.51, 0.81)	42 (33, 53)	13.4 (10.32, 16.69)
Mali	43 (28, 63)	0.51 (0.34, 0.73)	1182 (744, 1739)	12.73 (8.15, 18.64)
Malta	8 (6, 10)	0.87 (0.69, 1.07)	175 (140, 215)	21.55 (17.49,

Marshall Islands	0 (0, 0)	0.79 (0.52, 1.16)	8 (5, 12)	20.07 (12.3, 29.88)
Mauritania	18 (10, 26)	0.86 (0.46, 1.26)	489 (259, 740)	21.98 (11.7, 32.82)
Mauritius	13 (10, 17)	0.76 (0.56, 0.99)	329 (242, 434)	17.89 (13.25,
Mexico	435 (331, 555)	0.38 (0.29, 0.49)	9973 (7370, 12897)	8.4 (6.22, 10.83)
Micronesia (Federated States of)	1 (1, 1)	1.27 (0.81, 1.79)	27 (17, 40)	33.34 (20.74, 48.04)
Monaco	2 (1, 2)	1.94 (1.45, 2.5)	37 (27, 48)	46.93 (34.55,
Mongolia	152 (112, 203)	7.56 (5.44, 9.94)	3853 (2799, 5336)	164.97 (121.84, 220.5)
Montenegro	13 (10, 16)	1.3 (1.02, 1.65)	335 (260, 429)	34.6 (26.82, 44.18)
Morocco	107 (73, 138)	0.35 (0.25, 0.45)	2725 (1840, 3637)	8.21 (5.58, 10.8)
Mozambique	214 (141, 304)	2.03 (1.34, 2.83)	5802 (3870, 8323)	49.94 (33.14,
Myanmar	384 (285, 598)	0.86 (0.65, 1.33)	9699 (7108, 14859)	19.89 (14.76,
Namibia	10 (7, 14)	0.76 (0.54, 1.05)	259 (172, 374)	17.89 (12.22,
Nauru	0 (0, 0)	1.16 (0.77, 1.62)	2 (1, 2)	30.12 (18.7, 42.65)
Nepal	370 (256, 516)	1.77 (1.25, 2.44)	8698 (5838, 12337)	38.37 (25.98,
Netherlands	1014 (856, 1191)	2.94 (2.49, 3.41)	20537 (17388,	63.77 (54.18,
New Zealand	90 (73, 109)	1.14 (0.92, 1.37)	1855 (1515, 2188)	24.96 (20.42, 29.2)
Nicaragua	13 (9, 17)	0.32 (0.23, 0.43)	293 (208, 405)	6.58 (4.75, 8.99)
Niger	32 (16, 50)	0.43 (0.22, 0.65)	902 (460, 1439)	10.49 (5.31, 16.33)
Nigeria	82 (47, 163)	0.1 (0.06, 0.19)	2226 (1233, 4580)	2.38 (1.37, 4.77)
Niue	0 (0, 0)	0.77 (0.55, 0.99)	0 (0, 1)	18.81 (13.16,
North Macedonia	26 (20, 34)	0.81 (0.62, 1.05)	676 (506, 886)	20.68 (15.51,
Northern Mariana Islands	1 (0, 1)	1.04 (0.7, 1.3)	15 (10, 20)	25.09 (16.76, 31.67)
Norway	57 (47, 68)	0.63 (0.52, 0.74)	1336 (1110, 1562)	15.62 (13.05,
Oman	8 (5, 10)	0.51 (0.38, 0.64)	215 (143, 300)	11.01 (7.73, 14.18)
Pakistan	2188 (1588, 2901)	2.01 (1.46, 2.61)	61263 (43214,	50.31 (35.89,
Palau	0 (0, 0)	0.69 (0.5, 0.91)	4 (3, 6)	18.03 (13.15,
Palestine	10 (8, 14)	0.46 (0.36, 0.65)	252 (194, 341)	10.14 (7.88, 14.09)
Panama	14 (10, 19)	0.34 (0.24, 0.47)	316 (216, 441)	7.64 (5.26, 10.65)
Papua New	26 (16, 42)	0.54 (0.35, 0.87)	766 (468, 1234)	13.97 (8.73, 22.58)
Paraguay	89 (62, 122)	1.66 (1.16, 2.27)	2067 (1440, 2830)	36.84 (25.64,
Peru	49 (31, 74)	0.15 (0.1, 0.23)	1057 (653, 1638)	3.32 (2.05, 5.15)
Philippines	407 (306, 567)	0.53 (0.4, 0.74)	11124 (8284,	13.06 (9.82, 18.28)
Poland	991 (783, 1238)	1.46 (1.15, 1.84)	24752 (19279,	38.52 (29.84,
Portugal	242 (205, 283)	1.2 (1.01, 1.39)	6314 (5330, 7359)	34.26 (28.75,
Puerto Rico	47 (33, 66)	0.67 (0.46, 0.93)	1008 (697, 1385)	15.82 (10.94,
Qatar	7 (4, 10)	1.52 (0.98, 2.22)	189 (118, 312)	23.78 (15.72,
Republic of Korea	1114 (911, 1547)	1.23 (1.01, 1.71)	23872 (19672,	25.93 (21.42,
Republic of Moldova	53 (43, 66)	0.91 (0.74, 1.12)	1506 (1217, 1861)	26.28 (21.11, 32.46)
Romania	371 (289, 464)	1.12 (0.86, 1.4)	10359 (7941,	32.97 (25.09,
Russian Federation	3591 (2862, 4381)	1.51 (1.21, 1.85)	96278 (76995, 118116)	41.62 (33.27, 51.18)

Rwanda	205 (130, 305)	3.68 (2.4, 5.37)	5247 (3249, 8006)	83.44 (52.67,
Saint Kitts and Nevis	1 (0, 1)	0.83 (0.63, 1.06)	16 (11, 21)	21.1 (15.42, 27.26)
Saint Lucia	3 (2, 3)	1.22 (0.92, 1.55)	71 (53, 90)	31.47 (23.63,
Saint Vincent and the Grenadines	1 (1, 1)	0.61 (0.47, 0.77)	23 (17, 29)	16.18 (12.25, 20.56)
Samoa	1 (1, 1)	0.66 (0.47, 0.82)	24 (17, 32)	15.99 (11.13,
San Marino	0 (0, 1)	0.58 (0.35, 0.86)	8 (5, 12)	13.66 (8.06, 20.98)
Sao Tome and Principe	1 (0, 1)	0.75 (0.38, 1.04)	22 (11, 33)	19.42 (9.95, 27.84)
Saudi Arabia	82 (58, 121)	0.49 (0.35, 0.7)	2478 (1708, 3669)	11.49 (8.23, 16.88)
Senegal	63 (33, 90)	0.82 (0.42, 1.17)	1784 (924, 2595)	21.8 (11.32, 31.62)
Serbia	180 (135, 235)	1.18 (0.89, 1.54)	4574 (3399, 6050)	31.72 (23.56,
Seychelles	3 (2, 3)	2.28 (1.83, 2.81)	67 (53, 84)	56.57 (45.09,
Sierra Leone	40 (20, 60)	1.12 (0.56, 1.65)	1128 (574, 1679)	29.22 (14.84,
Singapore	40 (32, 49)	0.52 (0.42, 0.63)	908 (735, 1108)	11.22 (9.04, 13.63)
Slovakia	120 (82, 164)	1.32 (0.91, 1.8)	3210 (2186, 4393)	36.52 (24.8, 49.86)
Slovenia	47 (34, 62)	1.16 (0.85, 1.55)	1137 (834, 1523)	30.26 (22.34,
Solomon Islands	4 (3, 6)	1.37 (0.97, 1.96)	133 (83, 200)	36.55 (24.68,
Somalia	148 (84, 224)	2.21 (1.27, 3.3)	4396 (2451, 6719)	58.75 (33.17,
South Africa	1284 (1023, 1659)	2.85 (2.29, 3.63)	35124 (27502,	73.07 (57.76,
South Sudan	76 (46, 128)	2.06 (1.24, 3.37)	2088 (1209, 3612)	49.94 (29.59,
Spain	1042 (903, 1202)	1.19 (1.03, 1.36)	24216 (20956,	30.02 (25.87,
Sri Lanka	257 (170, 375)	1 (0.67, 1.44)	6313 (4011, 9441)	23.57 (15.11,
Sudan	268 (86, 408)	1.59 (0.51, 2.41)	6581 (2101, 10228)	34.71 (11.25,
Suriname	3 (2, 4)	0.48 (0.36, 0.61)	80 (59, 105)	12.48 (9.28, 16.11)
Sweden	216 (179, 256)	1 (0.84, 1.17)	4072 (3404, 4738)	20.96 (17.79,
Switzerland	253 (213, 295)	1.45 (1.24, 1.69)	5065 (4343, 5901)	31.94 (27.51,
Syrian Arab Republic	45 (32, 61)	0.39 (0.29, 0.52)	1154 (792, 1604)	8.69 (6.12, 11.93)
Taiwan (Province of China)	1429 (1064, 1926)	3.64 (2.74, 4.9)	41813 (30905, 57072)	109.03 (80.61, 148.88)
Tajikistan	98 (67, 216)	2.25 (1.54, 4.86)	2635 (1784, 5728)	49.92 (34.09,
Thailand	1140 (724, 1586)	1.09 (0.7, 1.51)	29877 (18219,	28.1 (17.16, 39.89)
Timor-Leste	6 (4, 11)	0.82 (0.55, 1.31)	164 (102, 273)	19.34 (12.22,
Togo	39 (19, 57)	1.05 (0.53, 1.51)	1109 (563, 1648)	26.8 (13.39, 39.56)
Tokelau	0 (0, 0)	0.61 (0.44, 0.85)	0 (0, 0)	14.99 (10.28,
Tonga	1 (1, 1)	0.93 (0.67, 1.23)	17 (12, 23)	21.25 (14.96,
Trinidad and	9 (6, 12)	0.47 (0.33, 0.66)	230 (157, 325)	12.14 (8.35, 17.1)
Tunisia	55 (36, 77)	0.45 (0.3, 0.63)	1244 (806, 1776)	9.65 (6.32, 13.77)
Turkey	516 (387, 673)	0.58 (0.44, 0.76)	13461 (9874,	14.61 (10.77,
Turkmenistan	105 (79, 139)	2.71 (2.02, 3.57)	2910 (2157, 3836)	67.96 (50.78,
Tuvalu	0 (0, 0)	0.84 (0.6, 1.2)	2 (2, 3)	21.47 (14.74,
Uganda	376 (249, 531)	2.77 (1.87, 3.88)	10351 (6767,	68.29 (45.1, 96.75)
Ukraine	998 (757, 1269)	1.37 (1.04, 1.75)	28863 (21833,	41.07 (31.06,

United Arab Emirates	104 (29, 180)	2.5 (0.74, 4.36)	3783 (1031, 6579)	57.16 (16.28, 98.61)
United Kingdom	4082 (3497, 4710)	3.18 (2.75, 3.64)	78450 (68658,	67.17 (59.07,
United Republic of Tanzania	772 (461, 1245)	3.24 (1.95, 5.08)	20956 (12104, 34522)	80 (47.45, 130.53)
United States of America	9823 (8457, 11240)	1.75 (1.51, 1.99)	214668 (188969, 240192)	39.89 (35.31, 44.57)
United States Virgin Islands	2 (1, 2)	0.96 (0.69, 1.26)	43 (31, 57)	23.3 (16.45, 31.11)
Uruguay	113 (95, 134)	2.19 (1.84, 2.57)	2623 (2203, 3091)	54.24 (45.47,
Uzbekistan	306 (237, 383)	1.52 (1.19, 1.89)	8778 (6766, 11038)	36.65 (28.41,
Vanuatu	1 (1, 2)	0.77 (0.53, 1.12)	35 (23, 52)	18.96 (12.75,
Venezuela (Bolivarian Republic of)	159 (109, 217)	0.55 (0.38, 0.75)	3904 (2630, 5509)	12.93 (8.72, 18.08)
Viet Nam	1283 (859, 1687)	1.32 (0.91, 1.7)	36156 (22959,	34.64 (22.7, 45.85)
Yemen	191 (60, 283)	1.55 (0.49, 2.29)	5026 (1572, 7556)	35.75 (11.16,
Zambia	215 (136, 307)	3.43 (2.21, 4.85)	5840 (3627, 8459)	82.63 (51.87,
Zimbabwe	349 (261, 455)	5.12 (3.86, 6.63)	9728 (7037, 12923)	128.35 (94.83, 168.98)
Countries and territories	Alcohol use			
	Deaths (95%UI)	ASMR (95%UI)	DALYs (95%UI)	ASDR (95%UI)
Afghanistan	4 (1, 8)	0.03 (0.01, 0.05)	137 (33, 290)	0.78 (0.19, 1.63)
Albania	14 (8, 21)	0.33 (0.2, 0.49)	334 (206, 522)	8.27 (5.15, 12.83)
Algeria	15 (8, 23)	0.04 (0.02, 0.07)	444 (249, 689)	1.17 (0.66, 1.82)
American Samoa	0 (0, 0)	0.07 (0.02, 0.14)	1 (0, 2)	1.99 (0.59, 3.92)
Andorra	2 (1, 3)	1.39 (0.94, 1.89)	49 (33, 67)	34.98 (23.34,
Angola	254 (132, 384)	2.31 (1.19, 3.45)	7490 (3919, 11227)	57.69 (30, 86.99)
Antigua and Barbuda	1 (0, 1)	0.58 (0.41, 0.79)	16 (11, 22)	14.7 (10.33, 19.87)
Argentina	905 (688, 1127)	1.68 (1.28, 2.09)	20490 (15517,	39.05 (29.61, 48.3)
Armenia	10 (6, 14)	0.23 (0.15, 0.32)	260 (168, 360)	6.22 (4.03, 8.54)
Australia	604 (463, 751)	1.45 (1.1, 1.79)	12608 (9797,	32.57 (25.37, 39.9)
Austria	160 (124, 195)	0.96 (0.75, 1.17)	3725 (2935, 4502)	24.23 (19.07,
Azerbaijan	182 (112, 301)	1.94 (1.17, 3.21)	5161 (3253, 8347)	48.32 (30.13,
Bahamas	4 (2, 7)	1.07 (0.53, 1.63)	132 (68, 198)	30.18 (15.3, 45.33)
Bahrain	1 (1, 2)	0.09 (0.05, 0.14)	34 (19, 52)	2.31 (1.3, 3.52)
Bangladesh	88 (10, 180)	0.07 (0.01, 0.14)	2442 (293, 5045)	1.76 (0.21, 3.63)
Barbados	6 (4, 8)	1.28 (0.91, 1.71)	155 (111, 207)	32.8 (23.57, 43.76)
Belarus	191 (133, 262)	1.2 (0.84, 1.64)	5349 (3717, 7364)	34.63 (24.01,
Belgium	378 (294, 461)	1.74 (1.36, 2.11)	8349 (6509, 10130)	42.5 (33.22, 51.45)
Belize	2 (1, 2)	0.51 (0.37, 0.68)	46 (33, 60)	14.48 (10.45,
Benin	36 (18, 57)	0.72 (0.36, 1.16)	1039 (520, 1676)	19.02 (9.33, 30.67)
Bermuda	1 (1, 2)	1.17 (0.83, 1.56)	35 (25, 47)	29.49 (21.08,
Bhutan	2 (1, 3)	0.28 (0.12, 0.51)	46 (20, 83)	7.52 (3.25, 13.54)

Bolivia (Plurinational State of)	35 (20, 53)	0.39 (0.23, 0.6)	897 (535, 1353)	9.6 (5.7, 14.49)
Bosnia and Herzegovina	33 (23, 47)	0.56 (0.39, 0.78)	877 (601, 1226)	15.06 (10.34, 21)
Botswana	39 (22, 58)	2.69 (1.46, 3.97)	1205 (663, 1817)	73.62 (41.08,
Brazil	2822 (2078, 3582)	1.16 (0.85, 1.48)	80962 (60821, 102213)	32.63 (24.48, 41.21)
Brunei	0 (0, 0)	0.05 (0, 0.14)	4 (0, 12)	1.21 (0, 3.47)
Bulgaria	99 (69, 133)	0.75 (0.53, 1.01)	2597 (1826, 3488)	21.61 (15.3, 29.2)
Burkina Faso	124 (66, 185)	1.38 (0.73, 2.06)	3476 (1873, 5137)	34.51 (18.32,
Burundi	123 (69, 197)	2.54 (1.44, 4)	3742 (2078, 6002)	68.82 (38.61,
Cabo Verde	15 (10, 20)	3.65 (2.48, 4.85)	417 (281, 561)	93.98 (63.97,
Cambodia	78 (52, 118)	0.64 (0.42, 0.96)	2232 (1481, 3338)	16.8 (11.08, 25.18)
Cameroon	170 (81, 265)	1.41 (0.68, 2.17)	4922 (2351, 7737)	36.51 (17.45,
Canada	748 (559, 940)	1.1 (0.82, 1.38)	16732 (12556,	26.27 (19.8, 32.71)
Central African Republic	34 (15, 58)	1.46 (0.63, 2.49)	1076 (463, 1892)	40.24 (17.43, 68.85)
Chad	36 (14, 62)	0.63 (0.23, 1.09)	1058 (415, 1791)	16.71 (6.42, 28.48)
Chile	280 (205, 350)	1.16 (0.85, 1.45)	5346 (4032, 6651)	22.15 (16.7, 27.51)
China	61888 (42883, 84201)	3.04 (2.11, 4.13)	1512555 (1053770, 2058982)	71.39 (49.98, 96.93)
Colombia	149 (93, 220)	0.28 (0.18, 0.42)	3738 (2368, 5429)	7.08 (4.49, 10.25)
Comoros	2 (0, 4)	0.35 (0.07, 0.77)	50 (11, 109)	9.47 (2.04, 20.85)
Congo	59 (31, 94)	2.29 (1.18, 3.66)	1712 (890, 2766)	56.88 (29.78,
Cook Islands	0 (0, 0)	0.74 (0.5, 1.03)	5 (3, 6)	18.71 (12.75,
Costa Rica	16 (10, 22)	0.3 (0.19, 0.43)	382 (246, 550)	7.31 (4.69, 10.49)
Croatia	78 (54, 106)	0.94 (0.66, 1.28)	1897 (1321, 2588)	24.81 (17.18,
Cuba	218 (147, 307)	1.16 (0.78, 1.64)	6022 (4040, 8486)	32.75 (22, 45.97)
Cyprus	8 (5, 10)	0.41 (0.28, 0.54)	176 (122, 231)	9.21 (6.47, 12.1)
Czechia	253 (186, 329)	1.28 (0.94, 1.67)	6202 (4542, 8146)	33.86 (24.73, 44.9)
C厶te d'Ivoire	133 (65, 210)	1.22 (0.59, 1.92)	3993 (1968, 6400)	31.81 (15.57, 50.4)
Democratic People's Republic of Korea	577 (382, 824)	1.74 (1.16, 2.46)	16486 (10600, 23985)	48.6 (31.58, 70.34)
Democratic Republic of the Congo	347 (141, 619)	0.95 (0.38, 1.71)	10317 (4261, 17994)	24.92 (10.13, 44.38)
Denmark	190 (143, 238)	1.67 (1.26, 2.09)	3995 (3052, 4993)	38.14 (29.13,
Djibouti	2 (0, 5)	0.34 (0.06, 0.78)	70 (13, 166)	9.27 (1.69, 21.55)
Dominica	1 (1, 1)	1.17 (0.82, 1.63)	27 (19, 39)	30.91 (21.49,
Dominican	49 (29, 74)	0.52 (0.31, 0.78)	1447 (833, 2197)	14.65 (8.52, 22.22)
Ecuador	31 (19, 46)	0.2 (0.13, 0.31)	792 (507, 1232)	5.04 (3.22, 7.82)
Egypt	22 (9, 40)	0.03 (0.01, 0.05)	720 (310, 1312)	0.92 (0.39, 1.67)
El Salvador	12 (8, 19)	0.21 (0.13, 0.32)	320 (196, 479)	5.51 (3.39, 8.25)
Equatorial Guinea	10 (5, 19)	2.27 (1.1, 4.29)	284 (137, 538)	53.7 (25.3, 101.28)

Eritrea	30 (12, 55)	1 (0.41, 1.78)	969 (397, 1769)	28.46 (11.8, 52.16)
Estonia	28 (19, 38)	1.17 (0.8, 1.6)	693 (469, 956)	31.94 (21.6, 44.13)
Eswatini	21 (12, 32)	3.47 (1.92, 5.16)	648 (350, 1011)	97.92 (53.28,
Ethiopia	145 (78, 243)	0.35 (0.19, 0.6)	4103 (2285, 6879)	8.99 (4.92, 15.07)
Fiji	3 (1, 4)	0.33 (0.16, 0.5)	73 (36, 115)	8.63 (4.17, 13.38)
Finland	89 (66, 113)	0.76 (0.58, 0.97)	1924 (1464, 2432)	18.52 (14.16,
France	2059 (1598, 2511)	1.6 (1.24, 1.94)	44701 (35013,	39.19 (30.74,
Gabon	34 (18, 50)	3.22 (1.69, 4.7)	935 (499, 1437)	80.48 (43.02,
Gambia	3 (2, 5)	0.32 (0.18, 0.48)	87 (50, 134)	8.41 (4.94, 12.92)
Georgia	25 (18, 34)	0.44 (0.31, 0.6)	685 (480, 927)	12.66 (8.95, 17.16)
Germany	2798 (2160, 3347)	1.57 (1.22, 1.88)	62612 (48621,	39.1 (30.48, 46.85)
Ghana	117 (71, 173)	0.73 (0.44, 1.07)	3294 (2010, 4876)	18.52 (11.29,
Greece	99 (74, 123)	0.44 (0.34, 0.54)	2067 (1565, 2540)	10.86 (8.19, 13.33)
Greenland	2 (1, 3)	3.27 (2.04, 4.79)	65 (40, 94)	83.15 (51.76,
Grenada	2 (2, 3)	1.84 (1.34, 2.39)	60 (44, 77)	49.19 (36.17,
Guam	1 (0, 1)	0.34 (0.04, 0.68)	19 (2, 37)	9.97 (1.26, 19.33)
Guatemala	19 (12, 30)	0.17 (0.1, 0.26)	579 (361, 875)	4.61 (2.82, 7.09)
Guinea	8 (4, 14)	0.14 (0.08, 0.25)	235 (125, 395)	3.92 (2.11, 6.56)
Guinea-Bissau	8 (4, 13)	1.01 (0.48, 1.61)	241 (116, 388)	28.19 (13.46, 45.6)
Guyana	3 (2, 5)	0.5 (0.33, 0.7)	102 (68, 143)	14.25 (9.5, 19.89)
Haiti	51 (31, 81)	0.72 (0.43, 1.12)	1513 (894, 2435)	19.05 (11.3, 30.4)
Honduras	15 (9, 22)	0.25 (0.14, 0.36)	385 (227, 574)	6.06 (3.58, 8.96)
Hungary	241 (177, 317)	1.37 (1, 1.8)	6377 (4639, 8394)	38.73 (28.24,
Iceland	7 (5, 9)	1.27 (0.92, 1.6)	158 (116, 200)	31.31 (22.98,
India	4534 (2886, 6929)	0.38 (0.24, 0.59)	133433 (85202, 202627)	10.71 (6.82, 16.32)
Indonesia	115 (18, 270)	0.05 (0.01, 0.12)	3467 (551, 7946)	1.36 (0.21, 3.15)
Iran (Islamic Republic of)	71 (44, 101)	0.1 (0.06, 0.14)	2050 (1294, 2861)	2.51 (1.57, 3.52)
Iraq	8 (4, 14)	0.03 (0.02, 0.05)	267 (134, 443)	0.94 (0.47, 1.58)
Ireland	153 (112, 192)	2.04 (1.51, 2.55)	3256 (2390, 4073)	45.33 (33.35,
Israel	22 (13, 32)	0.19 (0.11, 0.28)	493 (300, 703)	4.6 (2.79, 6.48)
Italy	818 (628, 1001)	0.6 (0.46, 0.73)	16918 (13107,	14.19 (11.09,
Jamaica	13 (9, 19)	0.46 (0.29, 0.65)	341 (222, 489)	11.52 (7.49, 16.46)
Japan	3675 (2647, 4735)	1.1 (0.8, 1.4)	72548 (52862,	25.82 (19.07,
Jordan	2 (1, 4)	0.03 (0.02, 0.06)	77 (37, 131)	0.93 (0.44, 1.57)
Kazakhstan	253 (169, 344)	1.43 (0.94, 1.96)	6912 (4660, 9304)	36.69 (24.59,
Kenya	481 (306, 751)	2.12 (1.35, 3.32)	14067 (8925,	55.5 (35.29, 86.75)
Kiribati	0 (0, 1)	0.48 (0.09, 1)	14 (3, 28)	15.08 (3.06, 30.73)
Kuwait	0 (0, 0)	0 (0, 0.01)	5 (0, 12)	0.13 (0.01, 0.33)
Kyrgyzstan	33 (23, 44)	0.71 (0.48, 0.95)	939 (653, 1250)	18.46 (12.66,
Lao People's Democratic Republic	23 (13, 37)	0.5 (0.28, 0.82)	690 (392, 1116)	13.49 (7.65, 22.01)
Latvia	46 (32, 62)	1.3 (0.89, 1.78)	1195 (818, 1635)	36.69 (25.07,
Lebanon	4 (2, 6)	0.07 (0.04, 0.11)	98 (57, 149)	1.89 (1.1, 2.87)

Lesotho	40 (22, 61)	2.91 (1.62, 4.5)	1240 (683, 1909)	85.67 (47.88,
Liberia	20 (9, 32)	0.94 (0.43, 1.47)	604 (281, 957)	24.88 (11.52,
Libya	1 (0, 2)	0.02 (0.01, 0.04)	44 (14, 69)	0.7 (0.21, 1.09)
Lithuania	76 (55, 100)	1.49 (1.07, 1.98)	2014 (1429, 2660)	42.27 (29.95,
Luxembourg	13 (9, 16)	1.3 (0.96, 1.64)	298 (221, 379)	31.68 (23.54,
Madagascar	110 (50, 195)	0.92 (0.42, 1.61)	3461 (1597, 6062)	25.5 (11.7, 44.98)
Malawi	261 (147, 404)	3.39 (1.91, 5.3)	8067 (4494, 12503)	95.71 (54.07,
Malaysia	49 (25, 81)	0.18 (0.09, 0.29)	1372 (703, 2223)	4.68 (2.38, 7.56)
Maldives	0 (0, 1)	0.08 (0.02, 0.17)	8 (2, 16)	2.15 (0.64, 4.39)
Mali	14 (8, 22)	0.17 (0.09, 0.26)	390 (223, 605)	4.17 (2.36, 6.42)
Malta	6 (4, 7)	0.65 (0.47, 0.84)	128 (94, 167)	16.25 (11.82,
Marshall Islands	0 (0, 0)	0.31 (0.14, 0.55)	4 (2, 7)	8.75 (3.99, 15.53)
Mauritania	0 (0, 0)	0 (0, 0)	0 (0, 1)	0 (0, 0.02)
Mauritius	8 (5, 12)	0.46 (0.28, 0.66)	215 (135, 315)	11.71 (7.29, 17.18)
Mexico	361 (250, 488)	0.31 (0.21, 0.42)	9653 (6661, 12929)	7.86 (5.42, 10.55)
Micronesia (Federated States of)	0 (0, 0)	0.29 (0.15, 0.48)	8 (4, 12)	8.76 (4.22, 14.27)
Monaco	1 (0, 2)	1.14 (0.01, 2.13)	22 (0, 41)	28.27 (0.25, 52.53)
Mongolia	88 (58, 130)	4.09 (2.64, 5.93)	2448 (1561, 3702)	96.51 (63.39,
Montenegro	8 (6, 11)	0.84 (0.59, 1.12)	217 (152, 293)	22.62 (15.86,
Morocco	11 (5, 19)	0.03 (0.02, 0.05)	341 (169, 574)	0.94 (0.46, 1.59)
Mozambique	109 (49, 192)	0.97 (0.43, 1.7)	3173 (1440, 5595)	25.79 (11.75,
Myanmar	149 (91, 232)	0.31 (0.19, 0.48)	4365 (2685, 6876)	8.4 (5.16, 13.19)
Namibia	10 (6, 15)	0.71 (0.45, 1.02)	308 (189, 452)	19.73 (12.21, 28.9)
Nauru	0 (0, 0)	0.6 (0.33, 0.93)	1 (1, 2)	16.75 (9.28, 26.28)
Nepal	134 (63, 230)	0.59 (0.27, 1.02)	3703 (1728, 6333)	15.42 (7.24, 26.6)
Netherlands	854 (644, 1060)	2.51 (1.9, 3.09)	18043 (13633,	57.07 (43.53,
New Zealand	105 (80, 132)	1.33 (1.02, 1.66)	2172 (1675, 2693)	29.35 (22.72,
Nicaragua	8 (5, 11)	0.18 (0.12, 0.26)	191 (127, 274)	4.1 (2.74, 5.88)
Niger	10 (3, 20)	0.12 (0.03, 0.25)	282 (76, 576)	3.16 (0.85, 6.55)
Nigeria	168 (100, 318)	0.21 (0.13, 0.38)	4463 (2644, 8818)	4.78 (2.87, 9.06)
Niue	0 (0, 0)	0.43 (0.14, 0.69)	0 (0, 0)	11.55 (3.81, 18.74)
North Macedonia	16 (11, 22)	0.48 (0.33, 0.67)	409 (277, 582)	12.56 (8.51, 17.7)
Northern Mariana Islands	0 (0, 0)	0.28 (0.01, 0.62)	5 (0, 10)	7.36 (0.4, 16.38)
Norway	68 (49, 87)	0.72 (0.51, 0.92)	1460 (1049, 1847)	16.66 (11.97, 21)
Oman	1 (0, 1)	0.04 (0.02, 0.07)	27 (12, 49)	1.02 (0.44, 1.86)
Pakistan	313 (182, 477)	0.26 (0.15, 0.39)	9987 (5785, 15220)	7.45 (4.32, 11.32)
Palau	0 (0, 0)	0.29 (0.09, 0.51)	2 (1, 4)	8.32 (2.64, 14.66)
Palestine	1 (1, 2)	0.06 (0.04, 0.08)	44 (28, 63)	1.53 (0.99, 2.21)
Panama	14 (9, 21)	0.35 (0.23, 0.5)	373 (245, 533)	8.95 (5.88, 12.8)
Papua New	8 (3, 14)	0.15 (0.06, 0.28)	247 (107, 460)	4.14 (1.74, 7.76)
Paraguay	66 (43, 92)	1.19 (0.78, 1.66)	1695 (1119, 2409)	29.41 (19.49,
Peru	78 (46, 119)	0.24 (0.14, 0.37)	2009 (1222, 3015)	6.16 (3.76, 9.27)
Philippines	258 (178, 372)	0.31 (0.21, 0.45)	7892 (5502, 11227)	8.73 (6.06, 12.47)

Poland	746 (542, 1010)	1.12 (0.82, 1.52)	19395 (14091,	30.86 (22.35,
Portugal	257 (200, 314)	1.23 (0.96, 1.5)	6378 (4947, 7753)	34.17 (26.62,
Puerto Rico	34 (23, 48)	0.51 (0.34, 0.73)	802 (528, 1138)	13.32 (8.76, 18.99)
Qatar	1 (0, 2)	0.1 (0.05, 0.17)	35 (17, 63)	2.16 (1.03, 3.87)
Republic of Korea	770 (561, 1106)	0.85 (0.62, 1.22)	17507 (12747,	19.06 (13.89,
Republic of Moldova	40 (29, 50)	0.68 (0.51, 0.86)	1133 (838, 1441)	19.94 (14.78, 25.36)
Romania	327 (238, 428)	0.98 (0.71, 1.29)	9064 (6521, 11835)	28.98 (20.68,
Russian	2549 (1862, 3332)	1.08 (0.79, 1.42)	69738 (51128,	30.49 (22.31,
Rwanda	175 (101, 291)	2.82 (1.65, 4.64)	5176 (2926, 8652)	74.35 (42.77,
Saint Kitts and Nevis	0 (0, 1)	0.58 (0.02, 1.22)	12 (1, 26)	15.67 (0.8, 33.13)
Saint Lucia	3 (2, 4)	1.23 (0.88, 1.62)	74 (53, 99)	33.17 (23.69,
Saint Vincent and the Grenadines	1 (1, 1)	0.66 (0.48, 0.86)	25 (18, 33)	17.94 (12.93, 23.29)
Samoa	0 (0, 0)	0.19 (0.1, 0.3)	8 (4, 13)	5.06 (2.74, 8.07)
San Marino	0 (0, 1)	0.51 (0.05, 0.86)	7 (1, 12)	12.53 (1.28, 21.34)
Sao Tome and Principe	1 (1, 2)	1.17 (0.59, 1.65)	34 (18, 49)	29.33 (15.2, 42.34)
Saudi Arabia	5 (1, 10)	0.02 (0, 0.04)	166 (28, 349)	0.61 (0.1, 1.25)
Senegal	10 (4, 19)	0.13 (0.05, 0.23)	336 (146, 583)	3.85 (1.64, 6.8)
Serbia	117 (81, 166)	0.78 (0.55, 1.1)	3052 (2116, 4376)	21.59 (14.97,
Seychelles	2 (1, 2)	1.52 (1.05, 2.06)	51 (36, 69)	41.78 (29.07,
Sierra Leone	31 (15, 49)	0.86 (0.42, 1.35)	876 (431, 1393)	22.23 (10.88,
Singapore	15 (9, 22)	0.18 (0.11, 0.27)	363 (221, 543)	4.39 (2.66, 6.6)
Slovakia	115 (76, 163)	1.27 (0.84, 1.79)	3090 (2015, 4380)	35.41 (23.25,
Slovenia	31 (16, 46)	0.75 (0.4, 1.14)	727 (382, 1118)	19.27 (10, 29.76)
Solomon Islands	1 (0, 2)	0.25 (0.1, 0.47)	31 (13, 58)	7.49 (3.13, 14.04)
Somalia	0 (0, 0)	0 (0, 0)	0 (0, 0)	0 (0, 0)
South Africa	1066 (773, 1443)	2.37 (1.72, 3.17)	29453 (21604,	60.87 (44.38,
South Sudan	11 (2, 26)	0.26 (0.05, 0.62)	322 (62, 792)	7.06 (1.35, 16.99)
Spain	762 (589, 946)	0.87 (0.68, 1.08)	18040 (14064,	22.65 (17.75,
Sri Lanka	188 (118, 280)	0.72 (0.46, 1.07)	5045 (3138, 7590)	18.88 (11.78,
Sudan	1 (0, 3)	0.01 (0, 0.02)	40 (2, 107)	0.18 (0.01, 0.48)
Suriname	2 (1, 3)	0.3 (0.2, 0.41)	53 (37, 74)	8.23 (5.7, 11.39)
Sweden	202 (154, 251)	0.97 (0.74, 1.19)	3972 (3077, 4876)	21.33 (16.58,
Switzerland	219 (168, 270)	1.28 (0.98, 1.57)	4522 (3474, 5518)	28.93 (22.14,
Syrian Arab Republic	3 (2, 5)	0.03 (0.01, 0.04)	97 (54, 157)	0.68 (0.38, 1.1)
Taiwan (Province of China)	822 (554, 1172)	2.11 (1.42, 3.01)	25037 (16767, 35405)	66.01 (43.98, 94.28)
Tajikistan	41 (23, 92)	0.77 (0.42, 1.73)	1287 (727, 2834)	20.49 (11.53, 45)
Thailand	844 (513, 1241)	0.8 (0.5, 1.18)	23907 (13893,	22.62 (13.19,
Timor-Leste	3 (1, 5)	0.32 (0.17, 0.55)	73 (38, 127)	8.33 (4.38, 14.54)
Togo	27 (13, 43)	0.7 (0.33, 1.12)	819 (389, 1311)	18.84 (8.92, 30.08)
Tokelau	0 (0, 0)	0.25 (0.13, 0.4)	0 (0, 0)	6.76 (3.51, 10.85)

Tonga	0 (0, 0)	0.19 (0.07, 0.35)	4 (2, 8)	5.11 (2.07, 9.51)
Trinidad and	7 (5, 10)	0.38 (0.25, 0.56)	195 (126, 282)	10.47 (6.8, 15.04)
Tunisia	8 (5, 13)	0.06 (0.04, 0.1)	230 (131, 357)	1.71 (0.98, 2.67)
Turkey	88 (53, 130)	0.1 (0.06, 0.14)	2710 (1646, 3999)	2.87 (1.75, 4.23)
Turkmenistan	70 (43, 100)	1.71 (1.08, 2.45)	2055 (1291, 2972)	46.24 (28.63, 66.4)
Tuvalu	0 (0, 0)	0.22 (0.1, 0.39)	1 (0, 1)	6.5 (3.06, 11.33)
Uganda	650 (440, 910)	4.48 (3.06, 6.26)	19488 (12835, 27651)	119.21 (80.72, 167.22)
Ukraine	687 (476, 938)	0.96 (0.67, 1.3)	20550 (14263,	29.8 (20.74, 40.77)
United Arab Emirates	35 (9, 71)	0.53 (0.13, 1.07)	1333 (346, 2700)	15.1 (3.91, 30.65)
United Kingdom	3004 (2197, 3746)	2.4 (1.77, 2.98)	60973 (45690,	53.79 (40.64,
United Republic of Tanzania	732 (431, 1187)	2.96 (1.77, 4.82)	20980 (12121, 34387)	76.81 (45.08, 125.12)
United States of America	6093 (4577, 7541)	1.11 (0.83, 1.37)	141723 (106392, 175171)	27.22 (20.51, 33.58)
United States Virgin Islands	2 (0, 3)	0.87 (0.05, 1.57)	41 (3, 73)	22.97 (1.5, 41.06)
Uruguay	101 (75, 128)	1.87 (1.39, 2.36)	2144 (1600, 2682)	43.5 (32.64, 54.4)
Uzbekistan	172 (108, 243)	0.73 (0.45, 1.03)	5456 (3507, 7678)	20.16 (12.74,
Vanuatu	0 (0, 1)	0.21 (0.11, 0.35)	12 (6, 20)	5.85 (3.06, 9.92)
Venezuela (Bolivarian Republic of)	119 (76, 174)	0.4 (0.26, 0.58)	3230 (2060, 4685)	10.52 (6.7, 15.32)
Viet Nam	957 (587, 1342)	0.98 (0.63, 1.36)	27604 (16567,	26.27 (16.07,
Yemen	11 (3, 19)	0.08 (0.02, 0.13)	359 (107, 613)	2.22 (0.65, 3.79)
Zambia	192 (106, 297)	2.71 (1.5, 4.13)	6052 (3341, 9537)	74.77 (41.01,
Zimbabwe	179 (105, 265)	2.35 (1.36, 3.54)	5718 (3448, 8516)	68.68 (40.83,

Countries and territories	Chewing tobacco			
	Deaths (95%UI)	ASMR (95%UI)	DALYs (95%UI)	ASDR (95%UI)
Afghanistan	35 (10, 62)	0.19 (0.06, 0.34)	1307 (370, 2368)	6.27 (1.81, 11.07)
Albania	0 (0, 1)	0.01 (0.01, 0.02)	10 (6, 19)	0.29 (0.15, 0.51)
Algeria	27 (14, 43)	0.08 (0.04, 0.14)	678 (339, 1099)	1.92 (0.97, 3.07)
American Samoa	0 (0, 0)	0.03 (0.02, 0.04)	0 (0, 1)	0.85 (0.47, 1.34)
Andorra	0 (0, 0)	0.01 (0.01, 0.02)	0 (0, 1)	0.34 (0.17, 0.56)
Angola	7 (3, 11)	0.06 (0.03, 0.1)	190 (96, 312)	1.5 (0.74, 2.42)
Antigua and Barbuda	0 (0, 0)	0.01 (0.01, 0.02)	0 (0, 0)	0.26 (0.15, 0.4)
Argentina	6 (4, 10)	0.01 (0.01, 0.02)	131 (81, 202)	0.25 (0.15, 0.38)
Armenia	0 (0, 0)	0 (0, 0.01)	4 (2, 7)	0.1 (0.06, 0.16)
Australia	13 (8, 21)	0.03 (0.02, 0.05)	260 (146, 411)	0.66 (0.37, 1.04)
Austria	2 (1, 3)	0.01 (0.01, 0.02)	46 (25, 73)	0.29 (0.16, 0.47)
Azerbaijan	2 (1, 4)	0.03 (0.02, 0.05)	62 (35, 108)	0.61 (0.35, 1.02)
Bahamas	0 (0, 0)	0.02 (0.01, 0.03)	2 (1, 4)	0.54 (0.28, 0.89)
Bahrain	0 (0, 0)	0.03 (0.02, 0.05)	7 (4, 13)	0.61 (0.31, 1.02)
Bangladesh	1662 (1077, 2640)	1.31 (0.85, 2.07)	41840 (26984,	30.92 (19.96,

Barbados	0 (0, 0)	0.01 (0.01, 0.02)	1 (1, 2)	0.3 (0.17, 0.46)
Belarus	1 (1, 2)	0.01 (0, 0.01)	33 (16, 57)	0.22 (0.11, 0.37)
Belgium	3 (1, 4)	0.01 (0.01, 0.02)	53 (30, 81)	0.26 (0.15, 0.41)
Belize	0 (0, 0)	0.01 (0.01, 0.01)	1 (0, 1)	0.25 (0.14, 0.39)
Benin	13 (6, 22)	0.28 (0.14, 0.47)	361 (172, 611)	6.99 (3.36, 11.62)
Bermuda	0 (0, 0)	0.02 (0.01, 0.02)	0 (0, 1)	0.38 (0.21, 0.58)
Bhutan	8 (5, 12)	1.45 (0.89, 2.21)	187 (110, 292)	32.32 (19.35,
Bolivia (Plurinational State of)	1 (0, 1)	0.01 (0, 0.01)	16 (9, 25)	0.18 (0.11, 0.28)
Bosnia and Herzegovina	1 (0, 1)	0.01 (0.01, 0.02)	14 (8, 23)	0.25 (0.14, 0.4)
Botswana	7 (3, 11)	0.5 (0.23, 0.85)	187 (87, 321)	12.1 (5.64, 20.88)
Brazil	128 (76, 198)	0.06 (0.03, 0.09)	2619 (1538, 3984)	1.11 (0.65, 1.68)
Brunei	0 (0, 0)	0.04 (0.02, 0.06)	3 (2, 4)	0.84 (0.49, 1.25)
Bulgaria	2 (1, 4)	0.02 (0.01, 0.03)	65 (34, 108)	0.55 (0.29, 0.94)
Burkina Faso	26 (14, 40)	0.29 (0.15, 0.44)	745 (403, 1144)	7.39 (3.97, 11.3)
Burundi	28 (14, 47)	0.56 (0.29, 0.93)	870 (438, 1511)	15.67 (8.08, 26.76)
Cabo Verde	3 (2, 4)	0.67 (0.4, 1.01)	68 (40, 107)	15.84 (9.35, 24.76)
Cambodia	50 (31, 76)	0.49 (0.31, 0.73)	1122 (687, 1712)	9.63 (5.92, 14.31)
Cameroon	15 (7, 26)	0.15 (0.07, 0.25)	382 (181, 669)	3.2 (1.55, 5.52)
Canada	41 (22, 65)	0.06 (0.03, 0.09)	839 (457, 1313)	1.29 (0.71, 2.04)
Central African Republic	3 (1, 5)	0.16 (0.08, 0.28)	90 (43, 154)	3.75 (1.81, 6.49)
Chad	10 (5, 17)	0.21 (0.11, 0.33)	245 (124, 399)	4.41 (2.23, 7.14)
Chile	3 (2, 5)	0.01 (0.01, 0.02)	53 (32, 80)	0.22 (0.13, 0.33)
China	1755 (910, 2842)	0.09 (0.05, 0.14)	46157 (23480,	2.17 (1.12, 3.57)
Colombia	6 (3, 9)	0.01 (0.01, 0.02)	120 (66, 191)	0.23 (0.12, 0.36)
Comoros	7 (4, 10)	1.33 (0.79, 2.09)	181 (103, 286)	35.26 (20.1, 55.76)
Congo	3 (2, 6)	0.14 (0.06, 0.25)	98 (47, 169)	3.34 (1.57, 5.61)
Cook Islands	0 (0, 0)	0.05 (0.03, 0.09)	0 (0, 1)	1.62 (0.86, 2.73)
Costa Rica	0 (0, 1)	0.01 (0, 0.01)	6 (4, 10)	0.13 (0.07, 0.2)
Croatia	1 (0, 1)	0.01 (0, 0.01)	17 (9, 28)	0.22 (0.11, 0.37)
Cuba	4 (2, 6)	0.02 (0.01, 0.03)	96 (53, 155)	0.52 (0.29, 0.84)
Cyprus	0 (0, 0)	0 (0, 0.01)	1 (1, 2)	0.08 (0.04, 0.12)
Czechia	4 (2, 6)	0.02 (0.01, 0.03)	90 (49, 144)	0.49 (0.27, 0.78)
C么te d'Ivoire	6 (3, 10)	0.07 (0.03, 0.11)	149 (74, 250)	1.38 (0.69, 2.28)
Democratic People's Republic of Korea	8 (5, 13)	0.03 (0.02, 0.04)	222 (124, 359)	0.66 (0.38, 1.08)
Democratic Republic of the Congo	69 (32, 125)	0.21 (0.09, 0.38)	1875 (880, 3343)	4.88 (2.23, 8.84)
Denmark	3 (2, 5)	0.03 (0.01, 0.04)	58 (32, 94)	0.54 (0.29, 0.85)
Djibouti	2 (1, 3)	0.27 (0.14, 0.45)	47 (24, 85)	6.68 (3.4, 11.8)
Dominica	0 (0, 0)	0.01 (0.01, 0.02)	0 (0, 0)	0.34 (0.19, 0.53)

Dominican	2 (1, 3)	0.02 (0.01, 0.03)	56 (28, 97)	0.57 (0.28, 0.98)
Ecuador	1 (0, 1)	0 (0, 0.01)	12 (6, 19)	0.08 (0.04, 0.12)
Egypt	10 (5, 17)	0.02 (0.01, 0.04)	249 (131, 405)	0.39 (0.2, 0.65)
El Salvador	1 (0, 1)	0.01 (0.01, 0.01)	12 (7, 19)	0.2 (0.11, 0.32)
Equatorial Guinea	1 (0, 1)	0.14 (0.06, 0.25)	14 (6, 26)	2.81 (1.3, 5.38)
Eritrea	10 (5, 18)	0.34 (0.17, 0.61)	328 (154, 587)	9.74 (4.62, 17.44)
Estonia	0 (0, 0)	0.01 (0.01, 0.02)	7 (4, 12)	0.33 (0.17, 0.58)
Eswatini	1 (0, 1)	0.12 (0.06, 0.21)	20 (10, 37)	3.15 (1.52, 5.59)
Ethiopia	28 (15, 48)	0.07 (0.04, 0.13)	726 (387, 1267)	1.68 (0.89, 2.9)
Fiji	0 (0, 1)	0.05 (0.02, 0.08)	13 (6, 22)	1.5 (0.7, 2.45)
Finland	1 (1, 2)	0.01 (0.01, 0.01)	22 (13, 33)	0.22 (0.12, 0.33)
France	13 (7, 20)	0.01 (0.01, 0.01)	264 (152, 400)	0.23 (0.13, 0.35)
Gabon	1 (0, 1)	0.07 (0.03, 0.12)	16 (8, 27)	1.52 (0.73, 2.45)
Gambia	0 (0, 1)	0.04 (0.03, 0.07)	9 (5, 15)	0.97 (0.57, 1.53)
Georgia	0 (0, 1)	0.01 (0, 0.01)	11 (6, 17)	0.2 (0.11, 0.32)
Germany	25 (14, 40)	0.01 (0.01, 0.02)	537 (310, 834)	0.33 (0.19, 0.51)
Ghana	12 (7, 19)	0.08 (0.04, 0.12)	300 (164, 479)	1.8 (0.99, 2.88)
Greece	1 (1, 2)	0 (0, 0.01)	18 (10, 28)	0.08 (0.05, 0.13)
Greenland	0 (0, 1)	0.45 (0.24, 0.74)	8 (4, 14)	10.94 (5.65, 17.76)
Grenada	0 (0, 0)	0.04 (0.02, 0.06)	1 (1, 2)	1 (0.54, 1.59)
Guam	0 (0, 0)	0.09 (0.05, 0.15)	6 (3, 10)	3.2 (1.8, 5.1)
Guatemala	1 (1, 2)	0.01 (0.01, 0.02)	22 (13, 34)	0.2 (0.12, 0.31)
Guinea	2 (1, 3)	0.04 (0.02, 0.06)	56 (32, 87)	0.98 (0.56, 1.53)
Guinea-Bissau	1 (0, 1)	0.13 (0.07, 0.21)	25 (12, 40)	3.19 (1.59, 5.13)
Guyana	0 (0, 0)	0.01 (0.01, 0.01)	2 (1, 3)	0.25 (0.14, 0.4)
Haiti	2 (1, 3)	0.02 (0.01, 0.04)	51 (27, 89)	0.64 (0.34, 1.1)
Honduras	1 (0, 1)	0.01 (0.01, 0.02)	19 (10, 32)	0.31 (0.15, 0.53)
Hungary	2 (1, 3)	0.01 (0.01, 0.02)	51 (27, 83)	0.31 (0.16, 0.51)
Iceland	0 (0, 1)	0.06 (0.03, 0.09)	7 (4, 11)	1.4 (0.78, 2.19)
India	9544 (6521, 14269)	0.86 (0.59, 1.29)	254057 (173925, 373840)	21.08 (14.43, 31.23)
Indonesia	184 (102, 302)	0.12 (0.06, 0.19)	3604 (2026, 5949)	1.91 (1.06, 3.13)
Iran (Islamic Republic of)	40 (23, 61)	0.06 (0.03, 0.09)	969 (555, 1464)	1.26 (0.71, 1.88)
Iraq	1 (1, 2)	0.01 (0, 0.01)	43 (24, 71)	0.16 (0.09, 0.25)
Ireland	1 (1, 2)	0.02 (0.01, 0.02)	24 (14, 36)	0.32 (0.19, 0.49)
Israel	1 (0, 1)	0.01 (0, 0.01)	12 (7, 18)	0.11 (0.06, 0.16)
Italy	8 (5, 13)	0.01 (0, 0.01)	162 (90, 245)	0.13 (0.07, 0.2)
Jamaica	0 (0, 0)	0.01 (0.01, 0.02)	7 (4, 11)	0.23 (0.13, 0.37)
Japan	137 (73, 221)	0.04 (0.02, 0.06)	2550 (1405, 4014)	0.88 (0.49, 1.36)
Jordan	1 (1, 2)	0.02 (0.01, 0.03)	29 (16, 48)	0.38 (0.21, 0.62)
Kazakhstan	12 (6, 18)	0.07 (0.04, 0.11)	275 (148, 435)	1.56 (0.84, 2.4)
Kenya	71 (41, 115)	0.34 (0.2, 0.57)	1944 (1121, 3161)	8.05 (4.65, 13.08)
Kiribati	0 (0, 0)	0.19 (0.07, 0.32)	6 (2, 10)	6.07 (1.98, 10.41)
Kuwait	0 (0, 1)	0.02 (0.01, 0.04)	13 (6, 21)	0.41 (0.21, 0.69)
Kyrgyzstan	9 (5, 14)	0.18 (0.1, 0.29)	237 (128, 377)	4.64 (2.48, 7.34)

Lao People's Democratic Republic	8 (4, 15)	0.23 (0.12, 0.4)	185 (90, 330)	4.41 (2.2, 7.81)
Latvia	0 (0, 1)	0.01 (0, 0.01)	7 (4, 13)	0.22 (0.12, 0.38)
Lebanon	1 (0, 1)	0.02 (0.01, 0.03)	21 (11, 33)	0.4 (0.22, 0.64)
Lesotho	4 (2, 6)	0.32 (0.17, 0.52)	105 (55, 169)	7.84 (4.14, 12.48)
Liberia	2 (1, 3)	0.08 (0.04, 0.14)	40 (19, 69)	1.84 (0.88, 3.16)
Libya	1 (0, 2)	0.02 (0.01, 0.03)	26 (13, 44)	0.47 (0.23, 0.8)
Lithuania	0 (0, 1)	0.01 (0.01, 0.02)	12 (7, 21)	0.26 (0.13, 0.43)
Luxembourg	0 (0, 0)	0.01 (0, 0.01)	2 (1, 3)	0.19 (0.11, 0.3)
Madagascar	170 (96, 270)	1.29 (0.73, 2.07)	5677 (3230, 8976)	39.05 (22.08,
Malawi	32 (18, 51)	0.47 (0.27, 0.76)	833 (474, 1357)	11.08 (6.35, 17.98)
Malaysia	30 (18, 48)	0.12 (0.07, 0.18)	753 (442, 1194)	2.68 (1.59, 4.27)
Maldives	1 (0, 1)	0.22 (0.14, 0.33)	15 (10, 22)	4.72 (2.96, 6.84)
Mali	11 (6, 19)	0.13 (0.07, 0.2)	332 (181, 556)	3.46 (1.91, 5.72)
Malta	0 (0, 0)	0.01 (0, 0.01)	1 (1, 2)	0.13 (0.08, 0.21)
Marshall Islands	0 (0, 0)	0.05 (0.02, 0.08)	1 (0, 1)	1.39 (0.71, 2.44)
Mauritania	2 (1, 3)	0.09 (0.05, 0.15)	42 (21, 72)	2 (1.02, 3.37)
Mauritius	2 (1, 4)	0.15 (0.09, 0.22)	51 (31, 75)	2.87 (1.76, 4.25)
Mexico	9 (6, 15)	0.01 (0, 0.01)	226 (130, 358)	0.19 (0.11, 0.3)
Micronesia (Federated States of)	0 (0, 0)	0.07 (0.03, 0.12)	2 (1, 4)	2.18 (1.04, 3.96)
Monaco	0 (0, 0)	0.02 (0.01, 0.03)	0 (0, 1)	0.4 (0.23, 0.63)
Mongolia	3 (2, 6)	0.17 (0.09, 0.28)	93 (49, 157)	3.74 (2.03, 6.26)
Montenegro	0 (0, 0)	0.01 (0.01, 0.02)	3 (2, 5)	0.32 (0.18, 0.54)
Morocco	6 (4, 10)	0.02 (0.01, 0.03)	162 (92, 271)	0.48 (0.27, 0.79)
Mozambique	29 (17, 47)	0.28 (0.16, 0.44)	792 (437, 1290)	6.88 (3.85, 10.97)
Myanmar	170 (106, 260)	0.35 (0.22, 0.55)	4822 (2983, 7349)	9.33 (5.79, 14.26)
Namibia	1 (0, 1)	0.05 (0.03, 0.08)	15 (9, 24)	1.05 (0.59, 1.67)
Nauru	0 (0, 0)	0.02 (0.01, 0.03)	0 (0, 0)	0.44 (0.23, 0.73)
Nepal	322 (212, 459)	1.52 (1, 2.16)	8122 (5258, 11598)	35.09 (22.81,
Netherlands	6 (4, 9)	0.02 (0.01, 0.03)	119 (70, 182)	0.37 (0.22, 0.57)
New Zealand	2 (1, 4)	0.03 (0.02, 0.05)	47 (26, 72)	0.63 (0.35, 0.97)
Nicaragua	0 (0, 0)	0.01 (0, 0.01)	7 (4, 11)	0.15 (0.09, 0.25)
Niger	17 (8, 29)	0.22 (0.1, 0.37)	480 (221, 838)	5.52 (2.57, 9.38)
Nigeria	6 (3, 12)	0.01 (0, 0.02)	162 (83, 329)	0.17 (0.09, 0.35)
Niue	0 (0, 0)	0.01 (0.01, 0.02)	0 (0, 0)	0.25 (0.14, 0.42)
North Macedonia	0 (0, 0)	0.01 (0, 0.01)	6 (3, 10)	0.19 (0.11, 0.33)
Northern Mariana Islands	0 (0, 0)	0.06 (0.03, 0.1)	1 (1, 2)	1.8 (0.93, 2.96)
Norway	4 (2, 7)	0.05 (0.02, 0.07)	96 (53, 150)	1.1 (0.6, 1.73)
Oman	0 (0, 1)	0.02 (0.01, 0.03)	10 (5, 17)	0.41 (0.22, 0.69)
Pakistan	1311 (854, 1895)	1.24 (0.82, 1.76)	36593 (23120,	30.08 (19.44,
Palau	0 (0, 0)	0.26 (0.16, 0.4)	2 (1, 3)	8.28 (5.05, 12.64)
Palestine	0 (0, 1)	0.02 (0.01, 0.03)	10 (6, 16)	0.37 (0.21, 0.6)

Panama	0 (0, 1)	0.01 (0, 0.01)	8 (4, 12)	0.18 (0.1, 0.3)
Papua New	2 (1, 4)	0.04 (0.02, 0.07)	81 (42, 139)	1.22 (0.65, 2.13)
Paraguay	7 (4, 11)	0.12 (0.07, 0.2)	155 (84, 257)	2.77 (1.51, 4.56)
Peru	2 (1, 2)	0 (0, 0.01)	34 (19, 54)	0.1 (0.06, 0.16)
Philippines	43 (26, 67)	0.06 (0.04, 0.1)	1065 (615, 1674)	1.32 (0.78, 2.09)
Poland	10 (5, 16)	0.01 (0.01, 0.02)	243 (132, 416)	0.39 (0.21, 0.68)
Portugal	2 (1, 3)	0.01 (0, 0.01)	39 (22, 61)	0.2 (0.11, 0.32)
Puerto Rico	1 (0, 1)	0.01 (0.01, 0.02)	14 (8, 24)	0.22 (0.12, 0.38)
Qatar	0 (0, 0)	0.02 (0.01, 0.04)	4 (2, 7)	0.34 (0.17, 0.6)
Republic of Korea	22 (11, 39)	0.02 (0.01, 0.04)	491 (253, 873)	0.53 (0.28, 0.95)
Republic of Moldova	0 (0, 1)	0.01 (0, 0.01)	9 (5, 15)	0.17 (0.09, 0.27)
Romania	5 (3, 8)	0.02 (0.01, 0.03)	142 (75, 250)	0.46 (0.24, 0.82)
Russian	23 (13, 36)	0.01 (0.01, 0.01)	580 (320, 925)	0.25 (0.14, 0.4)
Rwanda	18 (9, 31)	0.32 (0.16, 0.56)	493 (235, 883)	7.55 (3.73, 13.29)
Saint Kitts and Nevis	0 (0, 0)	0.01 (0.01, 0.02)	0 (0, 0)	0.26 (0.15, 0.42)
Saint Lucia	0 (0, 0)	0.02 (0.01, 0.03)	1 (1, 2)	0.52 (0.28, 0.82)
Saint Vincent and the Grenadines	0 (0, 0)	0.01 (0, 0.01)	0 (0, 0)	0.16 (0.09, 0.26)
Samoa	0 (0, 0)	0.01 (0.01, 0.02)	1 (0, 1)	0.32 (0.18, 0.51)
San Marino	0 (0, 0)	0.01 (0, 0.01)	0 (0, 0)	0.13 (0.06, 0.23)
Sao Tome and Principe	0 (0, 0)	0.06 (0.03, 0.09)	1 (1, 2)	1.25 (0.61, 1.97)
Saudi Arabia	3 (1, 4)	0.01 (0.01, 0.02)	82 (44, 138)	0.33 (0.19, 0.54)
Senegal	3 (2, 6)	0.05 (0.03, 0.09)	77 (40, 120)	1.05 (0.54, 1.66)
Serbia	2 (1, 3)	0.01 (0.01, 0.02)	45 (24, 74)	0.32 (0.17, 0.53)
Seychelles	0 (0, 0)	0.13 (0.08, 0.2)	3 (2, 4)	2.36 (1.46, 3.5)
Sierra Leone	5 (2, 7)	0.14 (0.07, 0.22)	118 (57, 192)	3.2 (1.55, 5.16)
Singapore	1 (1, 2)	0.02 (0.01, 0.03)	30 (16, 47)	0.37 (0.2, 0.57)
Slovakia	1 (0, 2)	0.01 (0.01, 0.02)	24 (12, 40)	0.27 (0.14, 0.46)
Slovenia	0 (0, 1)	0.01 (0.01, 0.02)	11 (6, 20)	0.3 (0.15, 0.53)
Solomon Islands	0 (0, 0)	0.06 (0.03, 0.1)	8 (3, 14)	1.75 (0.88, 3.07)
Somalia	19 (10, 31)	0.29 (0.15, 0.48)	567 (300, 968)	7.53 (3.97, 12.75)
South Africa	92 (51, 151)	0.24 (0.13, 0.4)	1895 (1115, 2985)	4.37 (2.51, 7.01)
South Sudan	9 (5, 15)	0.23 (0.13, 0.38)	248 (131, 424)	5.73 (3.1, 9.76)
Spain	6 (3, 9)	0.01 (0, 0.01)	124 (71, 191)	0.15 (0.09, 0.23)
Sri Lanka	217 (139, 322)	0.88 (0.55, 1.3)	5106 (3148, 7627)	19.4 (12.27, 29)
Sudan	32 (8, 58)	0.17 (0.04, 0.31)	923 (247, 1742)	4.33 (1.14, 8.1)
Suriname	0 (0, 0)	0.01 (0, 0.01)	1 (1, 1)	0.14 (0.08, 0.22)
Sweden	8 (4, 13)	0.04 (0.02, 0.07)	193 (103, 299)	1.15 (0.62, 1.8)
Switzerland	2 (1, 3)	0.01 (0.01, 0.02)	43 (24, 67)	0.27 (0.15, 0.43)
Syrian Arab Republic	0 (0, 1)	0 (0, 0.01)	11 (6, 18)	0.08 (0.05, 0.13)
Taiwan (Province of China)	23 (12, 40)	0.06 (0.03, 0.1)	720 (373, 1286)	1.94 (0.99, 3.44)

Tajikistan	16 (8, 34)	0.31 (0.15, 0.68)	473 (238, 1004)	7.84 (3.92, 16.66)
Thailand	159 (89, 249)	0.16 (0.09, 0.25)	3164 (1756, 4990)	3.06 (1.72, 4.84)
Timor-Leste	1 (1, 2)	0.13 (0.08, 0.22)	21 (12, 34)	2.57 (1.5, 4.27)
Togo	3 (2, 6)	0.1 (0.05, 0.17)	91 (46, 150)	2.35 (1.19, 3.8)
Tokelau	0 (0, 0)	0.04 (0.02, 0.06)	0 (0, 0)	1.08 (0.58, 1.75)
Tonga	0 (0, 0)	0.04 (0.02, 0.07)	1 (1, 2)	1.24 (0.68, 2.03)
Trinidad and	0 (0, 0)	0.01 (0, 0.01)	2 (1, 4)	0.13 (0.07, 0.22)
Tunisia	8 (4, 13)	0.06 (0.03, 0.11)	178 (92, 297)	1.38 (0.72, 2.29)
Turkey	6 (3, 9)	0.01 (0, 0.01)	160 (91, 253)	0.17 (0.1, 0.27)
Turkmenistan	1 (1, 2)	0.04 (0.02, 0.06)	38 (22, 61)	0.9 (0.53, 1.39)
Tuvalu	0 (0, 0)	0.05 (0.03, 0.08)	0 (0, 0)	1.52 (0.81, 2.55)
Uganda	37 (22, 58)	0.29 (0.17, 0.46)	966 (577, 1521)	6.58 (3.93, 10.27)
Ukraine	7 (4, 12)	0.01 (0.01, 0.02)	219 (107, 368)	0.32 (0.16, 0.54)
United Arab Emirates	7 (2, 16)	0.13 (0.04, 0.28)	284 (70, 614)	3.39 (0.89, 6.9)
United Kingdom	25 (15, 39)	0.02 (0.01, 0.03)	482 (288, 729)	0.42 (0.25, 0.63)
United Republic of Tanzania	51 (28, 82)	0.22 (0.12, 0.36)	1340 (736, 2171)	5.21 (2.85, 8.39)
United States of America	934 (512, 1436)	0.17 (0.09, 0.26)	20751 (11483, 31804)	3.96 (2.22, 6.05)
United States Virgin Islands	0 (0, 0)	0.01 (0.01, 0.02)	1 (0, 1)	0.32 (0.18, 0.51)
Uruguay	1 (1, 1)	0.02 (0.01, 0.02)	17 (10, 26)	0.35 (0.21, 0.52)
Uzbekistan	49 (26, 80)	0.2 (0.11, 0.34)	1566 (829, 2549)	5.73 (3.09, 9.28)
Vanuatu	0 (0, 0)	0.06 (0.03, 0.09)	4 (2, 6)	1.73 (0.9, 3.02)
Venezuela (Bolivarian Republic of)	14 (7, 23)	0.05 (0.02, 0.08)	360 (185, 608)	1.18 (0.61, 1.99)
Viet Nam	58 (33, 90)	0.07 (0.04, 0.1)	1298 (709, 2084)	1.38 (0.78, 2.17)
Yemen	64 (19, 109)	0.51 (0.15, 0.85)	1774 (538, 3020)	12.2 (3.71, 20.85)
Zambia	18 (10, 29)	0.31 (0.17, 0.49)	485 (268, 798)	7.03 (3.93, 11.38)
Zimbabwe	11 (6, 19)	0.21 (0.11, 0.38)	254 (147, 405)	3.91 (2.2, 6.53)

Countries and territories	High BMI			
	Deaths (95%UI)	ASMR (95%UI)	DALYs (95%UI)	ASDR (95%UI)
Afghanistan	182 (40, 387)	1.5 (0.34, 3.11)	5453 (1147, 12069)	37.49 (8.26, 78.96)
Albania	17 (5, 33)	0.41 (0.12, 0.78)	397 (121, 775)	9.61 (2.88, 18.73)
Algeria	108 (36, 193)	0.35 (0.11, 0.61)	2700 (883, 4831)	7.67 (2.51, 13.69)
American Samoa	0 (0, 0)	0.52 (0.18, 0.92)	7 (2, 11)	12.91 (4.43, 22.28)
Andorra	1 (0, 3)	0.98 (0.23, 1.93)	35 (8, 68)	24.78 (5.88, 48.21)
Angola	133 (34, 280)	1.16 (0.29, 2.49)	3970 (1048, 8382)	30.06 (7.84, 63.72)
Antigua and Barbuda	1 (0, 1)	0.73 (0.25, 1.34)	19 (7, 34)	17.28 (6.05, 31.47)
Argentina	707 (240, 1278)	1.31 (0.45, 2.36)	15549 (5038,	29.55 (9.6, 53.16)
Armenia	21 (7, 35)	0.49 (0.18, 0.84)	485 (180, 818)	11.54 (4.27, 19.47)
Australia	539 (192, 931)	1.28 (0.45, 2.22)	11135 (3830,	28.69 (9.78, 49.18)
Austria	110 (34, 198)	0.65 (0.2, 1.18)	2524 (766, 4610)	16.28 (4.85, 29.97)

Azerbaijan	243 (78, 469)	2.71 (0.88, 5.29)	6622 (2203, 12498)	63.74 (20.71,
Bahamas	6 (2, 11)	1.48 (0.44, 2.77)	170 (50, 314)	39.39 (11.54,
Bahrain	6 (2, 10)	0.71 (0.24, 1.23)	164 (53, 277)	14.51 (4.87, 24.84)
Bangladesh	495 (130, 1148)	0.37 (0.1, 0.86)	14332 (3968,	10.24 (2.8, 23.74)
Barbados	7 (2, 13)	1.4 (0.47, 2.55)	161 (53, 295)	33.75 (11.1, 61.42)
Belarus	143 (39, 278)	0.9 (0.25, 1.74)	3913 (997, 7644)	25.2 (6.47, 48.73)
Belgium	247 (83, 451)	1.12 (0.37, 2.06)	5357 (1724, 9874)	27.09 (8.65, 49.91)
Belize	2 (1, 4)	0.74 (0.24, 1.33)	59 (18, 104)	19.23 (6.02, 34.13)
Benin	53 (15, 108)	1.06 (0.3, 2.18)	1539 (437, 3129)	28.16 (7.98, 57.38)
Bermuda	2 (1, 3)	1.41 (0.46, 2.48)	41 (13, 73)	33.76 (10.16,
Bhutan	5 (1, 10)	0.81 (0.23, 1.72)	123 (36, 263)	20.57 (6.08, 43.72)
Bolivia (Plurinational State of)	58 (19, 107)	0.68 (0.23, 1.26)	1393 (470, 2559)	15.26 (5.13, 28.1)
Bosnia and Herzegovina	37 (12, 67)	0.62 (0.2, 1.13)	909 (281, 1649)	15.49 (4.79, 28.18)
Botswana	49 (14, 93)	3.53 (1.05, 6.69)	1425 (415, 2813)	90.57 (26.12,
Brazil	3727 (1254, 6419)	1.56 (0.52, 2.69)	98571 (32630, 169601)	40.1 (13.34, 68.99)
Brunei	1 (0, 3)	0.47 (0.14, 0.97)	33 (9, 67)	10.07 (2.87, 20.76)
Bulgaria	83 (25, 151)	0.63 (0.19, 1.14)	2151 (644, 3915)	17.64 (5.26, 31.95)
Burkina Faso	62 (17, 136)	0.7 (0.19, 1.47)	1745 (494, 3778)	17.33 (4.83, 37.54)
Burundi	49 (11, 119)	1.03 (0.23, 2.56)	1462 (331, 3571)	27.24 (6.27, 66.42)
Cabo Verde	16 (4, 30)	3.76 (1.06, 7.12)	424 (112, 818)	95.28 (25.47,
Cambodia	33 (9, 74)	0.28 (0.07, 0.63)	935 (252, 2046)	7.13 (1.88, 15.77)
Cameroon	187 (51, 373)	1.6 (0.45, 3.16)	5289 (1513, 10463)	39.85 (11.02,
Canada	768 (255, 1327)	1.12 (0.37, 1.92)	16753 (5563,	26.01 (8.6, 44.57)
Central African Republic	20 (4, 51)	0.89 (0.18, 2.25)	635 (139, 1592)	24.09 (5.12, 60.57)
Chad	27 (6, 62)	0.49 (0.11, 1.12)	771 (183, 1736)	12.51 (2.96, 28.43)
Chile	278 (92, 483)	1.15 (0.38, 2)	5152 (1763, 8955)	21.34 (7.32, 37.16)
China	36181 (9426, 79606)	1.8 (0.47, 3.95)	859654 (221497, 1875382)	40.79 (10.61, 88.68)
Colombia	263 (84, 504)	0.49 (0.16, 0.95)	5644 (1830, 10906)	10.69 (3.48, 20.64)
Comoros	9 (3, 20)	1.86 (0.51, 4.11)	255 (71, 557)	48.68 (13.32,
Congo	61 (18, 118)	2.3 (0.69, 4.51)	1794 (529, 3485)	58.82 (17.25,
Cook Islands	0 (0, 0)	0.92 (0.27, 1.61)	5 (2, 10)	22.28 (6.33, 39.4)
Costa Rica	25 (8, 47)	0.49 (0.15, 0.93)	549 (169, 1024)	10.61 (3.26, 19.77)
Croatia	75 (23, 135)	0.9 (0.27, 1.63)	1783 (520, 3267)	23.1 (6.61, 42.47)
Cuba	257 (77, 474)	1.36 (0.41, 2.51)	6681 (1966, 12265)	36.09 (10.63,
Cyprus	5 (2, 10)	0.27 (0.08, 0.51)	111 (32, 209)	5.79 (1.72, 10.95)
Czechia	223 (73, 397)	1.12 (0.37, 1.99)	5364 (1714, 9557)	28.99 (8.93, 51.7)
C么te d'Ivoire	107 (28, 218)	0.98 (0.25, 2.01)	3208 (844, 6524)	25.57 (6.6, 52.29)
Democratic People's Republic of Korea	178 (28, 507)	0.55 (0.09, 1.56)	4651 (708, 13407)	13.93 (2.17, 40.28)

Democratic Republic of the Congo	383 (97, 866)	1.08 (0.28, 2.48)	11080 (2779, 24253)	27.39 (6.93, 61.66)
Denmark	123 (38, 227)	1.08 (0.33, 2)	2599 (782, 4774)	24.95 (7.58, 45.79)
Djibouti	11 (3, 24)	1.71 (0.41, 3.92)	322 (78, 750)	44.41 (11.04,
Dominica	1 (0, 3)	1.52 (0.5, 2.79)	33 (10, 60)	36.74 (11.83,
Dominican	55 (16, 113)	0.59 (0.17, 1.2)	1527 (438, 3083)	15.62 (4.5, 31.69)
Ecuador	71 (23, 133)	0.49 (0.16, 0.91)	1625 (527, 3061)	10.58 (3.43, 19.96)
Egypt	347 (117, 630)	0.53 (0.18, 0.98)	10261 (3461,	13.9 (4.73, 25.19)
El Salvador	26 (9, 49)	0.44 (0.15, 0.82)	578 (194, 1069)	9.89 (3.31, 18.23)
Equatorial Guinea	11 (3, 23)	2.55 (0.79, 5.23)	312 (95, 636)	59.45 (18.14,
Eritrea	43 (9, 99)	1.72 (0.35, 4.01)	1242 (270, 2924)	42.01 (8.84, 95.62)
Estonia	24 (8, 44)	1.01 (0.32, 1.84)	584 (178, 1075)	26.42 (7.66, 49.01)
Eswatini	30 (10, 57)	5.3 (1.67, 9.7)	878 (275, 1690)	137.95 (42.48, 261.49)
Ethiopia	123 (31, 280)	0.3 (0.07, 0.69)	3523 (904, 7831)	7.6 (1.94, 17.19)
Fiji	6 (2, 11)	0.87 (0.27, 1.65)	168 (55, 317)	20.62 (6.64, 38.77)
Finland	78 (25, 141)	0.64 (0.21, 1.16)	1570 (485, 2834)	14.61 (4.48, 26.5)
France	1283 (404, 2343)	0.99 (0.31, 1.8)	27603 (8612,	24.1 (7.45, 43.86)
Gabon	31 (9, 58)	2.92 (0.86, 5.54)	857 (241, 1621)	73.44 (21, 141.99)
Gambia	4 (1, 7)	0.43 (0.13, 0.79)	116 (36, 215)	11.24 (3.45, 20.44)
Georgia	30 (11, 53)	0.51 (0.18, 0.91)	749 (252, 1347)	13.58 (4.55, 24.32)
Germany	1874 (594, 3352)	1.05 (0.33, 1.87)	41761 (12855,	26.05 (7.92, 46.37)
Ghana	143 (50, 257)	0.92 (0.32, 1.65)	3893 (1318, 7039)	22.34 (7.69, 39.95)
Greece	84 (28, 151)	0.36 (0.11, 0.64)	1656 (514, 2970)	8.45 (2.6, 15.14)
Greenland	3 (1, 5)	3.94 (1.23, 7.11)	74 (25, 136)	96.22 (31.03,
Grenada	2 (0, 3)	1.56 (0.44, 2.92)	49 (13, 91)	40.27 (10.99,
Guam	2 (0, 3)	0.82 (0.23, 1.52)	42 (11, 76)	21.72 (5.72, 39.49)
Guatemala	46 (14, 90)	0.43 (0.13, 0.85)	1133 (368, 2236)	9.82 (3.16, 19.28)
Guinea	16 (4, 32)	0.29 (0.08, 0.6)	431 (126, 885)	7.3 (2.14, 15.03)
Guinea-Bissau	6 (2, 13)	0.82 (0.2, 1.8)	181 (45, 386)	21.88 (5.47, 46.98)
Guyana	3 (1, 7)	0.53 (0.17, 1.01)	100 (32, 192)	14.16 (4.59, 27.28)
Haiti	29 (7, 67)	0.41 (0.1, 0.97)	825 (210, 1892)	10.58 (2.68, 24.33)
Honduras	23 (7, 45)	0.39 (0.11, 0.78)	553 (159, 1095)	8.93 (2.57, 17.75)
Hungary	231 (74, 407)	1.31 (0.41, 2.3)	6009 (1904, 10573)	36.31 (11.35,
Iceland	6 (2, 11)	1.12 (0.35, 2.04)	138 (43, 251)	27.03 (8.36, 49.12)
India	5895 (1788, 11558)	0.51 (0.16, 1.01)	165616 (50780, 325517)	13.5 (4.16, 26.55)
Indonesia	759 (222, 1562)	0.34 (0.1, 0.7)	22248 (6703,	8.88 (2.61, 18.19)
Iran (Islamic Republic of)	841 (303, 1438)	1.23 (0.43, 2.1)	19865 (7056, 33971)	26.36 (9.41, 45.2)
Iraq	94 (32, 168)	0.42 (0.15, 0.74)	2652 (930, 4652)	10.23 (3.55, 18.07)
Ireland	132 (45, 233)	1.75 (0.59, 3.09)	2727 (897, 4787)	37.74 (12.42,
Israel	47 (16, 84)	0.4 (0.14, 0.71)	908 (306, 1627)	8.19 (2.76, 14.73)
Italy	629 (200, 1145)	0.44 (0.14, 0.8)	12321 (3918,	10.07 (3.17, 18.26)
Jamaica	22 (8, 41)	0.74 (0.26, 1.37)	523 (181, 951)	17.68 (6.12, 32.24)

Japan	1527 (327, 3565)	0.45 (0.09, 1.03)	29227 (5868,	10.19 (2, 23.53)
Jordan	28 (10, 46)	0.45 (0.16, 0.75)	762 (269, 1260)	10.38 (3.73, 17.31)
Kazakhstan	424 (136, 720)	2.55 (0.8, 4.34)	10574 (3624,	58.46 (19.22,
Kenya	591 (183, 1175)	2.65 (0.78, 5.34)	17312 (5513,	68.15 (21.08,
Kiribati	1 (0, 3)	1.7 (0.42, 3.59)	42 (10, 86)	48.87 (12.04,
Kuwait	13 (5, 22)	0.58 (0.21, 0.97)	334 (128, 546)	11.64 (4.38, 19.15)
Kyrgyzstan	46 (14, 83)	1.06 (0.34, 1.91)	1174 (373, 2138)	24.5 (7.7, 44.29)
Lao People's Democratic Republic	15 (4, 31)	0.33 (0.09, 0.7)	441 (119, 914)	8.76 (2.35, 18.23)
Latvia	39 (12, 73)	1.09 (0.33, 2.03)	983 (284, 1829)	29.62 (8.21, 55.19)
Lebanon	21 (7, 36)	0.41 (0.14, 0.7)	489 (167, 855)	9.43 (3.22, 16.5)
Lesotho	45 (13, 87)	3.59 (1.05, 6.72)	1297 (383, 2487)	93.57 (27.2,
Liberia	23 (7, 46)	1.09 (0.32, 2.15)	716 (215, 1395)	29.55 (8.85, 58.05)
Libya	25 (8, 44)	0.49 (0.16, 0.86)	679 (225, 1232)	12.02 (4.07, 21.68)
Lithuania	62 (19, 110)	1.19 (0.35, 2.13)	1581 (446, 2846)	32.52 (8.92, 59.12)
Luxembourg	8 (3, 16)	0.85 (0.27, 1.58)	193 (61, 358)	20.44 (6.37, 37.83)
Madagascar	149 (37, 324)	1.31 (0.32, 2.88)	4500 (1140, 9799)	34.48 (8.63, 75.31)
Malawi	286 (84, 614)	3.93 (1.17, 8.32)	8261 (2362, 17535)	102 (30.11, 216.85)
Malaysia	154 (50, 291)	0.59 (0.19, 1.11)	3891 (1268, 7425)	13.79 (4.53, 26.15)
Maldives	1 (0, 2)	0.27 (0.07, 0.55)	23 (7, 45)	6.57 (1.92, 12.87)
Mali	35 (10, 71)	0.4 (0.11, 0.82)	989 (286, 2061)	10.36 (2.94, 21.49)
Malta	4 (1, 8)	0.5 (0.15, 0.95)	96 (30, 185)	11.94 (3.61, 22.92)
Marshall Islands	0 (0, 0)	0.54 (0.15, 1.15)	6 (2, 13)	14.48 (3.99, 30.65)
Mauritania	23 (7, 45)	1.18 (0.35, 2.24)	586 (181, 1142)	27.11 (8.37, 52.49)
Mauritius	10 (3, 19)	0.56 (0.19, 1.07)	245 (82, 466)	13.56 (4.59, 25.74)
Mexico	507 (171, 901)	0.44 (0.15, 0.78)	12563 (4233,	10.38 (3.5, 18.33)
Micronesia (Federated States of)	1 (0, 1)	0.79 (0.23, 1.52)	19 (6, 36)	21.83 (6.48, 41.96)
Monaco	1 (0, 3)	1.6 (0.52, 2.81)	30 (10, 52)	37.14 (12.2, 66.08)
Mongolia	106 (33, 200)	5.52 (1.71, 10.69)	2678 (877, 5067)	115.51 (36.49, 217.44)
Montenegro	8 (3, 15)	0.86 (0.27, 1.54)	220 (68, 391)	22.89 (7.14, 40.46)
Morocco	143 (45, 255)	0.47 (0.15, 0.83)	3757 (1171, 6753)	11.18 (3.5, 19.97)
Mozambique	106 (29, 227)	0.97 (0.27, 2.08)	3032 (789, 6466)	24.92 (6.8, 53.49)
Myanmar	128 (36, 279)	0.27 (0.08, 0.6)	3600 (1022, 7848)	7.06 (1.98, 15.31)
Namibia	9 (3, 17)	0.63 (0.2, 1.22)	252 (79, 497)	16.6 (5.04, 32.16)
Nauru	0 (0, 0)	0.84 (0.23, 1.65)	1 (0, 3)	22.27 (6.01, 43.27)
Nepal	138 (37, 290)	0.62 (0.16, 1.29)	3855 (1060, 7963)	16.06 (4.35, 33.28)
Netherlands	567 (183, 1056)	1.66 (0.53, 3.09)	11878 (3737,	37.51 (11.73,
New Zealand	92 (32, 159)	1.16 (0.4, 2)	1886 (637, 3226)	25.36 (8.6, 43.72)
Nicaragua	13 (4, 26)	0.31 (0.1, 0.63)	298 (92, 601)	6.6 (2.08, 13.32)
Niger	34 (8, 79)	0.45 (0.1, 1.03)	958 (219, 2178)	11.11 (2.51, 25.43)
Nigeria	147 (42, 343)	0.18 (0.05, 0.41)	3977 (1152, 9483)	4.18 (1.21, 9.76)
Niue	0 (0, 0)	0.76 (0.25, 1.4)	0 (0, 1)	18.93 (5.91, 34.88)

North Macedonia	16 (5, 30)	0.51 (0.17, 0.93)	418 (132, 767)	12.87 (4.08, 23.42)
Northern Mariana Islands	1 (0, 1)	0.99 (0.3, 1.77)	14 (4, 25)	23.49 (6.82, 41.65)
Norway	51 (16, 96)	0.53 (0.16, 1)	1059 (318, 1990)	11.98 (3.56, 22.61)
Oman	13 (4, 22)	0.82 (0.3, 1.41)	363 (125, 657)	18.13 (6.35, 31.48)
Pakistan	1582 (486, 3092)	1.42 (0.43, 2.77)	46221 (14320,	36.73 (11.31,
Palau	0 (0, 0)	0.77 (0.24, 1.38)	5 (1, 9)	19.74 (6.18, 34.75)
Palestine	7 (2, 12)	0.29 (0.09, 0.54)	185 (61, 343)	6.95 (2.23, 12.96)
Panama	17 (5, 33)	0.4 (0.12, 0.81)	396 (119, 796)	9.53 (2.86, 19.16)
Papua New	11 (3, 27)	0.23 (0.06, 0.56)	363 (95, 828)	6.21 (1.59, 14.43)
Paraguay	58 (17, 111)	1.05 (0.31, 2.03)	1447 (408, 2849)	25.25 (7.17, 49.3)
Peru	116 (38, 223)	0.36 (0.12, 0.7)	2582 (829, 4894)	8.01 (2.59, 15.17)
Philippines	168 (48, 336)	0.21 (0.06, 0.43)	4880 (1400, 9786)	5.56 (1.61, 11.05)
Poland	773 (271, 1328)	1.14 (0.4, 1.96)	18843 (6475,	29.43 (10.05,
Portugal	165 (47, 310)	0.77 (0.21, 1.47)	3927 (1045, 7550)	20.72 (5.3, 40.23)
Puerto Rico	62 (20, 112)	0.89 (0.29, 1.59)	1336 (437, 2406)	21.36 (6.84, 38.31)
Qatar	9 (3, 17)	2 (0.67, 3.56)	283 (92, 528)	32.19 (10.79,
Republic of Korea	320 (65, 704)	0.35 (0.07, 0.77)	7221 (1380, 16158)	7.85 (1.54, 17.55)
Republic of Moldova	32 (9, 59)	0.55 (0.16, 1.01)	902 (250, 1678)	15.72 (4.34, 29.22)
Romania	293 (88, 523)	0.87 (0.26, 1.55)	7894 (2335, 14111)	24.96 (7.19, 44.43)
Russian Federation	2457 (830, 4225)	1.04 (0.35, 1.78)	62822 (20788, 109476)	27.16 (8.91, 47.54)
Rwanda	92 (22, 200)	1.53 (0.37, 3.32)	2594 (619, 5757)	38.4 (9.09, 84)
Saint Kitts and Nevis	1 (0, 2)	1.2 (0.36, 2.22)	23 (7, 43)	30.21 (9.47, 56.72)
Saint Lucia	2 (1, 5)	1.13 (0.35, 2.18)	65 (19, 125)	29.02 (8.65, 55.83)
Saint Vincent and the Grenadines	1 (0, 2)	0.62 (0.18, 1.18)	23 (6, 43)	16.2 (4.65, 30.82)
Samoa	1 (0, 1)	0.47 (0.15, 0.85)	19 (6, 35)	12.21 (3.94, 21.91)
San Marino	0 (0, 1)	0.42 (0.11, 0.86)	6 (1, 12)	10.2 (2.48, 21.31)
Sao Tome and Principe	1 (0, 2)	1.2 (0.35, 2.34)	35 (10, 66)	29.83 (8.79, 57.97)
Saudi Arabia	117 (43, 197)	0.69 (0.25, 1.14)	3646 (1341, 6257)	16.06 (5.88, 26.89)
Senegal	63 (18, 124)	0.87 (0.24, 1.7)	1676 (469, 3373)	21 (5.98, 41.89)
Serbia	130 (42, 231)	0.85 (0.27, 1.52)	3246 (1013, 5784)	22.45 (6.86, 39.58)
Seychelles	1 (0, 3)	1.15 (0.33, 2.24)	40 (11, 75)	32.1 (9.21, 61.58)
Sierra Leone	20 (5, 44)	0.56 (0.13, 1.27)	572 (146, 1235)	14.57 (3.66, 31.84)
Singapore	25 (6, 50)	0.31 (0.09, 0.64)	564 (144, 1124)	6.93 (1.75, 13.89)
Slovakia	99 (29, 184)	1.09 (0.32, 2.03)	2602 (733, 4844)	29.59 (8.23, 55.4)
Slovenia	36 (12, 66)	0.88 (0.27, 1.62)	840 (252, 1539)	22.15 (6.55, 40.53)
Solomon Islands	2 (1, 5)	0.63 (0.17, 1.35)	75 (21, 159)	18.41 (5.04, 38.7)
Somalia	46 (6, 140)	0.67 (0.09, 2.08)	1443 (199, 4333)	18.13 (2.47, 55.16)
South Africa	1456 (528, 2494)	3.37 (1.2, 5.72)	37482 (13859,	79.47 (29.16,
South Sudan	75 (21, 164)	1.94 (0.54, 4.15)	2183 (601, 4752)	49.43 (13.9,
Spain	646 (200, 1167)	0.72 (0.21, 1.31)	14582 (4156,	18 (5, 32.91)

Sri Lanka	192 (53, 390)	0.76 (0.21, 1.58)	4682 (1286, 9591)	17.75 (4.84, 35.9)
Sudan	229 (56, 465)	1.29 (0.3, 2.58)	6163 (1466, 12595)	30.41 (7.4, 61.15)
Suriname	2 (1, 4)	0.37 (0.13, 0.66)	60 (20, 110)	9.43 (3.1, 17.08)
Sweden	144 (48, 259)	0.69 (0.22, 1.24)	2818 (905, 5081)	15.13 (4.81, 27.5)
Switzerland	134 (41, 249)	0.78 (0.23, 1.43)	2748 (816, 5111)	17.57 (5.2, 32.59)
Syrian Arab Republic	33 (11, 62)	0.28 (0.09, 0.52)	896 (279, 1660)	6.6 (2.09, 12.27)
Taiwan (Province of China)	581 (117, 1266)	1.49 (0.3, 3.25)	17107 (3358, 37725)	45.14 (8.71, 98.8)
Tajikistan	53 (14, 137)	1.11 (0.29, 2.97)	1536 (406, 3749)	26.74 (7.09, 69.06)
Thailand	577 (165, 1180)	0.55 (0.16, 1.13)	16011 (4500,	15.15 (4.22, 30.79)
Timor-Leste	1 (0, 3)	0.15 (0.03, 0.39)	35 (8, 86)	3.99 (0.87, 9.9)
Togo	28 (8, 58)	0.77 (0.21, 1.59)	810 (233, 1672)	19.43 (5.42, 39.92)
Tokelau	0 (0, 0)	0.51 (0.16, 0.98)	0 (0, 0)	12.55 (4.12, 24.44)
Tonga	0 (0, 1)	0.61 (0.2, 1.16)	13 (4, 24)	15.8 (5.02, 29.33)
Trinidad and	9 (3, 17)	0.48 (0.16, 0.92)	230 (73, 437)	12.31 (3.95, 23.57)
Tunisia	35 (12, 66)	0.28 (0.09, 0.53)	853 (277, 1609)	6.53 (2.14, 12.23)
Turkey	460 (155, 807)	0.52 (0.18, 0.91)	11888 (4173,	12.94 (4.51, 22.45)
Turkmenistan	125 (40, 220)	3.2 (0.99, 5.61)	3496 (1132, 6211)	81.22 (25.94,
Tuvalu	0 (0, 0)	0.52 (0.15, 1.04)	1 (0, 3)	13.74 (4.25, 26.96)
Uganda	349 (103, 690)	2.45 (0.69, 4.89)	10158 (2980,	63.78 (18.9,
Ukraine	623 (182, 1161)	0.85 (0.24, 1.59)	17747 (4945,	25.22 (6.98, 47.86)
United Arab Emirates	160 (31, 322)	3.53 (0.7, 7.23)	5906 (1166, 11770)	85.07 (16.55, 175.17)
United Kingdom	2932 (996, 5023)	2.31 (0.78, 3.96)	57443 (19086,	49.86 (16.59,
United Republic of Tanzania	562 (164, 1174)	2.29 (0.66, 4.75)	16009 (4721, 33238)	58.73 (17.31, 121.68)
United States of America	7428 (2540, 12221)	1.34 (0.46, 2.2)	169400 (56319, 276895)	32.19 (10.75, 52.6)
United States Virgin Islands	3 (1, 5)	1.5 (0.5, 2.56)	66 (22, 115)	37.14 (12.44, 65.08)
Uruguay	86 (29, 156)	1.59 (0.54, 2.86)	1824 (610, 3269)	37.02 (12.11,
Uzbekistan	377 (121, 661)	1.87 (0.59, 3.29)	11079 (3538,	45.5 (14.46, 80.14)
Vanuatu	1 (0, 2)	0.53 (0.14, 1.14)	27 (8, 58)	14.12 (3.97, 30.59)
Venezuela (Bolivarian Republic of)	149 (47, 289)	0.52 (0.16, 1)	3699 (1112, 7135)	12.26 (3.73, 23.65)
Viet Nam	266 (56, 619)	0.27 (0.06, 0.63)	7581 (1558, 17693)	7.24 (1.48, 16.89)
Yemen	88 (18, 203)	0.67 (0.13, 1.54)	2512 (499, 5761)	16.87 (3.35, 38.96)
Zambia	180 (55, 356)	2.6 (0.78, 5.09)	5484 (1668, 10841)	70.02 (21.39,
Zimbabwe	187 (52, 373)	2.65 (0.72, 5.35)	5446 (1555, 10880)	69.46 (19.55,

**Countries and territories**	**Diet low in fruits**			
	**Deaths (95%UI)**	**ASMR (95%UI)**	**DALYs (95%UI)**	**ASDR (95%UI)**
Afghanistan	145 (32, 295)	1.19 (0.28, 2.4)	4425 (967, 9151)	29.26 (6.45, 60.05)
Albania	1 (0, 4)	0.03 (0, 0.11)	23 (3, 99)	0.61 (0.07, 2.56)
Algeria	15 (1, 51)	0.05 (0, 0.17)	344 (23, 1208)	1 (0.07, 3.5)

American Samoa	0 (0, 0)	0.14 (0.02, 0.35)	2 (0, 4)	3.07 (0.53, 7.87)
Andorra	0 (0, 1)	0.17 (0.01, 0.54)	6 (0, 19)	4.25 (0.36, 13.79)
Angola	98 (18, 256)	0.95 (0.18, 2.39)	2865 (518, 7407)	22.41 (4.15, 58.1)
Antigua and Barbuda	0 (0, 1)	0.14 (0.01, 0.51)	3 (0, 12)	3.18 (0.21, 11.38)
Argentina	81 (7, 312)	0.15 (0.01, 0.57)	1685 (146, 6545)	3.21 (0.28, 12.37)
Armenia	2 (0, 8)	0.05 (0, 0.19)	47 (4, 179)	1.17 (0.09, 4.39)
Australia	162 (24, 428)	0.38 (0.06, 1)	3179 (498, 8347)	8.16 (1.29, 21.25)
Austria	14 (1, 55)	0.08 (0.01, 0.33)	314 (22, 1253)	2.06 (0.14, 8.08)
Azerbaijan	37 (2, 140)	0.45 (0.03, 1.68)	1006 (64, 3822)	10.01 (0.65, 37.51)
Bahamas	2 (0, 4)	0.4 (0.06, 1.08)	43 (7, 121)	10.15 (1.56, 28.08)
Bahrain	0 (0, 1)	0.04 (0, 0.15)	10 (1, 36)	0.86 (0.09, 3.11)
Bangladesh	1190 (433, 2070)	0.93 (0.34, 1.63)	30895 (11362,	22.62 (8.28, 39.93)
Barbados	4 (1, 7)	0.76 (0.21, 1.49)	83 (22, 164)	17.41 (4.72, 34.46)
Belarus	48 (7, 130)	0.3 (0.05, 0.82)	1297 (183, 3474)	8.5 (1.25, 22.67)
Belgium	69 (7, 202)	0.31 (0.03, 0.91)	1421 (125, 4290)	7.19 (0.63, 21.55)
Belize	0 (0, 0)	0.01 (0, 0.06)	1 (0, 5)	0.37 (0.12, 1.59)
Benin	46 (15, 84)	0.98 (0.32, 1.81)	1230 (410, 2261)	23.33 (7.63, 42.91)
Bermuda	1 (0, 1)	0.46 (0.1, 1.11)	13 (3, 31)	10.78 (2.3, 25.37)
Bhutan	4 (1, 9)	0.81 (0.25, 1.59)	111 (33, 221)	18.81 (5.71, 37.35)
Bolivia (Plurinational State of)	17 (2, 48)	0.2 (0.02, 0.58)	380 (42, 1084)	4.22 (0.46, 12.12)
Bosnia and Herzegovina	8 (1, 25)	0.13 (0.01, 0.43)	181 (14, 608)	3.18 (0.26, 10.46)
Botswana	44 (17, 74)	3.27 (1.3, 5.39)	1273 (497, 2154)	81.51 (31.9,
Brazil	508 (60, 1641)	0.22 (0.03, 0.7)	13219 (1530,	5.4 (0.63, 17.46)
Brunei	1 (0, 2)	0.48 (0.14, 0.92)	32 (10, 61)	9.93 (3.02, 19.08)
Bulgaria	30 (5, 73)	0.23 (0.04, 0.56)	767 (139, 1872)	6.4 (1.25, 15.36)
Burkina Faso	127 (60, 199)	1.45 (0.7, 2.27)	3427 (1608, 5374)	34.68 (16.43,
Burundi	17 (1, 66)	0.37 (0.02, 1.43)	507 (31, 2016)	9.25 (0.58, 37.09)
Cabo Verde	11 (3, 20)	2.56 (0.75, 4.82)	268 (82, 504)	60.99 (18.4, 114.8)
Cambodia	76 (30, 124)	0.66 (0.26, 1.09)	2012 (801, 3340)	15.75 (6.33, 25.91)
Cameroon	38 (3, 132)	0.34 (0.03, 1.14)	1091 (85, 3638)	8.11 (0.62, 27.58)
Canada	170 (17, 508)	0.25 (0.02, 0.74)	3485 (344, 10531)	5.46 (0.53, 16.41)
Central African Republic	41 (11, 81)	1.91 (0.57, 3.75)	1265 (357, 2471)	49.12 (13.85, 96.53)
Chad	69 (30, 111)	1.29 (0.57, 2.06)	1862 (806, 2981)	30.98 (13.48,
Chile	81 (11, 225)	0.34 (0.04, 0.93)	1416 (178, 4024)	5.89 (0.76, 16.67)
China	19549 (3277, 51906)	1.02 (0.18, 2.68)	436575 (71099, 1152946)	21.28 (3.6, 55.83)
Colombia	51 (4, 180)	0.1 (0.01, 0.34)	1023 (74, 3653)	1.94 (0.14, 6.94)
Comoros	4 (0, 13)	0.8 (0.06, 2.66)	104 (8, 338)	19.83 (1.46, 65.2)
Congo	35 (8, 78)	1.42 (0.32, 3.21)	1018 (225, 2249)	34.04 (7.51, 75.67)
Cook Islands	0 (0, 0)	0.17 (0.02, 0.53)	1 (0, 3)	3.93 (0.39, 12.25)
Costa Rica	4 (0, 13)	0.07 (0, 0.26)	79 (5, 298)	1.54 (0.11, 5.79)

Croatia	12 (1, 41)	0.15 (0.01, 0.49)	282 (19, 955)	3.72 (0.26, 12.68)
Cuba	39 (3, 139)	0.2 (0.02, 0.73)	959 (72, 3406)	5.23 (0.4, 18.58)
Cyprus	1 (0, 4)	0.07 (0, 0.23)	28 (2, 93)	1.48 (0.11, 4.92)
Czechia	69 (13, 169)	0.35 (0.07, 0.85)	1650 (328, 4078)	9 (1.8, 21.94)
C么te d'Ivoire	49 (7, 140)	0.48 (0.06, 1.38)	1413 (194, 4047)	11.51 (1.53, 33.27)
Democratic People's Republic of Korea	295 (46, 775)	0.92 (0.14, 2.39)	7728 (1228, 20391)	23.16 (3.67, 60.58)
Democratic Republic of the Congo	515 (154, 990)	1.51 (0.45, 2.96)	14659 (4412, 28214)	36.68 (10.9, 70.42)
Denmark	22 (2, 76)	0.19 (0.02, 0.66)	421 (33, 1484)	4.03 (0.32, 14.21)
Djibouti	18 (7, 33)	3.1 (1.28, 5.52)	528 (206, 991)	75.35 (30.04,
Dominica	0 (0, 0)	0.01 (0.01, 0.04)	0 (0, 1)	0.32 (0.2, 0.71)
Dominican	2 (0, 10)	0.03 (0, 0.11)	65 (10, 285)	0.66 (0.11, 2.88)
Ecuador	4 (0, 17)	0.03 (0, 0.12)	94 (10, 380)	0.61 (0.07, 2.42)
Egypt	20 (2, 87)	0.03 (0, 0.13)	634 (55, 2621)	0.84 (0.08, 3.55)
El Salvador	10 (1, 26)	0.16 (0.02, 0.43)	208 (32, 544)	3.54 (0.54, 9.25)
Equatorial Guinea	2 (0, 7)	0.47 (0.04, 1.49)	57 (6, 183)	10.64 (1.04, 34.21)
Eritrea	59 (17, 120)	2.23 (0.62, 4.57)	1837 (552, 3642)	56.96 (16.38,
Estonia	8 (1, 19)	0.33 (0.06, 0.8)	190 (34, 459)	8.65 (1.58, 20.6)
Eswatini	14 (4, 29)	2.46 (0.65, 5.05)	413 (109, 874)	63.8 (16.89,
Ethiopia	310 (143, 503)	0.79 (0.37, 1.28)	8238 (3760, 13365)	18.59 (8.57, 30.31)
Fiji	4 (2, 7)	0.63 (0.23, 1.08)	109 (40, 192)	13.8 (5.11, 24.26)
Finland	33 (6, 79)	0.27 (0.05, 0.64)	645 (120, 1540)	6.02 (1.14, 14.24)
France	541 (96, 1355)	0.41 (0.07, 1.02)	11454 (2089,	10.1 (1.89, 24.57)
Gabon	3 (0, 10)	0.25 (0.02, 0.98)	72 (6, 283)	6.1 (0.51, 25.02)
Gambia	6 (3, 9)	0.64 (0.31, 0.96)	156 (77, 241)	15.5 (7.52, 23.54)
Georgia	13 (3, 30)	0.23 (0.05, 0.52)	331 (71, 746)	6.07 (1.4, 13.41)
Germany	541 (64, 1575)	0.3 (0.04, 0.87)	11869 (1382,	7.45 (0.9, 21.41)
Ghana	13 (1, 49)	0.09 (0.01, 0.32)	357 (35, 1365)	2.03 (0.21, 7.61)
Greece	5 (1, 20)	0.02 (0, 0.08)	91 (13, 383)	0.47 (0.07, 2)
Greenland	1 (0, 2)	0.77 (0.08, 2.37)	14 (1, 44)	18.73 (1.98, 57.46)
Grenada	0 (0, 1)	0.26 (0.02, 0.97)	8 (0, 28)	6.45 (0.42, 23.92)
Guam	0 (0, 1)	0.14 (0.01, 0.44)	7 (1, 22)	3.5 (0.38, 11.24)
Guatemala	23 (4, 61)	0.22 (0.04, 0.57)	562 (97, 1417)	4.8 (0.81, 12.32)
Guinea	7 (1, 23)	0.14 (0.01, 0.44)	203 (22, 627)	3.41 (0.34, 10.69)
Guinea-Bissau	7 (2, 15)	0.97 (0.23, 2.08)	199 (51, 424)	24.29 (5.95, 52.46)
Guyana	2 (0, 4)	0.3 (0.07, 0.64)	53 (13, 117)	7.7 (1.89, 16.84)
Haiti	25 (4, 69)	0.37 (0.06, 1)	707 (117, 1906)	9.1 (1.47, 25.03)
Honduras	8 (1, 25)	0.15 (0.02, 0.44)	202 (23, 595)	3.26 (0.37, 9.75)
Hungary	67 (11, 170)	0.38 (0.06, 0.95)	1733 (282, 4360)	10.57 (1.69, 26.16)
Iceland	2 (0, 5)	0.36 (0.05, 0.96)	43 (6, 115)	8.55 (1.29, 22.58)
India	10795 (4977, 17115)	0.96 (0.44, 1.53)	292416 (135588, 459962)	24.11 (11.15, 38.01)
Indonesia	629 (159, 1377)	0.31 (0.08, 0.69)	16749 (4262,	7.16 (1.83, 15.52)

Iran (Islamic Republic of)	55 (8, 185)	0.08 (0.01, 0.27)	1317 (189, 4446)	1.7 (0.25, 5.75)
Iraq	29 (5, 73)	0.13 (0.02, 0.32)	842 (140, 2112)	3.16 (0.51, 8.01)
Ireland	36 (4, 106)	0.48 (0.05, 1.39)	705 (74, 2097)	9.75 (1.04, 29.04)
Israel	3 (0, 13)	0.03 (0, 0.11)	58 (7, 241)	0.53 (0.07, 2.22)
Italy	73 (9, 241)	0.05 (0.01, 0.17)	1368 (164, 4638)	1.13 (0.13, 3.88)
Jamaica	5 (0, 16)	0.16 (0.01, 0.54)	112 (10, 362)	3.79 (0.33, 12.38)
Japan	1620 (412, 3526)	0.49 (0.13, 1.02)	31488 (8596,	11.63 (3.47, 23.02)
Jordan	6 (1, 17)	0.09 (0.01, 0.27)	162 (20, 457)	2.16 (0.25, 6.13)
Kazakhstan	135 (24, 337)	0.85 (0.15, 2.08)	3262 (562, 8244)	18.4 (3.31, 46.07)
Kenya	409 (122, 825)	1.95 (0.59, 3.96)	11550 (3490,	46.56 (13.86,
Kiribati	1 (0, 1)	0.94 (0.15, 2.36)	19 (3, 47)	23.14 (3.76, 58.38)
Kuwait	2 (0, 6)	0.09 (0.01, 0.3)	54 (5, 163)	1.8 (0.14, 5.63)
Kyrgyzstan	28 (8, 56)	0.68 (0.19, 1.33)	702 (195, 1391)	14.96 (4.18, 29.61)
Lao People's Democratic Republic	9 (1, 26)	0.22 (0.03, 0.63)	250 (32, 729)	5.11 (0.64, 14.91)
Latvia	19 (5, 38)	0.53 (0.15, 1.03)	480 (136, 929)	14.52 (4.14, 28.15)
Lebanon	1 (0, 4)	0.02 (0, 0.08)	22 (3, 93)	0.42 (0.05, 1.75)
Lesotho	49 (20, 81)	3.88 (1.61, 6.39)	1389 (569, 2358)	100.3 (40.61,
Liberia	17 (5, 34)	0.87 (0.26, 1.74)	474 (139, 941)	20.66 (6.04, 41.45)
Libya	3 (0, 13)	0.07 (0, 0.25)	94 (6, 350)	1.6 (0.1, 6.2)
Lithuania	23 (4, 53)	0.44 (0.09, 1.01)	577 (115, 1339)	12.11 (2.57, 27.73)
Luxembourg	3 (0, 7)	0.28 (0.04, 0.73)	63 (10, 164)	6.77 (1.06, 17.48)
Madagascar	190 (59, 376)	1.75 (0.54, 3.44)	5777 (1809, 11510)	44.25 (13.79,
Malawi	237 (51, 524)	3.29 (0.72, 7.27)	6883 (1553, 15492)	83.93 (18.04,
Malaysia	48 (5, 148)	0.2 (0.02, 0.6)	1165 (115, 3613)	4.25 (0.45, 13.08)
Maldives	1 (0, 2)	0.26 (0.06, 0.55)	18 (5, 39)	5.45 (1.3, 11.73)
Mali	40 (12, 74)	0.47 (0.15, 0.87)	1099 (345, 2099)	11.66 (3.62, 21.93)
Malta	1 (0, 4)	0.16 (0.02, 0.47)	31 (3, 90)	3.93 (0.42, 11.33)
Marshall Islands	0 (0, 0)	0.41 (0.12, 0.87)	4 (1, 9)	9.91 (2.79, 21.57)
Mauritania	23 (10, 38)	1.18 (0.51, 1.9)	568 (248, 940)	26.45 (11.44,
Mauritius	8 (3, 15)	0.49 (0.17, 0.86)	204 (71, 360)	11.41 (3.99, 20.07)
Mexico	90 (12, 267)	0.08 (0.01, 0.23)	2200 (307, 6510)	1.82 (0.25, 5.41)
Micronesia (Federated States of)	0 (0, 1)	0.47 (0.13, 0.97)	9 (2, 19)	11.48 (2.98, 24.17)
Monaco	0 (0, 0)	0.07 (0.01, 0.32)	1 (0, 6)	1.78 (0.23, 7.48)
Mongolia	120 (56, 188)	6.59 (2.96, 10.4)	2913 (1367, 4609)	130.44 (61.94, 202.53)
Montenegro	0 (0, 2)	0.04 (0.01, 0.16)	9 (1, 37)	1.02 (0.13, 4.08)
Morocco	10 (1, 41)	0.04 (0, 0.14)	264 (26, 1039)	0.8 (0.08, 3.17)
Mozambique	193 (73, 320)	1.87 (0.7, 3.1)	5283 (1984, 8741)	44.85 (16.86,
Myanmar	199 (66, 364)	0.44 (0.15, 0.81)	5285 (1792, 9570)	10.67 (3.62, 19.54)
Namibia	9 (4, 14)	0.62 (0.25, 1.01)	241 (98, 397)	15.86 (6.44, 26.06)
Nauru	0 (0, 0)	0.34 (0.06, 0.84)	0 (0, 1)	8.36 (1.5, 20.67)

Nepal	169 (47, 334)	0.8 (0.22, 1.56)	4387 (1248, 8611)	18.79 (5.32, 36.64)
Netherlands	150 (14, 465)	0.44 (0.04, 1.36)	3017 (267, 9382)	9.62 (0.87, 29.97)
New Zealand	19 (2, 59)	0.24 (0.02, 0.74)	378 (37, 1163)	5.15 (0.52, 15.68)
Nicaragua	12 (5, 19)	0.29 (0.11, 0.48)	263 (103, 442)	5.9 (2.32, 9.89)
Niger	71 (27, 122)	0.99 (0.38, 1.65)	1907 (722, 3265)	22.86 (8.67, 38.98)
Nigeria	94 (23, 222)	0.12 (0.03, 0.28)	2455 (614, 5988)	2.64 (0.65, 6.33)
Niue	0 (0, 0)	0.24 (0.04, 0.62)	0 (0, 0)	5.59 (1.05, 14.31)
North Macedonia	2 (0, 8)	0.07 (0, 0.24)	53 (3, 194)	1.7 (0.11, 6.04)
Northern Mariana Islands	0 (0, 0)	0.2 (0.02, 0.58)	3 (0, 8)	4.47 (0.5, 12.97)
Norway	14 (2, 39)	0.14 (0.02, 0.4)	274 (40, 770)	3.09 (0.45, 8.68)
Oman	1 (0, 3)	0.05 (0.01, 0.17)	21 (2, 77)	0.94 (0.11, 3.64)
Pakistan	1639 (591, 2944)	1.49 (0.52, 2.66)	48274 (17356,	38.02 (13.67,
Palau	0 (0, 0)	0.22 (0.03, 0.6)	1 (0, 3)	5.37 (0.85, 14.26)
Palestine	2 (0, 6)	0.09 (0.01, 0.27)	55 (8, 151)	2.11 (0.28, 5.9)
Panama	6 (1, 17)	0.15 (0.02, 0.42)	148 (24, 404)	3.57 (0.57, 9.76)
Papua New	13 (3, 27)	0.29 (0.07, 0.62)	371 (98, 769)	6.82 (1.78, 14.47)
Paraguay	10 (1, 36)	0.18 (0.01, 0.67)	242 (15, 870)	4.22 (0.26, 15.25)
Peru	33 (4, 99)	0.1 (0.01, 0.31)	670 (75, 2074)	2.06 (0.22, 6.41)
Philippines	75 (13, 202)	0.1 (0.02, 0.26)	2189 (414, 5852)	2.5 (0.46, 6.67)
Poland	254 (58, 580)	0.38 (0.09, 0.85)	6065 (1383, 13835)	9.61 (2.19, 21.76)
Portugal	33 (2, 115)	0.15 (0.01, 0.53)	777 (48, 2749)	4.16 (0.26, 14.49)
Puerto Rico	9 (1, 31)	0.12 (0.01, 0.42)	171 (12, 620)	2.64 (0.18, 9.58)
Qatar	0 (0, 1)	0.05 (0.01, 0.19)	8 (1, 33)	0.75 (0.14, 3.15)
Republic of Korea	168 (21, 495)	0.19 (0.02, 0.55)	3402 (383, 10272)	3.75 (0.44, 11.28)
Republic of Moldova	13 (2, 29)	0.22 (0.04, 0.51)	349 (67, 818)	6.2 (1.22, 14.4)
Romania	59 (5, 190)	0.18 (0.01, 0.55)	1596 (127, 5036)	5.12 (0.41, 16.07)
Russian	883 (193, 1958)	0.38 (0.08, 0.83)	22577 (5100,	9.95 (2.32, 21.82)
Rwanda	2 (1, 2)	0.03 (0.02, 0.04)	43 (27, 70)	0.63 (0.41, 0.99)
Saint Kitts and Nevis	1 (0, 1)	0.84 (0.3, 1.44)	15 (5, 26)	20.2 (7.25, 35.1)
Saint Lucia	1 (0, 2)	0.29 (0.03, 0.91)	17 (2, 51)	7.47 (0.77, 22.8)
Saint Vincent and the Grenadines	0 (0, 0)	0.09 (0.01, 0.34)	3 (0, 12)	2.26 (0.15, 8.4)
Samoa	0 (0, 1)	0.21 (0.05, 0.45)	8 (2, 17)	5.01 (1.25, 10.87)
San Marino	0 (0, 0)	0.08 (0.01, 0.26)	1 (0, 3)	1.84 (0.14, 6.37)
Sao Tome and Principe	0 (0, 1)	0.14 (0.01, 0.55)	4 (0, 15)	3.03 (0.24, 12.56)
Saudi Arabia	20 (2, 62)	0.12 (0.01, 0.36)	657 (66, 1960)	2.71 (0.25, 8.36)
Senegal	78 (28, 129)	1.09 (0.4, 1.79)	2005 (714, 3384)	25.38 (9.12, 42.4)
Serbia	12 (1, 46)	0.08 (0.01, 0.31)	292 (20, 1130)	2.13 (0.15, 8.27)
Seychelles	1 (0, 1)	0.53 (0.09, 1.34)	16 (3, 40)	13.42 (2.46, 33.69)
Sierra Leone	29 (9, 55)	0.86 (0.26, 1.61)	791 (248, 1481)	20.5 (6.37, 38.58)
Singapore	5 (0, 18)	0.06 (0, 0.25)	99 (7, 393)	1.25 (0.09, 4.94)
Slovakia	35 (7, 82)	0.39 (0.08, 0.91)	917 (180, 2142)	10.61 (2.18, 24.79)

Slovenia	5 (0, 16)	0.11 (0.01, 0.39)	106 (7, 361)	2.85 (0.2, 9.67)
Solomon Islands	2 (0, 3)	0.53 (0.14, 1.07)	52 (13, 110)	13.7 (3.53, 28.47)
Somalia	209 (80, 358)	3.19 (1.22, 5.42)	6326 (2458, 10854)	82.21 (31.54,
South Africa	945 (367, 1523)	2.21 (0.87, 3.57)	24145 (9332,	51.23 (19.7, 82.97)
South Sudan	35 (5, 99)	0.93 (0.12, 2.67)	1012 (139, 2879)	22.88 (3.1, 64.53)
Spain	58 (5, 222)	0.06 (0.01, 0.24)	1270 (118, 4844)	1.58 (0.15, 5.86)
Sri Lanka	204 (68, 385)	0.82 (0.27, 1.54)	4950 (1620, 9419)	18.9 (6.19, 35.69)
Sudan	83 (10, 239)	0.47 (0.06, 1.36)	2229 (275, 6207)	10.89 (1.28, 31.07)
Suriname	1 (0, 2)	0.12 (0.01, 0.33)	18 (2, 51)	2.87 (0.36, 8)
Sweden	41 (6, 119)	0.19 (0.03, 0.54)	762 (98, 2203)	4.05 (0.51, 11.64)
Switzerland	38 (3, 117)	0.22 (0.02, 0.67)	763 (64, 2360)	4.89 (0.42, 15.1)
Syrian Arab Republic	5 (0, 19)	0.05 (0, 0.17)	148 (8, 525)	1.11 (0.06, 3.92)
Taiwan (Province of China)	103 (8, 394)	0.27 (0.02, 1.01)	3074 (215, 12014)	8.37 (0.57, 32.41)
Tajikistan	56 (16, 132)	1.26 (0.35, 3.13)	1598 (473, 3687)	28.77 (8.28, 67.86)
Thailand	119 (8, 460)	0.12 (0.01, 0.44)	3132 (210, 12392)	3.04 (0.2, 11.89)
Timor-Leste	5 (2, 8)	0.6 (0.25, 1.03)	119 (48, 207)	14.05 (5.68, 24.35)
Togo	48 (21, 77)	1.35 (0.59, 2.16)	1345 (579, 2226)	32.78 (14.28,
Tokelau	0 (0, 0)	0.23 (0.05, 0.54)	0 (0, 0)	5.24 (1.15, 12.32)
Tonga	0 (0, 0)	0.28 (0.07, 0.64)	5 (1, 12)	6.44 (1.53, 14.61)
Trinidad and	4 (1, 9)	0.22 (0.05, 0.48)	100 (25, 222)	5.44 (1.34, 11.9)
Tunisia	4 (0, 17)	0.03 (0, 0.14)	98 (6, 395)	0.76 (0.05, 3.06)
Turkey	11 (2, 46)	0.01 (0, 0.05)	311 (62, 1232)	0.34 (0.07, 1.34)
Turkmenistan	23 (2, 76)	0.61 (0.05, 1.99)	658 (52, 2113)	15.31 (1.16, 49.4)
Tuvalu	0 (0, 0)	0.36 (0.1, 0.75)	1 (0, 2)	8.44 (2.42, 17.64)
Uganda	43 (5, 164)	0.3 (0.03, 1.19)	1304 (133, 5032)	7.68 (0.84, 29.73)
Ukraine	323 (90, 636)	0.45 (0.13, 0.87)	9242 (2597, 18205)	13.32 (3.83, 26.35)
United Arab Emirates	14 (1, 55)	0.27 (0.01, 1.19)	552 (21, 2206)	6.53 (0.26, 28.72)
United Kingdom	1210 (340, 2518)	0.94 (0.26, 1.95)	22902 (6463,	19.81 (5.68, 40.91)
United Republic of Tanzania	269 (40, 731)	1.13 (0.18, 3.03)	7549 (1081, 20564)	27.49 (3.95, 74.78)
United States of America	1653 (256, 4522)	0.3 (0.05, 0.81)	36663 (5776, 99780)	7.06 (1.14, 18.88)
United States Virgin Islands	0 (0, 1)	0.12 (0.01, 0.43)	5 (1, 19)	3.09 (0.32, 11.04)
Uruguay	23 (2, 69)	0.42 (0.04, 1.26)	461 (39, 1423)	9.25 (0.77, 28.71)
Uzbekistan	98 (9, 299)	0.53 (0.05, 1.6)	2918 (287, 8762)	12.31 (1.19, 37.01)
Vanuatu	0 (0, 1)	0.24 (0.03, 0.7)	11 (1, 32)	5.85 (0.73, 17.18)
Venezuela (Bolivarian Republic of)	36 (3, 120)	0.13 (0.01, 0.42)	866 (70, 2860)	2.9 (0.24, 9.53)
Viet Nam	288 (45, 735)	0.3 (0.05, 0.77)	8051 (1262, 20662)	7.76 (1.21, 19.82)
Yemen	98 (22, 195)	0.78 (0.18, 1.61)	2699 (624, 5409)	18.35 (4.18, 36.37)

Zambia	270 (121, 431)	4.1 (1.87, 6.49)	7952 (3514, 12890)	103.62 (46.22, 166.27)
Zimbabwe	289 (135, 447)	4.26 (1.97, 6.55)	8251 (3755, 12696)	106.56 (49.34, 164.62)

Countries and territories	Diet low in vegetables			
	Deaths (95%UI)	ASMR (95%UI)	DALYs (95%UI)	ASDR (95%UI)
Afghanistan	92 (13, 197)	0.77 (0.11, 1.63)	2743 (396, 5886)	18.74 (2.65, 40.36)
Albania	0 (0, 1)	0.01 (0, 0.02)	5 (2, 10)	0.11 (0.05, 0.25)
Algeria	18 (1, 43)	0.06 (0, 0.14)	422 (19, 1005)	1.24 (0.06, 2.93)
American Samoa	0 (0, 0)	0.15 (0.02, 0.28)	2 (0, 3)	3.21 (0.53, 6.13)
Andorra	0 (0, 1)	0.15 (0.01, 0.37)	5 (0, 12)	3.49 (0.19, 8.86)
Angola	80 (9, 169)	0.79 (0.09, 1.65)	2271 (242, 4815)	18.27 (2.11, 38.65)
Antigua and Barbuda	0 (0, 1)	0.27 (0.03, 0.53)	6 (1, 12)	5.81 (0.57, 11.7)
Argentina	185 (8, 392)	0.34 (0.01, 0.72)	3866 (163, 8232)	7.3 (0.3, 15.55)
Armenia	0 (0, 0)	0 (0, 0.01)	3 (3, 4)	0.08 (0.06, 0.11)
Australia	107 (5, 229)	0.25 (0.01, 0.53)	2030 (84, 4374)	5.11 (0.21, 11)
Austria	24 (1, 52)	0.14 (0.01, 0.3)	514 (21, 1118)	3.27 (0.14, 7.11)
Azerbaijan	3 (1, 9)	0.05 (0.02, 0.15)	72 (36, 176)	0.85 (0.36, 2.19)
Bahamas	1 (0, 3)	0.33 (0.01, 0.71)	34 (1, 73)	7.98 (0.34, 17.3)
Bahrain	0 (0, 1)	0.04 (0, 0.11)	7 (1, 19)	0.72 (0.08, 2.03)
Bangladesh	528 (91, 1118)	0.42 (0.07, 0.87)	13594 (2300,	9.98 (1.7, 20.96)
Barbados	2 (0, 4)	0.41 (0.03, 0.85)	44 (3, 92)	9.18 (0.65, 19.2)
Belarus	6 (1, 20)	0.04 (0.01, 0.13)	149 (23, 478)	0.96 (0.15, 3.13)
Belgium	37 (2, 88)	0.16 (0.01, 0.38)	695 (45, 1739)	3.36 (0.22, 8.55)
Belize	1 (0, 1)	0.23 (0.03, 0.45)	17 (2, 33)	5.66 (0.8, 11.11)
Benin	20 (2, 42)	0.43 (0.04, 0.91)	529 (41, 1127)	10.16 (0.79, 21.75)
Bermuda	0 (0, 1)	0.26 (0.01, 0.57)	7 (0, 16)	5.63 (0.24, 12.6)
Bhutan	2 (0, 5)	0.44 (0.05, 0.92)	58 (6, 125)	9.95 (1.09, 21.37)
Bolivia (Plurinational State of)	21 (3, 42)	0.25 (0.04, 0.52)	457 (74, 926)	5.15 (0.83, 10.45)
Bosnia and Herzegovina	3 (0, 7)	0.04 (0, 0.12)	56 (6, 162)	0.96 (0.09, 2.78)
Botswana	17 (3, 35)	1.3 (0.2, 2.6)	499 (75, 1015)	32.19 (4.88, 64.68)
Brazil	1045 (94, 2097)	0.44 (0.04, 0.88)	26669 (2384,	10.9 (0.98, 21.84)
Brunei	1 (0, 1)	0.26 (0.03, 0.53)	16 (1, 33)	5.23 (0.5, 10.62)
Bulgaria	2 (0, 6)	0.02 (0, 0.04)	41 (12, 121)	0.31 (0.1, 0.88)
Burkina Faso	48 (7, 93)	0.55 (0.08, 1.07)	1286 (199, 2530)	13.04 (2, 25.58)
Burundi	58 (10, 120)	1.3 (0.23, 2.65)	1680 (286, 3517)	32.28 (5.63, 67.04)
Cabo Verde	4 (0, 8)	0.91 (0.05, 1.99)	89 (4, 201)	20.42 (0.94, 46.02)
Cambodia	36 (6, 70)	0.31 (0.05, 0.63)	943 (151, 1840)	7.42 (1.2, 14.45)
Cameroon	46 (2, 106)	0.41 (0.02, 0.93)	1235 (53, 2914)	9.58 (0.45, 22.54)
Canada	142 (6, 310)	0.2 (0.01, 0.44)	2901 (122, 6331)	4.45 (0.19, 9.78)
Central African Republic	27 (5, 55)	1.28 (0.23, 2.54)	828 (138, 1682)	32.63 (5.74, 65.78)

Chad	30 (5, 59)	0.57 (0.09, 1.11)	810 (132, 1591)	13.53 (2.2, 26.68)
Chile	48 (2, 109)	0.2 (0.01, 0.45)	797 (38, 1807)	3.31 (0.16, 7.51)
China	1049 (479, 2347)	0.06 (0.02, 0.14)	20176 (10483,	1.03 (0.51, 2.24)
Colombia	91 (9, 188)	0.17 (0.02, 0.35)	1821 (178, 3799)	3.46 (0.34, 7.22)
Comoros	6 (1, 13)	1.36 (0.26, 2.64)	168 (33, 328)	32.87 (6.45, 63.67)
Congo	29 (5, 57)	1.19 (0.19, 2.36)	822 (133, 1608)	28.16 (4.45, 54.71)
Cook Islands	0 (0, 0)	0.24 (0.03, 0.48)	1 (0, 3)	5.41 (0.69, 10.92)
Costa Rica	8 (1, 17)	0.17 (0.02, 0.34)	174 (22, 351)	3.37 (0.44, 6.82)
Croatia	13 (1, 30)	0.16 (0.01, 0.36)	304 (13, 717)	3.9 (0.16, 9.26)
Cuba	45 (2, 106)	0.24 (0.01, 0.55)	1057 (51, 2507)	5.68 (0.28, 13.52)
Cyprus	2 (0, 4)	0.09 (0, 0.19)	33 (1, 74)	1.73 (0.08, 3.88)
Czechia	42 (2, 93)	0.21 (0.01, 0.47)	990 (44, 2210)	5.31 (0.23, 11.95)
C么te d'Ivoire	51 (7, 106)	0.51 (0.07, 1.04)	1422 (200, 2968)	12.04 (1.7, 25.31)
Democratic People's Republic of Korea	162 (7, 366)	0.5 (0.02, 1.14)	4095 (171, 9386)	12.32 (0.51, 28.22)
Democratic Republic of the Congo	339 (56, 706)	1.01 (0.17, 2.08)	9489 (1514, 19563)	24.08 (3.96, 50.17)
Denmark	26 (1, 59)	0.23 (0.01, 0.51)	511 (24, 1160)	4.75 (0.22, 10.86)
Djibouti	6 (1, 13)	1.09 (0.12, 2.3)	182 (20, 401)	26.19 (2.89, 56.37)
Dominica	0 (0, 1)	0.37 (0.02, 0.79)	7 (0, 16)	8.3 (0.36, 17.84)
Dominican	19 (1, 41)	0.2 (0.02, 0.45)	468 (33, 1053)	4.87 (0.34, 10.94)
Ecuador	24 (4, 49)	0.17 (0.03, 0.35)	480 (84, 989)	3.2 (0.56, 6.6)
Egypt	2 (1, 3)	0 (0, 0.01)	58 (40, 79)	0.08 (0.06, 0.12)
El Salvador	9 (1, 18)	0.14 (0.01, 0.29)	176 (15, 379)	2.99 (0.26, 6.45)
Equatorial Guinea	4 (1, 9)	0.94 (0.15, 2.05)	107 (18, 246)	20.87 (3.46, 47.19)
Eritrea	39 (6, 80)	1.5 (0.25, 3.05)	1169 (199, 2395)	37.41 (6.22, 76.63)
Estonia	4 (0, 8)	0.14 (0.01, 0.33)	79 (4, 189)	3.48 (0.16, 8.47)
Eswatini	10 (2, 19)	1.71 (0.29, 3.36)	281 (47, 564)	43.96 (7.45, 87.77)
Ethiopia	119 (23, 245)	0.31 (0.06, 0.63)	3147 (597, 6430)	7.13 (1.36, 14.63)
Fiji	2 (0, 4)	0.27 (0.04, 0.56)	45 (5, 94)	5.79 (0.69, 12.06)
Finland	21 (1, 44)	0.17 (0.01, 0.35)	405 (18, 839)	3.67 (0.16, 7.67)
France	366 (18, 770)	0.28 (0.01, 0.58)	7536 (344, 15971)	6.48 (0.28, 13.69)
Gabon	11 (2, 23)	1.13 (0.18, 2.28)	302 (46, 615)	26.63 (4.13, 54.51)
Gambia	2 (0, 4)	0.24 (0.04, 0.46)	57 (9, 112)	5.64 (0.91, 11.15)
Georgia	6 (0, 14)	0.1 (0, 0.24)	145 (6, 332)	2.6 (0.11, 5.95)
Germany	435 (18, 927)	0.24 (0.01, 0.51)	9193 (355, 19665)	5.61 (0.21, 12.03)
Ghana	48 (6, 97)	0.31 (0.04, 0.64)	1266 (154, 2558)	7.38 (0.91, 15)
Greece	3 (1, 9)	0.01 (0, 0.03)	39 (12, 114)	0.16 (0.06, 0.43)
Greenland	0 (0, 1)	0.48 (0.03, 1.2)	8 (1, 21)	10.78 (0.67, 27.03)
Grenada	1 (0, 1)	0.67 (0.11, 1.29)	19 (3, 37)	16.15 (2.65, 31.23)
Guam	0 (0, 1)	0.22 (0.02, 0.46)	10 (1, 22)	5.37 (0.5, 11.21)
Guatemala	20 (2, 41)	0.19 (0.02, 0.4)	455 (43, 948)	3.99 (0.38, 8.33)
Guinea	10 (2, 21)	0.2 (0.03, 0.39)	275 (44, 544)	4.73 (0.75, 9.42)
Guinea-Bissau	5 (1, 10)	0.72 (0.11, 1.44)	144 (23, 296)	17.96 (2.85, 36.78)

Guyana	1 (0, 2)	0.18 (0.02, 0.38)	31 (3, 66)	4.55 (0.4, 9.56)
Haiti	28 (5, 58)	0.42 (0.07, 0.88)	755 (129, 1608)	9.95 (1.74, 21.01)
Honduras	9 (1, 18)	0.16 (0.02, 0.32)	210 (24, 431)	3.43 (0.4, 7.06)
Hungary	18 (1, 50)	0.1 (0.01, 0.27)	440 (32, 1211)	2.59 (0.2, 7.14)
Iceland	2 (0, 3)	0.29 (0.02, 0.59)	34 (2, 70)	6.57 (0.36, 13.62)
India	3114 (288, 6500)	0.28 (0.03, 0.58)	83426 (7353,	6.9 (0.61, 14.41)
Indonesia	442 (52, 898)	0.22 (0.03, 0.45)	11517 (1321,	4.98 (0.58, 10.16)
Iran (Islamic Republic of)	49 (7, 123)	0.08 (0.01, 0.2)	900 (147, 2306)	1.28 (0.2, 3.27)
Iraq	6 (1, 16)	0.03 (0, 0.08)	148 (15, 425)	0.62 (0.06, 1.74)
Ireland	24 (1, 56)	0.32 (0.01, 0.74)	457 (21, 1075)	6.23 (0.28, 14.66)
Israel	1 (0, 3)	0.01 (0, 0.02)	12 (7, 28)	0.1 (0.06, 0.23)
Italy	110 (6, 250)	0.07 (0, 0.17)	2006 (112, 4564)	1.58 (0.09, 3.59)
Jamaica	6 (0, 13)	0.21 (0.01, 0.44)	133 (8, 291)	4.51 (0.26, 9.87)
Japan	241 (29, 633)	0.06 (0.01, 0.17)	4108 (530, 10762)	1.4 (0.18, 3.65)
Jordan	3 (0, 8)	0.06 (0, 0.14)	91 (4, 213)	1.28 (0.06, 3.02)
Kazakhstan	9 (2, 24)	0.07 (0.02, 0.18)	166 (59, 451)	1.06 (0.34, 2.82)
Kenya	241 (27, 513)	1.19 (0.13, 2.54)	6551 (693, 13861)	27.3 (3.07, 58.06)
Kiribati	1 (0, 1)	0.81 (0.13, 1.66)	15 (2, 32)	19.66 (3.04, 40.94)
Kuwait	0 (0, 1)	0.02 (0, 0.06)	7 (1, 19)	0.29 (0.05, 0.82)
Kyrgyzstan	3 (0, 8)	0.07 (0.01, 0.21)	61 (9, 184)	1.4 (0.19, 4.12)
Lao People's Democratic Republic	8 (1, 18)	0.21 (0.02, 0.43)	228 (16, 490)	4.79 (0.35, 10.35)
Latvia	6 (0, 14)	0.15 (0.01, 0.36)	133 (6, 321)	3.93 (0.19, 9.53)
Lebanon	0 (0, 1)	0 (0, 0.01)	4 (2, 8)	0.08 (0.05, 0.15)
Lesotho	20 (3, 41)	1.64 (0.25, 3.26)	583 (86, 1160)	42.23 (6.34, 84.34)
Liberia	11 (2, 21)	0.56 (0.09, 1.09)	294 (46, 587)	13.08 (2.06, 25.87)
Libya	3 (0, 8)	0.07 (0, 0.16)	86 (4, 207)	1.56 (0.07, 3.72)
Lithuania	9 (0, 20)	0.16 (0.01, 0.39)	206 (10, 506)	4.2 (0.21, 10.4)
Luxembourg	2 (0, 4)	0.18 (0.01, 0.4)	38 (2, 87)	3.96 (0.18, 9.13)
Madagascar	122 (25, 235)	1.14 (0.23, 2.21)	3631 (733, 7107)	28.39 (5.74, 54.67)
Malawi	194 (39, 384)	2.71 (0.53, 5.3)	5484 (1087, 11043)	68.37 (13.74,
Malaysia	55 (5, 115)	0.22 (0.02, 0.46)	1300 (102, 2745)	4.74 (0.39, 10.01)
Maldives	0 (0, 1)	0.14 (0.01, 0.3)	9 (0, 20)	2.86 (0.14, 6.19)
Mali	23 (4, 44)	0.28 (0.05, 0.53)	634 (108, 1229)	6.8 (1.15, 13.08)
Malta	0 (0, 1)	0.04 (0, 0.11)	6 (1, 18)	0.73 (0.1, 2.08)
Marshall Islands	0 (0, 0)	0.3 (0.05, 0.61)	3 (0, 6)	7.08 (1.18, 14.24)
Mauritania	9 (1, 18)	0.47 (0.07, 0.93)	226 (34, 447)	10.54 (1.6, 20.66)
Mauritius	3 (0, 7)	0.18 (0.01, 0.39)	75 (4, 161)	4.18 (0.22, 9.01)
Mexico	132 (10, 270)	0.12 (0.01, 0.24)	3050 (218, 6247)	2.55 (0.18, 5.21)
Micronesia (Federated States of)	0 (0, 0)	0.34 (0.06, 0.69)	6 (1, 13)	8.08 (1.25, 16.93)
Monaco	0 (0, 0)	0.03 (0.01, 0.09)	1 (0, 2)	0.58 (0.21, 1.65)
Mongolia	40 (5, 80)	2.21 (0.29, 4.47)	947 (103, 1952)	42.97 (4.95, 86.84)

Montenegro	0 (0, 0)	0.01 (0, 0.02)	2 (1, 3)	0.18 (0.11, 0.34)
Morocco	14 (1, 36)	0.05 (0, 0.13)	326 (25, 871)	1.03 (0.08, 2.71)
Mozambique	93 (15, 177)	0.9 (0.15, 1.72)	2515 (418, 4871)	21.49 (3.59, 41.23)
Myanmar	81 (5, 173)	0.18 (0.01, 0.39)	2101 (124, 4483)	4.28 (0.26, 8.99)
Namibia	4 (1, 7)	0.27 (0.04, 0.54)	103 (16, 212)	6.81 (1.08, 13.8)
Nauru	0 (0, 0)	0.33 (0.05, 0.66)	0 (0, 1)	7.75 (1.19, 15.62)
Nepal	92 (9, 185)	0.43 (0.04, 0.87)	2333 (226, 4698)	10.05 (0.99, 20.34)
Netherlands	169 (8, 350)	0.49 (0.02, 1.01)	3359 (158, 6993)	10.49 (0.48, 21.9)
New Zealand	19 (1, 40)	0.23 (0.01, 0.5)	350 (15, 755)	4.64 (0.2, 10)
Nicaragua	6 (1, 11)	0.15 (0.02, 0.28)	130 (22, 250)	2.93 (0.49, 5.66)
Niger	24 (2, 51)	0.33 (0.03, 0.7)	627 (42, 1366)	7.58 (0.55, 16.32)
Nigeria	59 (4, 148)	0.08 (0.01, 0.18)	1488 (94, 3901)	1.65 (0.11, 4.15)
Niue	0 (0, 0)	0.25 (0.04, 0.49)	0 (0, 0)	5.58 (0.92, 10.94)
North Macedonia	0 (0, 1)	0.01 (0, 0.03)	5 (2, 15)	0.17 (0.07, 0.46)
Northern Mariana Islands	0 (0, 0)	0.28 (0.04, 0.56)	3 (0, 7)	6.02 (0.79, 12.28)
Norway	18 (1, 37)	0.18 (0.01, 0.38)	348 (23, 723)	3.88 (0.24, 8.05)
Oman	1 (0, 3)	0.1 (0.01, 0.24)	39 (2, 96)	2.08 (0.11, 4.98)
Pakistan	883 (137, 1742)	0.82 (0.13, 1.58)	25485 (3973,	20.39 (3.17, 40.44)
Palau	0 (0, 0)	0.24 (0.04, 0.48)	1 (0, 3)	5.64 (0.81, 11.28)
Palestine	1 (0, 3)	0.06 (0, 0.13)	28 (2, 66)	1.14 (0.07, 2.73)
Panama	7 (1, 13)	0.16 (0.02, 0.32)	146 (23, 297)	3.51 (0.55, 7.16)
Papua New	8 (1, 17)	0.19 (0.03, 0.41)	231 (31, 505)	4.37 (0.63, 9.45)
Paraguay	20 (3, 41)	0.37 (0.05, 0.75)	476 (62, 986)	8.39 (1.11, 17.34)
Peru	38 (4, 80)	0.12 (0.01, 0.25)	779 (80, 1644)	2.42 (0.25, 5.13)
Philippines	85 (7, 175)	0.11 (0.01, 0.23)	2352 (200, 4817)	2.75 (0.24, 5.6)
Poland	38 (5, 96)	0.05 (0.01, 0.13)	788 (107, 2051)	1.2 (0.17, 3.11)
Portugal	14 (1, 37)	0.06 (0.01, 0.16)	262 (33, 741)	1.26 (0.17, 3.64)
Puerto Rico	18 (3, 37)	0.25 (0.04, 0.5)	364 (62, 723)	5.64 (0.95, 11.26)
Qatar	0 (0, 0)	0.02 (0.01, 0.08)	2 (1, 5)	0.31 (0.13, 0.99)
Republic of Korea	52 (5, 137)	0.06 (0.01, 0.16)	915 (98, 2547)	1.02 (0.11, 2.83)
Republic of Moldova	6 (0, 14)	0.11 (0, 0.23)	168 (7, 371)	2.95 (0.13, 6.52)
Romania	2 (1, 3)	0.01 (0, 0.01)	49 (38, 66)	0.15 (0.12, 0.2)
Russian	416 (20, 921)	0.18 (0.01, 0.39)	10501 (516, 23632)	4.56 (0.22, 10.21)
Rwanda	66 (12, 134)	1.16 (0.21, 2.34)	1819 (324, 3713)	27.47 (5.01, 55.97)
Saint Kitts and Nevis	0 (0, 0)	0.38 (0.04, 0.74)	7 (1, 13)	8.97 (0.89, 17.74)
Saint Lucia	1 (0, 2)	0.44 (0.07, 0.84)	23 (4, 45)	10.59 (1.77, 20.18)
Saint Vincent and the Grenadines	0 (0, 1)	0.23 (0.03, 0.45)	8 (1, 15)	5.63 (0.76, 10.97)
Samoa	0 (0, 0)	0.19 (0.04, 0.35)	7 (1, 12)	4.35 (0.85, 8.19)
San Marino	0 (0, 0)	0.07 (0, 0.18)	1 (0, 2)	1.58 (0.07, 4.22)
Sao Tome and Principe	1 (0, 1)	0.52 (0.08, 1.03)	14 (2, 28)	12.15 (2, 24.3)
Saudi Arabia	16 (1, 37)	0.1 (0, 0.23)	495 (20, 1147)	2.28 (0.1, 5.2)

Senegal	31 (3, 64)	0.44 (0.05, 0.9)	801 (83, 1643)	10.21 (1.09, 20.88)
Serbia	18 (1, 43)	0.12 (0.01, 0.28)	424 (19, 1026)	2.91 (0.13, 7.07)
Seychelles	1 (0, 1)	0.54 (0.09, 1.05)	16 (3, 31)	13.53 (2.19, 25.84)
Sierra Leone	15 (2, 31)	0.45 (0.07, 0.92)	404 (61, 817)	10.6 (1.6, 21.72)
Singapore	5 (0, 11)	0.06 (0, 0.15)	90 (6, 234)	1.15 (0.08, 2.95)
Slovakia	14 (1, 34)	0.15 (0.01, 0.37)	353 (17, 865)	4.02 (0.19, 9.82)
Slovenia	7 (0, 15)	0.16 (0.01, 0.36)	150 (6, 339)	3.92 (0.16, 8.95)
Solomon Islands	1 (0, 2)	0.4 (0.07, 0.77)	38 (6, 77)	10.4 (1.79, 20.43)
Somalia	100 (18, 204)	1.54 (0.27, 3.14)	3002 (528, 6073)	39.35 (6.97, 79.8)
South Africa	402 (51, 796)	0.95 (0.12, 1.86)	10138 (1249,	21.63 (2.7, 42.88)
South Sudan	40 (6, 82)	1.09 (0.18, 2.26)	1112 (177, 2307)	26 (4.16, 53.57)
Spain	94 (5, 224)	0.1 (0.01, 0.24)	1912 (108, 4682)	2.28 (0.13, 5.64)
Sri Lanka	98 (11, 203)	0.4 (0.05, 0.82)	2316 (247, 4875)	8.86 (0.94, 18.63)
Sudan	62 (3, 153)	0.36 (0.02, 0.88)	1575 (80, 3843)	8.01 (0.42, 19.67)
Suriname	1 (0, 2)	0.13 (0.02, 0.26)	20 (2, 40)	3.11 (0.35, 6.29)
Sweden	40 (2, 85)	0.18 (0.01, 0.39)	735 (35, 1540)	3.82 (0.18, 8.01)
Switzerland	38 (2, 81)	0.22 (0.01, 0.46)	749 (31, 1602)	4.7 (0.19, 10.12)
Syrian Arab Republic	7 (0, 16)	0.06 (0, 0.14)	180 (8, 419)	1.38 (0.06, 3.14)
Taiwan (Province of China)	82 (6, 223)	0.21 (0.02, 0.57)	2143 (169, 5899)	5.65 (0.45, 15.65)
Tajikistan	5 (1, 17)	0.15 (0.01, 0.47)	128 (16, 427)	2.74 (0.3, 8.9)
Thailand	273 (30, 577)	0.27 (0.03, 0.56)	6992 (747, 15118)	6.68 (0.72, 14.4)
Timor-Leste	2 (0, 4)	0.26 (0.05, 0.53)	51 (9, 104)	6.05 (1.1, 12.26)
Togo	19 (3, 37)	0.54 (0.09, 1.05)	528 (82, 1051)	12.91 (2.02, 25.64)
Tokelau	0 (0, 0)	0.2 (0.04, 0.4)	0 (0, 0)	4.49 (0.79, 9.01)
Tonga	0 (0, 0)	0.23 (0.04, 0.45)	4 (1, 8)	5.1 (0.92, 9.83)
Trinidad and	3 (0, 6)	0.15 (0.02, 0.3)	67 (9, 138)	3.61 (0.5, 7.43)
Tunisia	2 (0, 5)	0.02 (0, 0.05)	35 (5, 105)	0.29 (0.04, 0.85)
Turkey	3 (2, 4)	0 (0, 0)	70 (53, 88)	0.08 (0.06, 0.1)
Turkmenistan	1 (1, 3)	0.04 (0.02, 0.08)	28 (18, 52)	0.71 (0.42, 1.42)
Tuvalu	0 (0, 0)	0.26 (0.04, 0.5)	1 (0, 1)	5.98 (1.04, 11.79)
Uganda	237 (41, 463)	1.76 (0.31, 3.41)	6642 (1152, 13260)	42.65 (7.42, 84)
Ukraine	32 (4, 94)	0.04 (0.01, 0.13)	819 (112, 2494)	1.16 (0.16, 3.57)
United Arab Emirates	16 (1, 47)	0.4 (0.01, 1.19)	599 (23, 1685)	9.18 (0.35, 27.15)
United Kingdom	644 (42, 1340)	0.49 (0.03, 1.03)	11804 (725, 24644)	10.03 (0.6, 21.01)
United Republic of Tanzania	290 (50, 600)	1.23 (0.22, 2.5)	7890 (1283, 16763)	29.59 (4.98, 61.66)
United States of America	1216 (97, 2491)	0.22 (0.02, 0.44)	25618 (1988, 52818)	4.81 (0.37, 9.92)
United States Virgin Islands	1 (0, 1)	0.32 (0.02, 0.66)	14 (1, 28)	7.59 (0.5, 15.71)
Uruguay	30 (3, 59)	0.53 (0.05, 1.06)	583 (55, 1164)	11.57 (1.1, 23.09)
Uzbekistan	3 (2, 3)	0.02 (0.01, 0.04)	79 (65, 94)	0.37 (0.29, 0.52)
Vanuatu	0 (0, 1)	0.28 (0.05, 0.56)	12 (2, 25)	6.58 (1.11, 13.44)

Venezuela (Bolivarian Republic of)	49 (4, 103)	0.17 (0.02, 0.36)	1153 (99, 2444)	3.87 (0.34, 8.19)
Viet Nam	136 (7, 319)	0.15 (0.01, 0.34)	3617 (180, 8796)	3.56 (0.17, 8.46)
Yemen	54 (7, 116)	0.44 (0.06, 0.96)	1445 (171, 3155)	10.03 (1.21, 21.64)
Zambia	103 (17, 200)	1.58 (0.26, 3.04)	3028 (484, 5913)	39.64 (6.33, 77.07)
Zimbabwe	125 (22, 233)	1.84 (0.33, 3.44)	3543 (635, 6689)	45.9 (8.25, 85.88)
